# GPR50 regulates neuronal development as a mitophagy receptor

**DOI:** 10.1038/s41419-024-06978-y

**Published:** 2024-08-15

**Authors:** Ji-Chuan Liu, Xiu-Yun Zhao, Ming-Lei Wu, Yi-fan Shi, Ze-Ping Huang, Li-Pao Fang, Chao Zhu, Xuan Peng, Zi-Ling Shi, Li-Jun Lan, Wen-Li Ji, Li Luo, Lei Feng, Zeng-Li Zhang, De-en Xu, Shao Li, Zheng-Hong Qin, Yan-Yun Sun, Melitta Schachner, Quan-Hong Ma

**Affiliations:** 1 Department of Neurology and Clinical Research Center of Neurological Disease, the Second Affiliated Hospital of Soochow University, Suzhou, Jiangsu 215021 China; 2 Institute of Neuroscience & Jiangsu Key Laboratory of Translational Research and Therapy for Neuro-psycho-Diseases, Soochow University, Suzhou, Jiangsu 215021 China; 3 Department of Physiology, College of Basic Medical Sciences, Liaoning Provincial Key Laboratory of Cerebral Diseases, National-Local Joint Engineering Research Center for Drug-Research and Development (R & D) of Neurodegenerative Diseases, Dalian Medical University Dalian, 116044 China; 4 School of Public Health, Soochow University, Suzhou, Jiangsu 215021 China; 5 School of Physical Education and Sports Science, Soochow University, Suzhou, 215021 China; 6 Monash Suzhou Research Institute, Suzhou, 215000 China; 7 The Wuxi No.2 People Hospital, Wuxi, 214002 Jiangsu China; 8 Institute of Health Technology, Suzhou Gaobo Vocational College, Suzhou High-Technology District, Science & Technology Town, 5 Qingshan Road, Suzhou, Jiangsu 215163 PR China; 9 Center for Neuroscience, Shantou University Medical College, Shantou, Guangdong, 515041 China; 10 Keck Center for Collaborative Neuroscience and Department of Cell Biology and Neuroscience, Rutgers University, Piscataway, NJ 08854 USA

**Keywords:** Molecular neuroscience, Disease model

## Abstract

Neurons rely heavily on high mitochondrial metabolism to provide sufficient energy for proper development. However, it remains unclear how neurons maintain high oxidative phosphorylation (OXPHOS) during development. Mitophagy plays a pivotal role in maintaining mitochondrial quality and quantity. We herein describe that G protein-coupled receptor 50 (GPR50) is a novel mitophagy receptor, which harbors the LC3-interacting region (LIR) and is required in mitophagy under stress conditions. Although it does not localize in mitochondria under normal culturing conditions, GPR50 is recruited to the depolarized mitochondrial membrane upon mitophagy stress, which marks the mitochondrial portion and recruits the assembling autophagosomes, eventually facilitating the mitochondrial fragments to be engulfed by the autophagosomes. Mutations Δ502-505 and T532A attenuate GPR50-mediated mitophagy by disrupting the binding of GPR50 to LC3 and the mitochondrial recruitment of GPR50. Deficiency of GPR50 causes the accumulation of damaged mitochondria and disrupts OXPHOS, resulting in insufficient ATP production and excessive ROS generation, eventually impairing neuronal development. GPR50-deficient mice exhibit impaired social recognition, which is rescued by prenatal treatment with mitoQ, a mitochondrially antioxidant. The present study identifies GPR50 as a novel mitophagy receptor that is required to maintain mitochondrial OXPHOS in developing neurons.

## Introduction

Mitochondria, as the powerhouse of eukaryotic cells, supply neurons with energy by generating metabolites via the tricarboxylic acid (TCA) cycle and ATP through OXPHOS. Neurons, characterized by their high energy demands, heavily rely on ATP provided by mitochondria [1]. Mitochondrial metabolism controls the species-specific tempo of cortical neurons. The prolonged human neuronal development correlates with lower mitochondrial metabolic activity, particularly that of OXPHOS. Enhancing mitochondrial oxidative metabolism accelerates human neuronal development, further emphasizing the essential role of OXPHOS in neuronal development [2]. Neurons cannot generate ATP through glycolysis, making them more susceptible to mitochondrial dysfunction [3]. Mitochondrial dysfunction results in insufficient ATP supply, oxidative stress, and impaired signaling pathways, which have been linked to neurodevelopmental disorders such as autism spectrum disorders (ASD). The latter is clinically manifested with impaired social capability and repetitive and stereotyped behaviors [4, 5]. Mitochondrial dysfunction and elevated ROS levels are even considered hallmarks of ASD [6–9]. However, how neurons maintain high mitochondrial OXPHOS to provide sufficient energy for proper development remains unclear.

Mitophagy, a subtype of selective autophagy, is pivotal in maintaining mitochondrial quality and quantity. Mitophagy relies on a population of factors called mitophagy receptors to recruit the assembling autophagosomes (phagophores) adjacent to the damaged mitochondria. With the fusion of autophagosomes to lysosomes, the engulfed mitochondria are degraded. Mitophagy receptors usually contain a conserved LC3 or GABARAP interaction region (LIR or GIM, respectively), through which mitophagy receptors bind to LC3 and recruit the assembling autophagosomes. The LIR/GIM motif is characterized by conserved “W/F/Y-x-x-L/I/V” motifs surrounded by acidic amino acids in the cytoplasmic domain [3]. Intriguingly, mitophagy is broadly activated in metabolically enhanced neurons. OXPHOS stimulation depolarizes the mitochondrial membrane and enhances mitophagy. These data indicate that to sustain high energetic activity, neurons must increase mitochondrial turnover and, hence, facilitate mitochondrial maintenance [3]. However, how mitophagy is coordinated with mitochondrial OXPHOS to control neuronal development remains unknown.

G protein-coupled receptor 50 (GPR50), an X-chromone-linked orphan GPCR, shares approximately 45% of its amino acid sequence homology with the melatonin receptors MT1 and MT2. However, GPR50 does not bind to melatonin or any other ligand, therefore remaining an orphan receptor [10]. Two GPR50 variations, Δ502-505 and T532A, have been detected in patients with ASD [11, 12], bipolar disorder, and major depression [13–15]. However, limited information is known about the role of GPR50 in the brain. Knocking down GPR50 in vitro decreases the proliferation and differentiation of neural progenitor cells and neurite outgrowth [16, 17], suggesting that GPR50 plays a role in maintaining neuronal development and function. We herein describe GPR50 as a novel mitophagy receptor containing an LIR and binding to LC3. GPR50 is recruited to the depolarized mitochondrial membrane upon mitophagy stress, where it marks the mitochondrial portion for degradation and recruits the assembling autophagosomes, facilitating the mitochondrial portion to be engulfed by the autophagosomes. We further show that disease-related mutations at Δ502-505 and T532A impair GPR50-mediated mitophagy by attenuating the binding of GPR50 to LC3 and the mitochondrial recruitment of GPR50. GPR50 deficiency causes accumulation of the damaged mitochondria, impairing mitochondrial OXPHOS, resulting in insufficient ATP production and excessive ROS accumulation, eventually leading to defective neuronal development. GPR50-deficient mice exhibit impaired social recognition, which is rescued by prenatal treatment with mitoQ, a mitochondrially antioxidant. Thus, GPR50, as a mitophagy receptor, is required for neuronal development via maintaining mitochondrial OXPHOS.

## Results

### GPR50 is transiently recruited to the mitochondrial region upon mitochondrial membrane depolarization

**Fig. 1:**
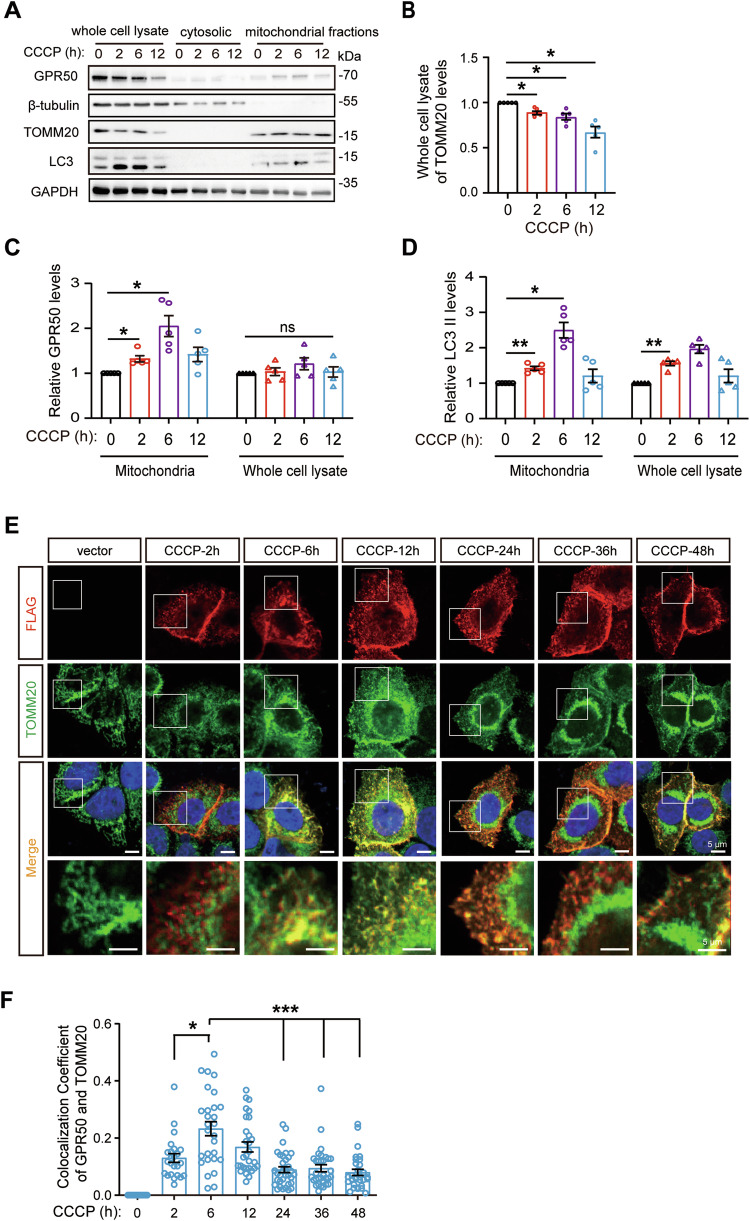
GPR50 is transiently recruited to the mitochondrial region upon mitochondrial membrane depolarization. **A**–**D** Western blotting analysis of endogenous GPR50, TOMM20, and LC3 II levels in the whole cell lysate and mitochondria fractions of Hela cells treated with CCCP for a distinct time as indicated. **B** Relative levels of TOMM20 in the whole cell lysate. One-way ANOVA (F_3, 16_ = 13.769, *p* = 0.000) followed by Dunnett T3 post hoc tests for CCCP 0 h versus 2 h (*p* = 0.025), 0 h versus 6 h (*p* = 0.045), 0 h versus 12 h (*p* = 0.026). *n* = 5 independent experiments (**B**). **C**, **D** Relative levels of GPR50 and LC3-II in the whole cell lysate and mitochondria fractions. One-way ANOVA for Mitochondria fractions (F_3, 16_ = 9.110, *p* = 0.001) followed by Dunnett T3 post hoc tests for 0 h versus 2 h (*p* = 0.042), 0 h versus 6 h (*p* = 0.045). One-way ANOVA for whole cell lysate (F_3, 16_ = 0.960, *p* = 0.436) followed by Dunnett T3 post hoc tests for CCCP 0 h versus 2 h (*p* = 0.998), 0 h versus 6 h (*p* = 0.581), 0 h versus 12 h (*p* = 1.000) (**C**). One-way ANOVA for Mitochondria fractions (F_3, 16_ = 20.603, *p* = 0.000) followed by Dunnett T3 post hoc tests for 0 h versus 2 h (*p* = 0.008), 0 h versus 6 h (*p* = 0.010). One-way ANOVA for whole cell lysate (F_3, 16_ = 9.132, *p* = 0.001) followed by Dunnett T3 post hoc tests for 0 h versus 2 h (*p* = 0.004) (**D**). *n* = 5 independent experiments (**C**, **D**). **E**, **F** HeLa cells transfected with GPR50-FLAG WT were treated with CCCP for the indicated time and immunostained for FLAG, TOMM20, and DAPI (E). Scale bars: 5 μm. **F** Colocalization coefficient between GPR50 and TOMM20. One-way ANOVA (F_6, 186_ = 19.451, *p* = 0.000) followed by Dunnett T3 post hoc tests for CCCP 2 h versus 6 h (*p* = 0.019), 6 h versus 24 h (*p* = 0.000), 6 h versus 36 h (*p* = 0.000), 6 h versus 48 h (*p* = 0.000). *n* = 16–34 cells per group from 3 independent experiments (**F**). Data are presented as mean ± SEM. *p < 0.05; **p < 0.01; ***p < 0.001. n.s, non-significance.

We failed to detect the distribution of GPR50 in the mitochondria in HEK293 cells under standard culturing conditions using immunofluorescence staining (Supplementary Fig. 1A, B). To mimic the mitophagy stress during OXPHOS stimulation [3], we treated HEK293 cells with carbonyl cyanide m-chlorophenyl hydrazone (CCCP) (Fig. 1). This mitochondrial uncoupler induces mitochondrial depolarization and mitophagy in a time-dependent manner [18, 19]. TOMM20 levels (a mitochondrial outer membrane protein) and LC3-II in the whole cell lysates (Fig. 1A, B, D; Supplementary Fig. 4) were decreased and increased respectively from 2 h of CCCP treatment. The levels of LC3-II in the mitochondrial fraction, which marks the autophagosomes/phagophores recruited by mitochondria, were increased from 2 h to reach a peak of 6 h of CCCP treatment (Fig. 1A, D). We were surprised to observe a transiently and dynamic mitochondrial distribution of GPR50 upon CCCP treatment, which displays a synchronous pattern with CCCP-induced mitophagy. The endogenous GPR50 in the mitochondrial fraction accumulated from 2 h to peak at 6 hours and declined from 12 h of CCCP treatment (Fig. 1A, C). Coimmunostaining for FLAG and TOMM20 in HeLa cells showed similar results (Fig. 1E, F). These results indicate that GPR50 is transiently recruited to the mitochondrion upon the depolarization of the mitochondrial membrane. The high synchronicity between the mitochondrial recruitment of GPR50 and autophagosomes/phagophores suggests a role of GPR50 in mitophagy.

### GPR50 binds to LC3 via the LIR motif, which is attenuated by ASD mutations

**Fig. 2:**
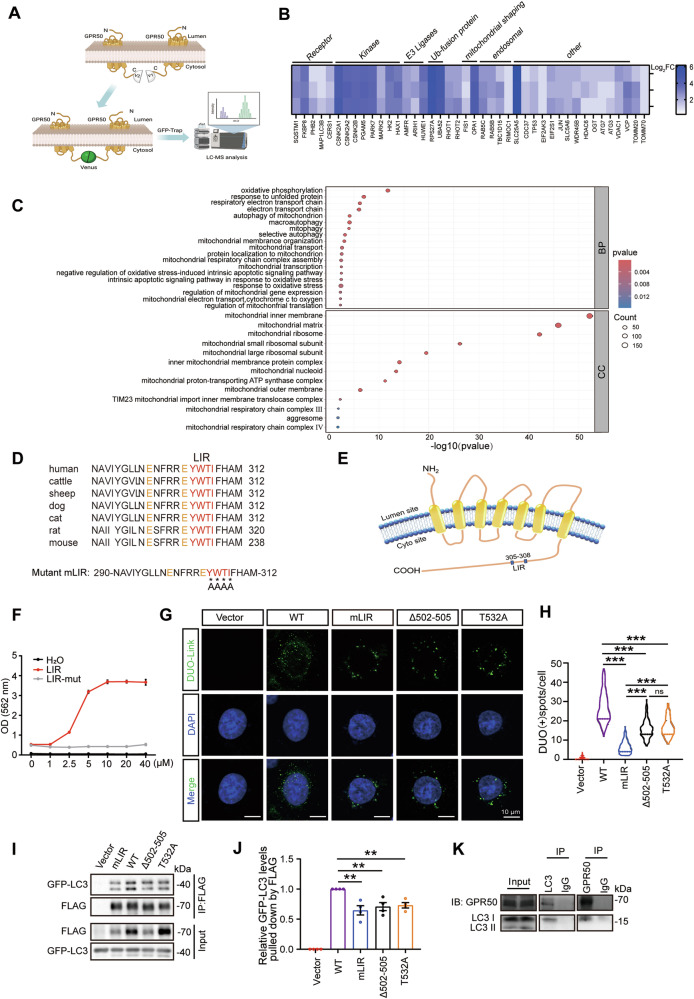
GPR50 binds to LC3 via the LIR motif, which is attenuated by ASD mutations. **A** BiCAP interactome analysis of GPR50-V1 and GPR50-V2 homodimers after GFP-Trap for LC-MS. **B** The heat map shows the interaction affinity of proteins enriched in “mitophagy” from the Go analysis. **C** Analysis of cellular components and biological processes of GPR50 interaction partners by Gene ontology (Go). **D** The conserved sequence of peptides containing the LIR motif (Y^305^WTI^308^) and its mutation sites. **E** Schematic description of the location of LIR Y^305^WTI^308^ in GPR50. **F** ELISA analysis of the binding of WT or mutant LIR motif containing-peptides to recombinant LC3. **G** Proximity Ligation Assay for HeLa cells transfected with either GPR50-FLAG WT, mLIR, Δ502-505, or T532A using antibodies against LC3 and FLAG. Scale bars: 10 μm. **H** Numbers of DUOLink^+^ puncta per cell were quantified. One-way ANOVA (F_4, 980_ = 922.910, *p* = 0.000) followed by Dunnett T3 post hoc tests for GPR50 WT versus mLIR (*p* = 0.000), GPR50 WT versus GPR50 Δ502-505 (*p* = 0.000), GPR50 WT versus GPR50 T532A (*p* = 0.000), mLIR versus GPR50 Δ502-505 (*p* = 0.000), mLIR versus GPR50 T532A (*p* = 0.000), GPR50 Δ502-505 versus GPR50 T532A (*p* = 0.395). *n* = 178–208 cells per group from 3 independent experiments (**G**). **I** Co-immunoprecipitation in HEK293T cells co-transfected with GFP-LC3 and GPR50-FLAG WT, mLIR, Δ502-505 or T532A. Cell lysates were immunoprecipitated with anti-FLAG and detected with antibodies against GFP and FLAG. **J** The relative levels of GFP-LC3 pulled down by GPR50-FLAG were quantified. One-way ANOVA (F_3, 12_ = 7.817, *p* = 0.004) followed by LSD post hoc tests for GPR50 WT versus mLIR (*p* = 0.001), GPR50 WT versus GPR50 Δ502-505 (*p* = 0.003), GPR50 WT versus GPR50 T532A (*p* = 0.005). *n* = 4 independent experiments (**I**). **K** Lysates of adult mouse brains were immunoprecipitated with antibodies against endo GPR50 and LC3 and detected with antibodies against GPR50 and LC3, respectively. Data are presented as mean ± SEM. *p < 0.05; **p < 0.01; ***p < 0.001. n.s, non-significance.

Autophagic receptors necessitate dimerization or oligomerization for optimal functionality in selective autophagy. Dimerization or oligomerization increases the concentration of autophagy receptors in the local membrane, enhancing the binding affinity of these factors to LC3 or membranes, thus facilitating their function in membrane curvature and recruiting phagophores [20]. Thus, to identify the molecular mechanism underlying GPR50-mediated mitochondrial function, we used biomolecular complementation affinity purification (BiCAP) to screen the factors potentially interacting with GPR50 homodimers. The carboxyl terminus of GPR50 was linked to the V1 or V2 segment of the fluorescent Venus protein. The GPR50-V1 and GPR50-V2 homodimers were immunoprecipitated and subjected to liquid chromatography and mass spectrometry (LC-MS) analysis (Fig. 2A). The interaction partners of GPR50 homodimers were subjected to Gene ontology (Go) analysis. The cellular components from Go terms showed an extensive location in the mitochondrial membrane, matrix, ribosomes, and mitochondrial respiratory complex, which further confirms a distribution of GPR50 in mitochondria. The biological processes from Go terms include pathways related to OXPHOS, autophagy, mitophagy, and responses to oxidative stress (Fig. 2C). We observed an extensive interaction of GPR50 with various mitophagy regulators including mitophagy receptors (FKBP8, PHB2, CERS1) and autophagy receptor (SQSTM1), kinases that modulate phosphorylation (PGAM5, PARK7, CSNK2, MARK2, HK2) and ubiquitination (AMFR, HUWE1) of mitophagy receptors, mitochondrial shaping proteins (FIS1 and OPA1). Of note, LC3 was also identified as one of the binding partners of GPR50 (Fig. 2B). Because GPR50 forms a protein complex with various mitophagy regulators, we were curious whether GPR50 directly binds to LC3. Autophagy receptors are characterized by a conserved “W/F/Y-x-x-L/I/V” motif surrounded by acidic amino acids [21]. Analysis of the sequence of GPR50 protein by iLIR [22] indicates that GPR50 contains one conserved “W/F/Y-x-x-L/I/V” motif (LIR, Y^305^WTI^308^) in its cytoplasmic domains (Fig. 2D, E). To examine whether a direct binding exists between Y^305^WTI^308^ and LC3, we performed ELISA by using recombinant LC3 protein coated on the dish plate to bait biotin-labeled synthetic peptides comprising of LIR, e.g., a.a.290-312. The results showed that LIR peptides bound to recombinant LC3 in a dose-dependent manner, which was abolished by mutating Y^305^WTI^308^ into quadruple alanine (Fig. 2D, F). The PLA-ligation assay in HeLa cells transfected with WT-GPR50-FLAG further showed that GPR50 interacts with endogenous LC3 (Fig. 2G, H). Co-IP analysis with the lysates from the cells cotransfected with GPR50-FLAG and LC3-GFP observed that both LC3-I and its lipidated form, LC3-II [23], were immunoprecipitated by GPR50-FLAG (Fig. 2I, J; Supplementary Fig. 4). The endogenous interaction between GPR50 and LC3 was further confirmed by the Co-IP assay with lysates from mouse brains (Fig. 2K; Supplementary Fig. 4). Both Co-IP and the duolink assay showed that mutation on the Y^305^WTI^308^ motif attenuated the binding of GPR50 to LC3, indicating that Y^305^WTI^308^ is one of the LIR motifs in GPR50 (Fig. 2F–J).

Intriguingly, the T532A mutation (532^Threonine^ is mutated to 532^Alanine^) and the GPR50 truncation (amino acid 502-505 is deleted, Δ502-505), which are genetically associated with ASD, bipolar disorder, and depression [11–15], also showed an attenuated binding capability to LC3 in both the duolink assay (Fig. 2G, H) and the Co-IP (Fig. 2I, J; Supplementary Fig. 4).

### Mitochondrial recruitment of GPR50 does not depend on its binding to LC3

**Fig. 3:**
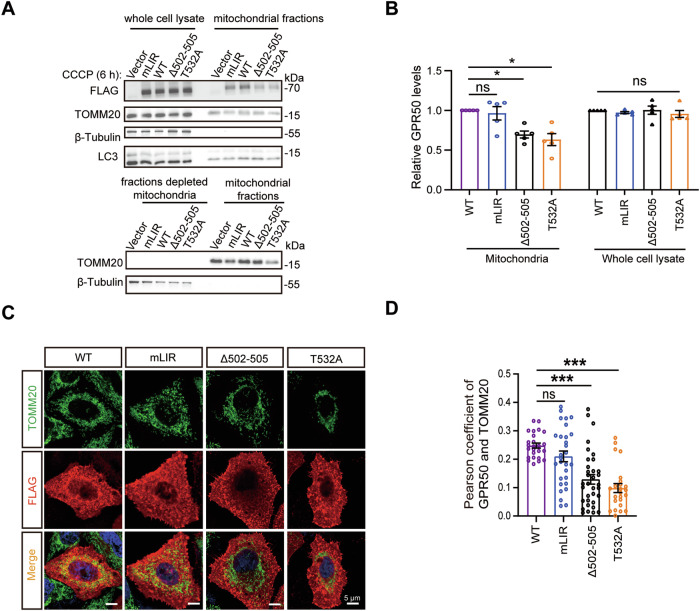
Mitochondrial recruitment of GPR50 does not depend on its binding to LC3. **A**, **B** Western blotting analysis of GPR50 levels with anti-FLAG antibodies in the whole cell lysates and mitochondria fractions of HeLa cells that were transfected with GPR50-FLAG WT, mLIR, Δ502-505, or T532A and treated with CCCP (50 μM) for 6 h (**A**). **B** Relative levels of GPR50 in the whole cell lysate and mitochondria fractions. One-way ANOVA for whole cell lysates (F_3, 16_ = 0.435, *p* = 0.731) followed by LSD post hoc tests for WT versus mLIR (*p* = 0.571), WT versus Δ502-505 (*p* = 0.955), WT versus T532A (*p* = 0.378) (**B**). One-way ANOVA for mitochondrial fractions (F_3, 16_ = 9.469, *p* = 0.001) followed by Dunnett T3 post hoc tests for WT versus mLIR (*p* = 0.997), WT versus Δ502-505 (*p* = 0.010), WT versus T532A (*p* = 0.035). *n* = 5 independent experiments (**B**). **C**, **D** HeLa cells transfected with GPR50-FLAG WT, mLIR, Δ502-505, or T532A were immunostained for FLAG and TOMM20 (**C**). Scale bars: 5 μm. Pearson coefficient of TOMM20 and GPR50 (**D**). One-way ANOVA (F_3, 108_ = 17.003, *p* = 0.000) followed by Dunnett T3 post hoc tests for WT versus mLIR (*p* = 0.378), WT versus Δ502-505 (*p* = 0.000), WT versus T532A (*p* = 0.000). *n* = 24–34 cells per group from 3 independent experiments (**D**). Data are presented as mean ± SEM.*p < 0.05; **p < 0.01; ***p < 0.001. n.s, non-significance.

We further examined whether the mitochondrial recruitment of GPR50 relies on its binding to LC3. HeLa cells were transfected with WT GPR50, GPR50^T532A^, GPR50^Δ502-505^, or mLIR plasmids, which were fused a FLAG tag in their carboxyl terminus and treated with CCCP. WB analysis with an anti-FLAG antibody showed that compared to WT GPR50, the levels of GPR50^T532A^ and GPR50^Δ502-505^, but not those of GPR50^mLIR^, were decreased in the mitochondrial fraction in CCCP-treated HeLa cells, while their levels in the whole cell lysates remained comparable to GPR50 WT (Fig. 3A, B; Supplementary Fig. 4). Coimmunostaining analysis revealed that in comparison to WT GPR50, GPR50^T532A^ and GPR50^Δ502-505^, but not those of GPR50^mLIR^, displayed decreased colocalization with TOMM20 in CCCP-treated HeLa cells (Fig. 3C, D). Therefore, these results indicate the mitochondrial recruitment of GPR50 does not rely on its binding to LC3. In contrast, ASD mutations impair the mitochondrial recruitment of GPR50 in a mechanism independent of binding to LC3.

### GPR50 is required in CCCP-induced mitophagy in an LC3-binding dependent manner

**Fig. 4:**
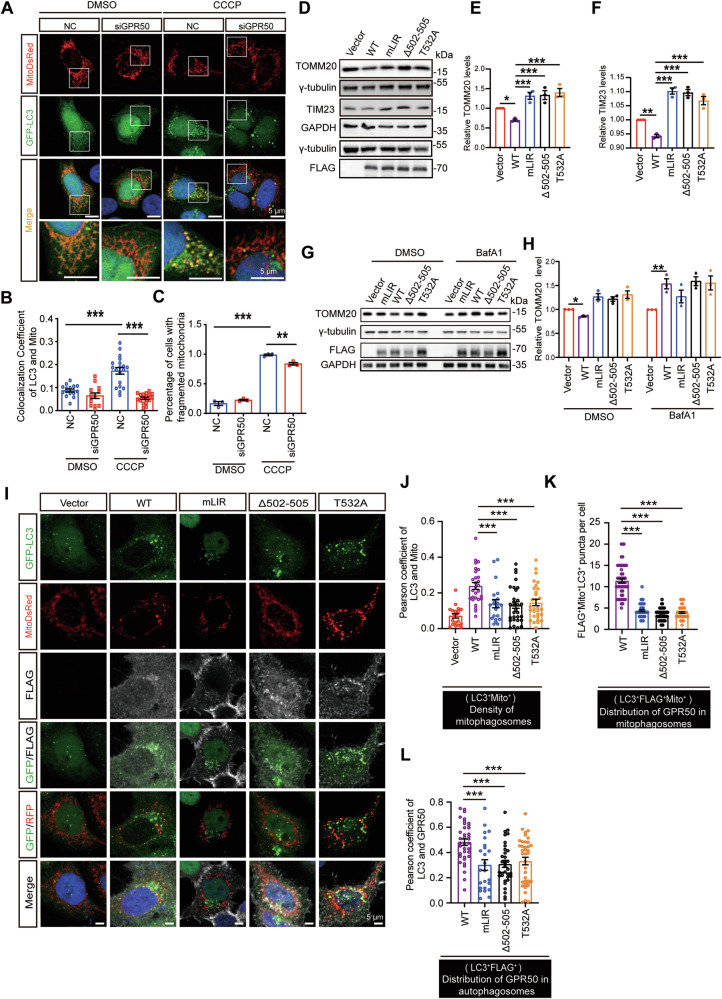
GPR50 is required in CCCP-induced mitophagy in an LC3-binding dependent manner. **A**–**C** HeLa cells co-transfected with mitoDsRed, LC3-GFP, and either GPR50 siRNA or scramble siRNA (NC) were treated with DMSO or CCCP and imaged (**A**). Scale bars: 5 μm. Colocalization Coefficient of mitoDsRed and LC3-GFP (**B**). Percentage of cells with fragmented mitochondria (**C**). One-way ANOVA (F_3, 60_ = 27.711, *p* = 0.000) followed by Dunnett T3 post hoc tests for NC-DMSO versus NC-CCCP (*p* = 0.000), NC-CCCP versus siGPR50-CCCP (*p* = 0.000). *n* = 15–19 cells per group from 3 independent experiments (**B**). One-way ANOVA (F_3, 8_ = 367.848, *p* = 0.000) followed by LSD post hoc tests for NC-DMSO versus NC-CCCP (*p* = 0.000), NC-CCCP versus siGPR50-CCCP (*p* = 0.001). *n* = 3 independent experiments (**C**). **D**–**H** Western blotting analysis of TOMM20 and TIM23 levels in GPR50-FLAG WT, mLIR, Δ502-505, or T532A transfected in HeLa cells (**D**–**F**), which were treated with Baf-A1 (**G**, **H**). Relative levels of TOMM20 (**E**, **H**) and TIM23 (**F**). One-way ANOVA (F_4, 10_ = 13.769, *p* = 0.000) followed by LSD post hoc tests for Vector versus WT (*p* = 0.023), WT versus mLIR (*p* = 0.000), WT versus Δ502-505 (*p* = 0.000), WT versus T532A (*p* = 0.000) (**E**). One-way ANOVA (F_4, 10_ = 49.733, *p* = 0.000) followed by LSD post hoc tests for Vector versus WT (*p* = 0.002), WT versus mLIR (*p* = 0.000), WT versus Δ502-505 (*p* = 0.000), WT versus T532A (*p* = 0.000) (**F**). One-way ANOVA for DMSO treatment (F_4, 10_ = 14.277, *p* = 0.000) followed by Dunnett T3 post hoc tests for Vector versus WT (*p* = 0.028), WT versus mLIR (*p* = 0.080), WT versus Δ502-505 (*p* = 0.050), WT versus T532A (*p* = 0.108) (**H**). One-way ANOVA for Baf-A1 treatment (F_4, 10_ = 5.414, *p* = 0.014) followed by LSD post hoc tests for Vector versus WT (*p* = 0.006), WT versus mLIR (*p* = 0.115), WT versus Δ502-505 (*p* = 0.750), WT versus T532A (*p* = 0.915) (**H**). *n* = 3 independent experiments (**E**, **F**, **H**). **I**–**L** HeLa cells co-transfected with GPR50-FLAG WT, mLIR, Δ502-505 or T532A, mitoDsRed, and LC3-GFP were immunostained for FLAG (**I**). Scale bars: 5 μm. Pearson coefficient of LC3-GFP and mitoDsRed (**J**). FLAG^+^Mito^+^LC3^+^ puncta per cell (**K**). Pearson coefficient of LC3-GFP and GPR50 (**L**). One-way ANOVA (F_4, 123_ = 9.634, *p* = 0.000) followed by LSD post hoc tests for WT versus mLIR (*p* = 0.000), WT versus Δ502-505 (*p* = 0.000), WT versus T532A (*p* = 0.000). *n* = 19–30 cells per group from 3 independent experiments (**J**). One-way ANOVA (F_3, 207_ = 149.999, *p* = 0.014) followed by Dunnett T3 post hoc tests for WT versus mLIR (*p* = 0.000), WT versus Δ502-505 (*p* = 0.000), WT versus T532A (*p* = 0.000). *n* = 52-53 cells per group from 3 independent experiments (**K**). One-way ANOVA (F_3, 129_ = 8.050, *p* = 0.000) followed by LSD post hoc tests for WT versus mLIR (*p* = 0.000), WT versus Δ502-505 (*p* = 0.000), WT versus T532A (*p* = 0.000). *n* = 24–38 cells per group from 3 independent experiments (**L**). Data are presented as mean ± SEM.*p < 0.05; **p < 0.01; ***p < 0.001. n.s, non-significance.

Because the mitochondrial recruitment of GPR50 is highly synchronous to that of autophagosomes and GPR50 contains LIR, we wondered whether GPR50 is a novel mitophagy receptor. HeLa cells cotransfected with either GPR50 siRNA or a scrambled siRNA (NC) (Supplementary Fig. 2A; Supplementary Fig. 5), GFP-LC3, and mitoDsRed were treated with CCCP to induce mitophagy (Fig. 4A). GPR50 siRNA abolished the CCCP-induced accumulation of mitophagosome (LC3^+^mitoDsRed^+^ puncta) (Fig. 4A, B), while it attenuated the CCCP-induced mitochondrial fragmentation (Fig. 4A, C), indicating that GPR50 is required in the CCCP-induced mitophagy. Notably, the knocking-down of GPR50 failed to affect the mitophagy in control cells treated with DMSO (Fig. 4A–C), suggesting that GPR50 mediates mitophagy upon mitochondrial depolarization rather than consecutively.

We also observed that the overexpression of GPR50 is sufficient to induce mitophagy in HeLa cells by using a similar experimental approach (Supplementary Fig. 2B–D). Overexpression of GPR50 reduced the protein levels of mitochondrial proteins, including TOMM20 (a mitochondrial preprotein translocase anchored in the outer membrane), TIM23 (a mitochondrial preprotein translocase anchored in the inner membrane), and MTCO2 (one of the components of the cytochrome c oxidase) in HeLa cells, which was prevented by the treatment with bafilomycin A1 (Fig. 4D–H; Supplementary Fig. 5; Supplementary Fig. 2E–J; Supplementary Fig. 5), an inhibitor of the lysosomal proton pump to inhibit lysosomal acidification and to block the fusion of autophagosomes to lysosomes [24]. In contrast, overexpression of GPR50 failed to alter the levels of *Mtco2* mRNA (Supplementary Fig. 2H), further confirming a posttranscriptional mechanism involved.

In contrast to WT GPR50, overexpression of GPR50^mLIR^, GPR50^T532A^, or GPR50^Δ502-505^, all of which exhibited an attenuated binding to LC3, failed to decrease the levels of mitochondrial proteins in HeLa cells, despite the presence or absence of bafilomycin A1 (Fig. 4D–H; Supplementary Fig. 2K; Supplementary Fig. 5). Immunofluorescence analysis was performed in HeLa cells that were cotransfected with LC3-GFP, mitoDsRed, and WT-GPR50 or GPR50^mLIR^, GPR50^T532A^, GPR50^Δ502-505^ mutants. Compared to the vector, transfection of WT-GPR50 increased mitophagosome density marked by LC3-GFP^+^mitoDsRed^+^ puncta. In contrast, neither GPR50^mLIR^ nor GPR50^T532A, Δ502-505^ mutants affected mitophagosome density (Fig. 4I, J). These results indicate that GPR50 promotes mitophagy in a way dependent on its binding to LC3. Mutations at GPR50^T532A^, GPR50^Δ502-505^, which attenuate the binding of GPR50 to LC3, impair GPR50-mediated mitophagy.

Of note, we observed GPR50 distributed in the LC3-GFP^+^mitoDsRed^+^ mitophagosomes and LC3-GFP^+^ autophagosomes/phagophores (Fig. 4I–L), which was attenuated by both mLIR and ASD mutations. This indicates that GPR50 recruits the assembling autophagosomes to mitochondria via binding to LC3, a feature shared with other mitophagy receptors such as FUNDC1, PHB2 and DISC1[19, 25, 26].

These results indicate that GPR50, a novel mitophagy receptor, is recruited to the depolarized mitochondria, which marks the damaged mitochondria and recruits the assembling autophagosomes via binding to LC3. Via this way, GPR50 and the damaged mitochondria are engulfed by autophagosomes for further degradation. Mutations of GPR50 at either T532A or Δ502-505 attenuate this process.

### Deficiency of GPR50 impairs mitochondrial metabolism and increases ROS levels

**Fig. 5:**
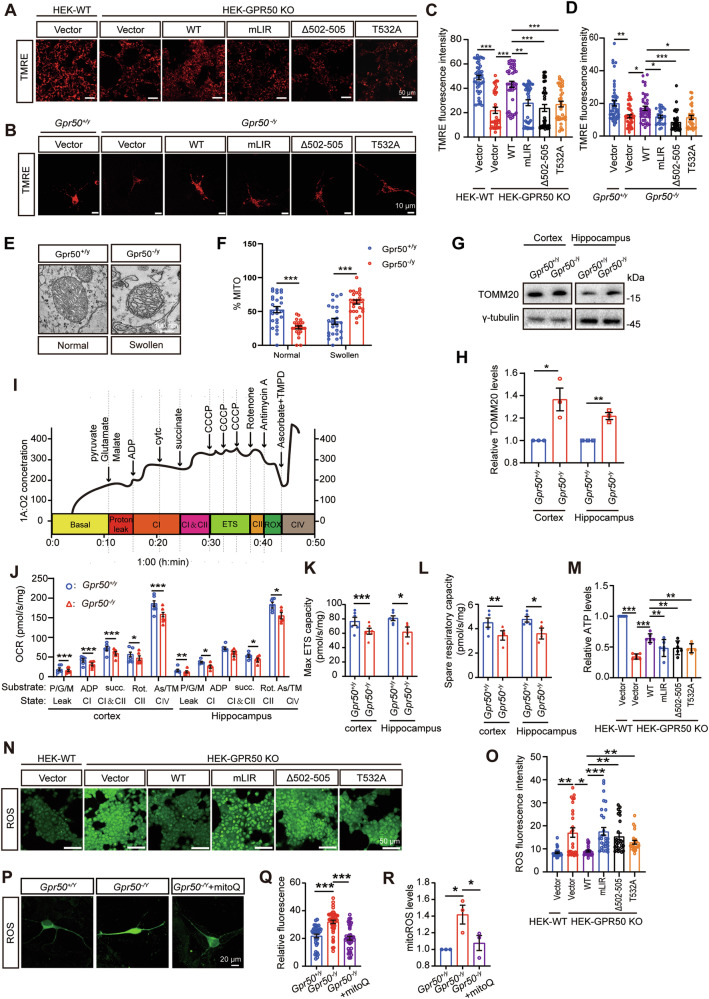
Deficiency of GPR50 impairs mitochondrial metabolism and increases ROS levels. **A**–**D** TMRE analysis of HEK-WT, HEK-GPR50 KO cells and primary cultured *Gpr50*^+/y^, *Gpr50*^−/y^ neurons transfected with GPR50-FLAG WT, mLIR, Δ502-505, or T532A (**A**, **B**). TMRE fluorescence intensity analysis of HEK cells and primary neurons (**C**, **D**). Scale bars: 50 μm and 10 μm respectively. One-way ANOVA for HEK cells (F_5, 233_ = 22.058, *p* = 0.000) followed by Dunnett T3 post hoc tests for WT+Vector versus KO+Vector (*p* = 0.000), KO+Vector versus KO + WT (*p* = 0.000), KO + WT versus KO+mLIR (*p* = 0.001), KO + WT versus KO + Δ502-505 (*p* = 0.000), KO + WT versus KO + T532A (*p* = 0.000). *n* = 37–45 cells per group from 3 independent experiments (**C**). One-way ANOVA for primary neurons (F_5, 220_ = 11.339, *p* = 0.000) followed by Dunnett T3 post hoc tests for WT+Vector versus KO+Vector (*p* = 0.001), KO+Vector versus KO + WT (*p* = 0.042), KO + WT versus KO+mLIR (*p* = 0.028), KO + WT versus KO + Δ502-505 (*p* = 0.000), KO + WT versus KO + T532A (*p* = 0.039). *n* = 26–48 cells per group from 3 independent experiments (**D**). **E**, **F** TEM analysis of mitochondria in neurons of hippocampal CA1 region of Gpr50^+/y^ and Gpr50^−/y^ mice. Representative images (**E**). Scale bars: 100 nm. Percentage of normal and mitochondria with swollen cristae (**F**). One-way ANOVA (F_5, 150_ = 44.071, *p* = 0.000) followed by LSD post hoc tests for *Gpr50*^+/y^ normal versus *Gpr50*^−/y^ normal (*p* = 0.000), *Gpr50*^+/y^ swollen versus *Gpr50*^−/y^ swollen (*p* = 0.000). *n* = 20–26 microscope fields from 3 mice per genotype (**F**). **G**, **H** Western blotting analysis of TOMM20 levels in the cortex and hippocampus of *Gpr50*^+/y^ and *Gpr50*^−/y^ mice (**G**). Relative TOMM20 levels (**H**). Student’s t-test for cortex (t_4_ = −3.626, *p* = 0.022) and hippocampus (t_4_ = −7.007, *p* = 0.002). *n* = 3 mice per genotype (**H**). **I**–**L** A representative graph of the high-resolution respirometry in adult *Gpr50*^+/y^ and *Gpr50*^−/y^ brain. The black line represents the oxygen consumption flux (**I**). Quantification of the oxygen consumption in the hippocampus and cortex of adult *Gpr50*^+/y^ and *Gpr50*^−/y^ mice (**J**). The max ETS capacity (**K**) and spare respiratory capacity (**L**). Student’s t-test for cortex P/G/M (t_5_ = 10.684, *p* = 0.000), ADP (t_5_ = 8.351, *p* = 0.000), succ (t_5_ = 17.792, *p* = 0.000), Rot (t_5_ = 2.937, *p* = 0.032), As/TM (t_5_ = 11.430, *p* = 0.000) (**H**). Student’s t-test for hippocampus P/G/M (t_5_ = 4.733, *p* = 0.005), ADP (t_5_ = 3.774, *p* = 0.013), succ (t_5_ = 2.471, *p* = 0.056), Rot (t_5_ = 3.482, *p* = 0.018), As/TM (t_5_ = 6.329, *p* = 0.001). *n* = 6 mice per genotype (**J**). Student’s t-test for Max ETS capacity in cortex (t_5_ = 9.659, *p* = 0.000) and hippocampus (t_5_ = 3.461, *p* = 0.018). n = 6 mice per genotype (**K**). Student’s t-test for Spare respiratory capacity in cortex (t_8_ = 4.910, *p* = 0.001) and hippocampus (t_4_ = 3.357, *p* = 0.028). *n* = 5 mice per genotype (**L**). **M** ATP analysis in HEK-WT and HEK-GPR50 KO cells transfected with GPR50-FLAG WT, mLIR, Δ502-505, or T532A. Relative ATP levels. One-way ANOVA (F_5, 30_ = 43.660, *p* = 0.000) followed by LSD post hoc tests for WT+Vector versus KO+Vector (*p* = 0.000), KO+Vector versus KO + WT (*p* = 0.000), KO + WT versus KO + mLIR (*p* = 0.003), KO + WT versus KO + Δ502-505 (*p* = 0.003), KO + WT versus KO + T532A (*p* = 0.002). *n* = 6 independent experiments (**M**). **N**, **O** HEK-WT and HEK-GPR50 KO cells were transfected with GPR50-FLAG WT, mLIR, Δ502-505, or T532A and subjected to ROS analysis (**N**). ROS fluorescence intensity was quantified (**O**). One-way ANOVA (F_5, 155_ = 9.657, *p* = 0.000) followed by Dunnett T3 post hoc tests for WT+Vector versus KO+Vector (*p* = 0.004), KO + Vector versus KO + WT (*p* = 0.010), KO + WT versus KO+mLIR (*p* = 0.000), KO + WT versus KO + Δ502-505 (*p* = 0.003), KO + WT versus KO + T532A (*p* = 0.002). *n* = 25–31 cells per group from 3 independent experiments (**O**). **P**–**R** ROS analysis in *Gpr50*^+/y^, *Gpr50*^−/y^, and MitoQ-treated *Gpr50*^−/y^ primary neurons (**P**). Scale bars: 20 μm. Relative fluorescence was quantified (**Q**). ROS analysis in the hippocampal slices of *Gpr50*^+/y^, *Gpr50*^−/y^, and MitoQ-treated *Gpr50*^−/y^ mice (**R**). One-way ANOVA (F_2, 117_ = 25.695, *p* = 0.000) followed by LSD post hoc tests for *Gpr50*^+/y^ versus *Gpr50*^−/y^ (*p* = 0.000), *Gpr50*^+/y^ versus *Gpr50*^−/y^ +MitoQ (*p* = 0.000). *n* = 45–60 cells from 3 mice per group (**Q**). One-way ANOVA (F_2, 6_ = 7.053, *p* = 0.027) followed by LSD post hoc tests for *Gpr50*^+/y^ versus *Gpr50*^−/y^ (*p* = 0.012), *Gpr50*^−/y^ versus *Gpr50*^−/y^ +MitoQ (*p* = 0.028). *n* = 30–40 slices from 3 mice per group (**R**). Data are presented as mean ± SEM.*p < 0.05; **p < 0.01; ***p < 0.001. n.s, non-significance.

Immunofluorescence staining on the mouse cortical and hippocampal sections and the primary cultured neurons revealed that neurons predominantly express GPR50. Few GPR50 was detected in astrocytes (Supplementary Fig. 1C–F). The specificity of the GPR50 antibody was validated by the absence of GPR50 immunoreactivity in *Gpr50*^*−/y*^ brains and HEK-GPR50 KO cells (Supplementary Fig. 1G, H; Supplementary Fig. 5). As GPR50 plays an essential role in mitophagy, we further analyzed whether a GPR50 deficiency would cause mitochondrial dysfunction. Both GPR50-deficient HEK293T cells (Fig. 5A, C) and mouse primary cortical neurons (Fig. 5B, D) exhibited a striking decrease in the fluorescent density of TMRE, an established marker for mitochondrial membrane potential [27], indicating GPR50 deficiency results in the accumulation of depolarized mitochondria in these cells. These phenomena were rescued by transfection of WT GPR50 plasmid but not by transfection of GPR50 mLIR or GPR50 ASD mutants (GPR50^T532A^, GPR50^Δ502-505^) (Fig. 5A–D), indicating GPR50 maintains mitochondrial quality in a mechanism dependent on its binding to LC3. TEM analysis unveiled that the mitochondria in *Gpr50*^*−/y*^ neurons exhibited notable vacuolization and swelling within the cristae (Fig. 5E, F). The mitochondrial protein TOMM20 was accumulated in the brains of adult *Gpr50*^*−/y*^ mice (Fig. 5G, H). These results indicate that GPR50 deficiency results in the accumulation of the damaged mitochondria.

We further examined whether a deficiency of GPR50 affects mitochondrial metabolism, which is closely linked to neuronal development [2]. The tissue homogenates from adult *Gpr*50^−/y^ and *Gpr50*^*+/y*^ hippocampus and cortex were subjected to high-resolution respirometry to examine the mitochondrial respiratory capacity (Fig. 5I) and compared to *Gpr50*^*+/y*^ mice, adult *Gpr50*^*−/y*^ mice exhibited a significant decrease in oxygen consumption rate (OCR) through complexes I, II, and IV (Fig. 5J), the max electron transport system (ETS) capacity, and spare respiratory capability in the hippocampus and cortex (Fig. 5K, L). The proton leak, closely associated with ATP and ROS production [28], was also decreased in the hippocampus and cortex of *Gpr50*^*−/y*^ mice (Fig. 5J). These results indicate that a deficiency of GPR50 impairs mitochondrial OXPHOS. Consistent with these observations, GPR50-deficient HEK293T cells exhibited decreased ATP production (Fig. 5M) and increased ROS levels (Fig. 5N, O), which was rescued by transfection of WT GPR50, but not GPR50^mLIR^ or GPR50^T532A, Δ502-505^ mutant, indicating the abnormal mitochondrial metabolism were ascribed to a defective GPR50-mediated mitophagy. The elevated mitochondrial reactive oxygen species (ROS) were also detected in the *Gpr50*^*−/y*^ brain slices (Fig. 5R) and primary cultured neurons derived from *Gpr50*^*−/y*^ mice (Fig. 5P, Q), that was blocked by treatment with mitoQ, a mitochondrially targeted antioxidant [29]. Thus, these results indicate that defective GPR50-mediated mitophagy impairs mitochondrial OXPHOS, resulting in insufficient ATP production and overproduction of ROS.

### GPR50 is required in neuronal development, which is attenuated by both ASD mutations

**Fig. 6:**
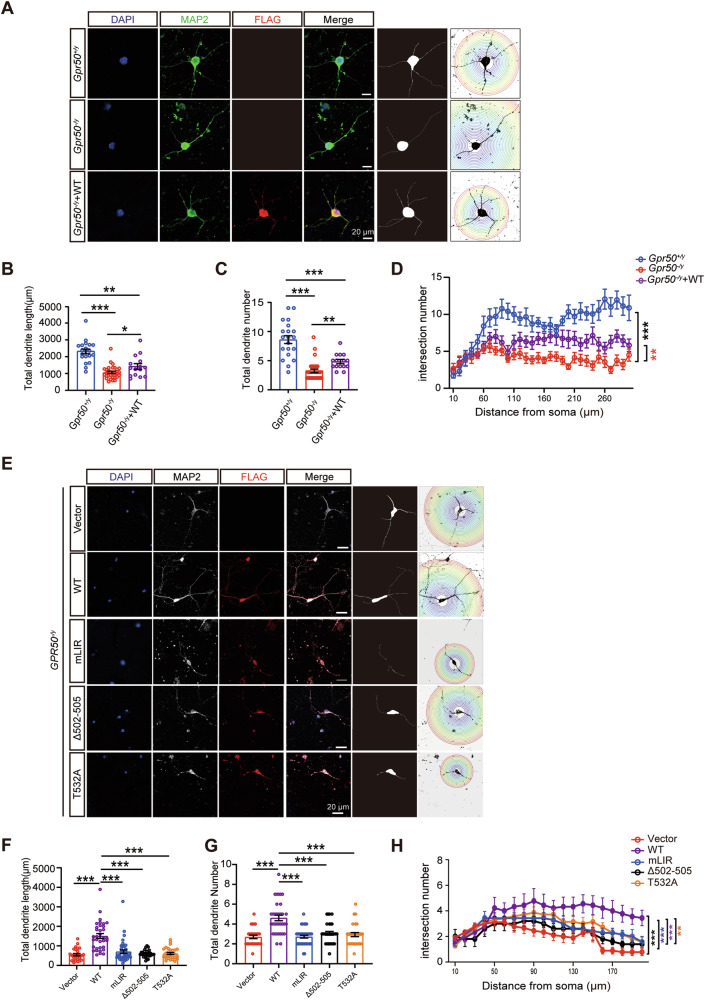
GPR50 is required in neuronal development, which is attenuated by ASD mutations. **A**–**D** The primary cultured neurons derived from newborn *Gpr50*^+/y^ and *Gpr50*^−/y^ mice were transfected with GPR50-FLAG WT or empty vector and immunostained for MAP2, FLAG, and DAPI (**A**). Length (**B**) and numbers (**C**) of neuronal dendrites. Sholl analysis of distance from neuronal soma (**D**). Scale bars: 20 μm. One-way ANOVA (F_2, 62_ = 16.166, *p* = 0.000) followed by LSD post hoc tests for *Gpr50*^+/y^ versus *Gpr50*^−/y^ (*p* = 0.000), *Gpr50*^+/y^ versus *Gpr50*^−/y^ + WT (*p* = 0.009), *Gpr50*^−/y^ versus *Gpr50*^−/y^ + WT (*p* = 0.026) (**B**). One-way ANOVA (F_2, 62_ = 37.545, *p* = 0.000) followed by Dunnett T3 post hoc tests for *Gpr50*^+/y^ versus *Gpr50*^−/y^ (*p* = 0.000), *Gpr50*^+/y^ versus *Gpr50*^−/y^ + WT (*p* = 0.000), *Gpr50*^−/y^ versus *Gpr50*^−/y^ + WT (*p* = 0.004) (**C**). Two-way ANOVA (F_2, 1409_ = 12.443, *p* = 0.000) followed by LSD post hoc tests for *Gpr50*^+/y^ versus *Gpr50*^−/y^ (*p* = 0.000), *Gpr50*^−/y^ versus *Gpr50*^−/y^ + WT (*p* = 0.001) (**D**). *n* = 15–30 cells per group from 3 independent experiments (**A**–**D**). **H** The primary cultured neurons derived from newborn *Gpr50*^−/y^ mice were transfected with GPR50-FLAG WT, mLIR, Δ502-505 or T532A as rescue group and immunostained for MAP2, FLAG, and DAPI (**E**). Length (**F**) and numbers (**G**) of neuronal dendrites. Sholl analysis of distance from neuronal soma (**H**). Scale bars: 20 μm. One-way ANOVA (F_3, 117_ = 35.448, *p* = 0.000) followed by Dunnett T3 post hoc tests for Vector versus WT (*p* = 0.000), WT versus mLIR (*p* = 0.000), WT versus Δ502-505 (*p* = 0.000), WT versus T532A (*p* = 0.000) (**F**). One-way ANOVA (F_3, 116_ = 16.139, *p* = 0.000) followed by Dunnett T3 post hoc tests for Vector versus WT (*p* = 0.000), WT versus mLIR (*p* = 0.000), WT versus Δ502-505 (*p* = 0.000), WT versus T532A (*p* = 0.000) (**G**). Two-way ANOVA (F_3, 597_ = 44.593, *p* = 0.000) followed by Dunnett T3 post hoc tests for Vector versus WT (*p* = 0.000), WT versus mLIR (*p* = 0.000), WT versus Δ502-505 (*p* = 0.000), WT versus T532A (p = 0.004) (**H**). *n* = 27-34 cells per group from 3 independent experiments (**E**–**H**). Data are presented as mean ± SEM. *p < 0.05; **p < 0.01; ***p < 0.001. n.s, non-significance.

We then examined whether GPR50 is required in neuronal development. The neurons derived from newborn *Gpr50*^*+/y*^ and *Gpr50*^*−/y*^ mouse pups were cultured for 5–7 days and immunostained for MAP2 (Fig. 6A, E). Compared to *Gpr50*^*+/y*^ neurons, *Gpr50*^−/y^ neurons displayed smaller complexity of neuronal dendrites as evidenced by the shorter neuronal dendrites (Fig. 6A, B), fewer dendritic branches (Fig. 6A, C, D), that was rescued partially by transfection of WT GPR50 plasmid. The failure of complete rescue may be due to the delayed transfection performed after three days of culturing. These results indicate that GPR50 plays an essential role in neuronal development. In contrast to WT GPR50, neither ASD mutants (T532A and Δ502-50^5^) nor mLIR mutants exhibited a rescue effect in *Gpr50*^−/y^ neurons (Fig. 6E–H), indicating that GPR50-mediated mitophagy is required in neuronal development.

### Deficiency of GPR50 results in defective social behaviors in mice

**Fig. 7:**
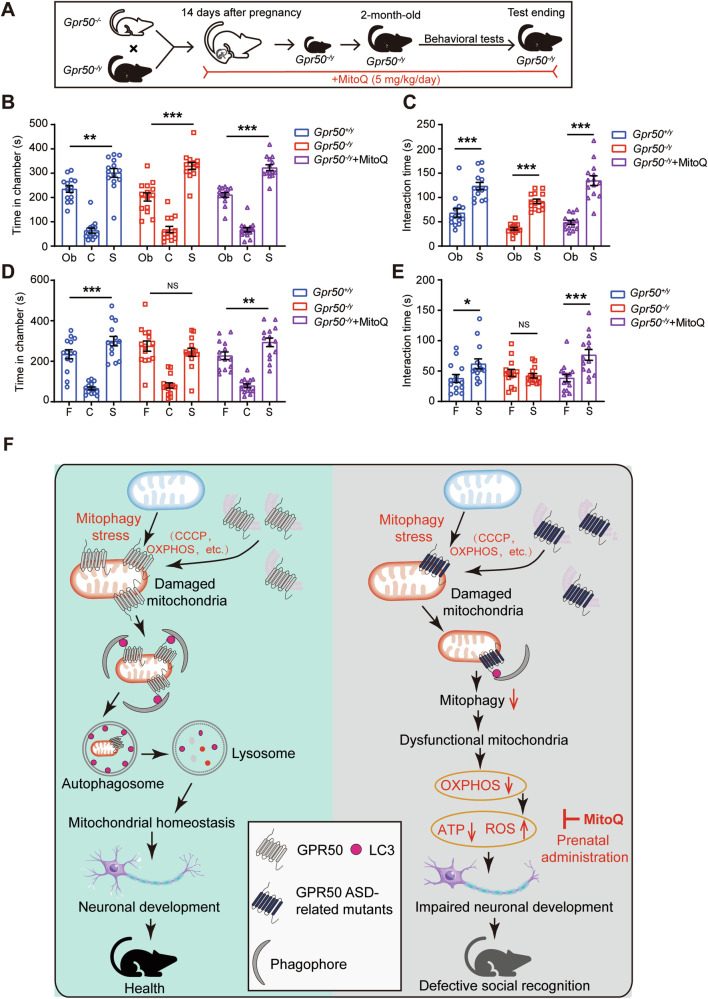
The administration of MitoQ prenatally rescues defective social recognition of GPR50-deficient mice. **A** Schematic description of experimental procedures. *Gpr50*^−/y^ mice were administrated with MitoQ (5 mg/kg/day) starting from E14. MitoQ treatment continued after the pubs were weaned until behavioral tests. The control mice were fed a standard diet. **B**–**E** Three chamber tests in *Gpr50*^+/y^ and *Gpr50*^−/y^ mice. *Gpr50*^−/y^ mice were treated with MitoQ as a rescue group. Time the mice spent in chambers containing either Object (Ob in **B**) or Familiar mice (F in **D**) versus Stranger (S in **B**, **D**) mice. Time the mice spent interacting with either Object (Ob in **C**) or Familiar mice (F in **E**) versus Stranger (S in **C**, **E**) mice. One-way ANOVA (F_8, 117_ = 68.037, *p* = 0.000) followed by LSD post hoc tests for *Gpr50*^+/y^ Object versus *Gpr50*^+/y^ Stranger (*p* = 0.001), *Gpr50*^−/y^ Object versus *Gpr50*^−/y^ Stranger (*p* = 0.000), *Gpr50*^−/y^ +MitoQ Object versus *Gpr50*^−/y^ +MitoQ Stranger (*p* = 0.000) (**B**). One-way ANOVA (F_8, 123_ = 70.486, *p* = 0.000) followed by LSD post hoc tests for *Gpr50*^+/y^ Familiar versus *Gpr50*^+/y^ Stranger (p = 0.001), *Gpr50*^−/y^ Familiar versus *Gpr50*^−/y^ Stranger (*p* = 0.226), *Gpr50*^−/y^ +MitoQ Familiar versus *Gpr50*^−/y^+MitoQ Stranger (*p* = 0.000) (**D**). One-way ANOVA (F_5, 78_ = 32.580, *p* = 0.000) followed by LSD post hoc tests for *Gpr50*^−/y^ Object versus *Gpr50*^−/y^ Stranger (*p* = 0.000), *Gpr50*^−/y^ Object versus *Gpr50*^−/y^ Stranger (*p* = 0.000), *Gpr50*^−/y^+MitoQ Object versus *Gpr50*^−/y^+MitoQ Stranger (*p* = 0.000) (**C**). One-way ANOVA (F_5, 78_ = 5.273, *p* = 0.000) followed by LSD post hoc tests for *Gpr50*^+/y^ Familiar versus *Gpr50*^+/y^ Stranger (p = 0.014), *Gpr50*^−/y^ Familiar versus *Gpr50*^−/y^ Stranger (*p* = 0.610), *Gpr50*^−/y^+MitoQ Familiar versus *Gpr50*^−/y^+MitoQ Stranger (*p* = 0.000) (**E**). *n* = 14–15 mice per genotype (**B**–**E**). **F** Schematic summary of the role of GPR50-mediated mitophagy in neuronal development. GPR50 is a novel mitophagy receptor. Upon mitophagy stress, such as the depolarization of mitochondrial membrane induced by either CCCP or high OXPHOS, GPR50 is recruited to the damaged mitochondria, where it interacts with other mitophagy regulators and facilitates the recruitment of the assembling autophagosomes, promoting mitophagy. In this way, GPR50 maintains mitochondrial metabolism in the developing neurons. Mutations at either Δ502-505 or T532A, disrupts the binding capability of GPR50 to LC3 and the recruitment of GPR50 to the damaged mitochondria, thus attenuating GPR50-mediated mitophagy, resulting in insufficient ATP production and overproduction of ROS, leading to defective social recognition behaviors of the mouse. The administration of mitoQ prenatally rescues the defective social recognition behaviors of GPR50-deficient mice. Data are presented as mean ± SEM.*p < 0.05; **p < 0.01; ***p < 0.001. n.s, non-significance.

Adult *Gpr50*^*−/y*^ mice exhibited normal fertility but a smaller body size than *Gpr50*^*+/y*^ mice (Supplementary Fig. 2M). Both *Gpr50*^*−/y*^ and *Gpr50*^*+/y*^ mice can survive into adulthood. Considering the genetic link of GPR50 to ASD, depression, and bipolar disorders [11, 12], all of which display impaired social interaction, we examined whether a deficiency of GPR50 is linked to defective social behaviors by subjecting the mice to a three-chamber test (Supplementary Fig. 3A). In the first phase of the three-chamber test, both adult *Gpr50*^*+/y*^ and *Gpr50*^*−/y*^ mice exhibited a preference for the stranger mice as evidenced by their spending a more extended time staying in the chamber containing the stranger mice (S) versus the one containing the empty cage (object) (Fig. 7B; Supplementary Fig. 3B) and in interacting with the stranger mice (S) versus with the empty cage (object) (Fig. 7C; Supplementary Fig. 3C), suggesting normal sociability of *Gpr50*^*−/y*^ mouse. In the second phase of three-chamber tests, the test mice were subjected to the chambers containing the stranger mice (S) and the familiar mice (F) on either side, respectively. In contrast to the adult *Gpr50*^*+/y*^ mice, which still exhibited a preference for the stranger mice (S) versus the familiar mice (F), *Gpr50*^*−/y*^ mice lost such preference as reflected by they spent comparable time in staying in the chamber containing the stranger mice (S) versus in the one containing the familiar mice (F) (Fig. 7D; Supplementary Fig. 3D), and in interacting with the stranger mice (S) versus the familiar mice (F) (Fig. 7E; Supplementary Fig. 3E). *Gpr50*^*−/y*^ and *Gpr50*^*+/y*^ mice displayed similar olfactory function in the buried food test, excluding the possibility that the social discrepancy was ascribed to the olfactory dysfunction of *Gpr50*^−/y^ mice (Supplementary Fig. 3F). Thus, a deficiency of GPR50 results in impaired social recognition and memory in mice.

### The administration of MitoQ prenatally rescues autism-like behavior in GPR50-deficient mice

ROS is predominantly produced by mitochondria and is a byproduct of OXPHOS [30]. ROS acts as a signaling factor at low levels and is required in neuronal development, such as neuronal polarity, growth cone pathfinding, neurite outgrowth, and synaptic plasticity [31]. In contrast, excessive ROS accumulation impairs neuronal development and synaptic plasticity, leading to neurodegeneration [31]. Intriguingly, excessive ROS forms a vicious cycle with defective mitophagy. Excessive ROS activates mitophagy to accelerate mitochondrial turnover. Defective mitophagy, in turn, results in excessive ROS production. We wondered whether the elimination of the overproduction of ROS would terminate the vicious cycle between defective mitophagy and ROS and bring beneficial effects on GPR50-deficient mice. MitoQ, a mitochondrially targeted antioxidant, effectively blocks ROS and prevents mitochondrial oxidative damage [29]. Thus, we administered orally *Gpr50*^*−/y*^ mice with mitoQ starting at E14, an active developmental stage for neurons, by feeding their pregnant mother mice with mitoQ until they were weaned. Then, *Gpr50*^*−/y*^ pubs were administered orally with MitoQ until they were subjected to behavioral tests (Fig. 7A). The results showed that administration of mitoQ prenatally rescued the impaired social recognition of *Gpr50*^*−/y*^ mouse as evidenced by that mitoQ-treated *Gpr50*^*−/y*^ mice regained the preference for the stranger mouse versus the familiar mouse in the three-chamber social interaction test (Fig. 7B–E). Thus, the impaired social recognition caused by the defective GPR50-mediated mitophagy is prevented by eliminating excessive ROS at the early developing stage.

## Discussion

We herein identify that GPR50 is a novel mitophagy receptor, which is required in mitophagy stress-induced mitophagy. GPR50 is recruited to the mitochondria upon mitophagy stress, where it marks the damaged mitochondria and initiates the recruitment of the assembling autophagosomes (phagophores), facilitating the damaged mitochondria to be engulfed by autophagosomes. In this way, GPR50 maintains mitochondrial OXPHOS in the developing neurons. Deficiency of GPR50 impairs mitochondrial OXPHOS, which results in an insufficient production of ATP and an overproduction of ROS, eventually leading to delayed neuronal development and defective social recognition. The prenatal administration of mitoQ, a mitochondrial antioxidant, rescues the defective social behaviors of GPR50-deficient mice. Both Δ502-505 and T532A, two GPR50 variations genetically associated with ASD, depression, and bipolar disorders, attenuate the binding of GPR50 to LC3 and the mitochondrial recruitment of GPR50, impairing GPR50-mediated mitophagy and neuronal development (Fig. 7F). This study provides a novel mechanism underlying how developing neurons utilize mitophagy to maintain mitochondrial OXPHOS.

One of the novelties of this study is that we identify GPR50 as a novel mitophagy receptor. So far, only a limited number of mitophagy receptors have been identified, including BNIP3 [32], NIX [33], FUNDC1 [34], BCL2L13 [35], FKBP8 [36, 37], AMBRA1 [38, 39], MCL-1 [40], SAMM50 [41], PHB2 [26], Cardiolipin [42, 43] and DISC1 [19, 25]. We identify that GPR50 harbors a LIR motif, e.g., Y^305^WTI^308^, which binds directly to LC3. Mutating the LIR motif abolished its binding to recombinant LC3 in the ELISA binding assay. In contrast, mutating the LIR motif only attenuates its binding to LC3 in CoIP and PLA assay. The discrepancy may be because GPR50 forms a protein complex with other LIR-containing mitophagy receptors such as FUNDC1 [25], PHB2 [26], and FKBP8 [36]. However, it is also possible that GPR50 harbors other LIR motifs, which remain to be further identified. The impairment in the binding of GPR50 to LC3 by either mutating the LIR motif or ASD variation attenuates GPR50-mediated mitophagy, concurrent with a decreased engulfment of GPR50 by mitophagosomes. These results indicate that the mitochondrial GPR50, like other mitophagy receptors, recruits the phagophores adjacent to the damaged mitochondrial portion. With the growth of phagophores, the damaged mitochondrial portion, together with GPR50, is engulfed by the autophagosomes. In contrast to the most known mitophagy receptors, which distribute endogenously in mitochondria in normal conditions [44], quite a few GPR50 localizes in the mitochondria in normal conditions. GPR50 is recruited to the mitochondria upon induction of mitophagy stress, e.g., CCCP treatment and high OXPHOS, which decreases mitochondrial membrane potential [3]. This provides a more flexible way to initiate mitophagy in response to mitophagy stress. Upon mitophagy stress, the accumulation of depolarized mitochondria initiates the recruitment of GPR50. The mitochondrial GPR50 marks the mitochondrial portion and recruits the assembling autophagosomes, enhancing mitophagy efficacy. Thus, we propose that GPR50-mediated mitophagy facilitates mitochondrial turnover in response to mitophagy stress.

Through a BiCAP assay, we observe an extensive interaction of GPR50 with other mitophagy regulators. Among them, FUNDC1 [25], PHB2 [26], and FKBP8 [36] contain LIR and serve as mitophagy receptors. PGAM5, HUWE1, Casein kinase 2, MARK2, and PARK7 regulate mitophagy via a posttranslational mechanism [42–49]. For example, PGAM5, an atypical protein phosphatase, promotes mitophagy via phosphosrylating FUNDC1 and stabilizing PINK1 [45, 46]. HUWE1, an E3 ubiquitin ligase, is required in AMBRA1-mediated mitophagy [47, 48]. PARK7, downstream of PINK1 and PARKIN, is translocated to the depolarized mitochondria and binds with OPTN (optineurin) [49, 50]. RHOT1 and RHOT2 are small GTPases with calcium-sensing EF-hands that function as adaptors between the mitochondrion and its molecular motors. RHOT1 and RHOT2 are not only substrates for PINK1-PARK-dependent degradation, but they also play an active role in the process of mitophagy. They function as a calcium-sensing docking site for PARKIN and are required for mitochondrial damage-induced PARKIN translocation to mitochondria and subsequent mitophagy [51]. FIS1 and OPA are mitochondrial remodeling proteins involved in mitochondrial fission and fragmentation [52–54]. Other factors such as VDAC1 [55], SLC25A5 [56], CERS1 [57], and VCP [58] are also robust mitophagy regulators. These results suggest that GPR50 coordinates with other mitophagy regulators to activate mitophagy. Thus, by recruiting GPR50 to the damaged mitochondria, a protein complex forms to facilitate mitophagy, which enhances mitochondrial turnover in response to mitophagy stress.

We have observed that the mitochondrial recruitment of GPR50 does not rely on its binding to LC3. Intriguingly, both Δ502-505 and T532A GPR50 variations, which attenuate the binding of GPR50 to LC3, impair the mitophagy stress-induced mitochondrial recruitment of GPR50, suggesting that other mechanisms are involved. The damaged mitochondria initiate a series of signal cascades on their surface, such as PINK1-mediated phosphorylation and PARKIN-mediated ubiquitination, which leads to the recruitment of autophagy machinery to the mitochondria with the aid of mitophagy receptors [3]. In this context, among the binding partners of GPR50 identified by BiCAP, OPA, VCP, and PARK7 are also translocated to the depolarized mitochondria. RHOT1 and RHOT2 are required for mitochondrial damage-induced PARKIN translocation to mitochondria [51]. We surmise a similar mechanism underlying the mitochondrial recruitment of GPR50, which requires further investigation.

High mitophagy stress exists during OXPHOS [59]. Mitophagy is broadly activated during OXPHOS to enhance mitochondrial turnover in response to the high demand for energy production [3], which is consistent with our observation that defective GPR50-mediated mitophagy impairs mitochondrial OXPHOS, resulting in insufficient ATP production. A deficiency of GPR50-mediated mitophagy also causes elevated ROS levels as the mitochondrial electron transfer chain (ETC) is the primary side to produce ROS [60]. We are not surprised to observe that administration of mitoQ at an earlier embryonic stage rescues impaired social memory and recognition of *Gpr50*^*−/y*^ mice. In physiological conditions, low levels of ROS play essential roles in multiple processes of neuronal development, such as neuronal polarization, neurite outgrowth, and synaptic plasticity [31]. In contrast, excessive ROS impairs neuronal function [61]. Mitochondrial ROS is produced as a byproduct during mitochondrial OXPHOS because of the electron leakage of the ETC [30]. Excessive ROS depolarizes mitochondrial membranes and activates mitophagy to eliminate the ROS-producing mitochondria during OXPHOS. This prevents ROS accumulation [45, 62, 63]. Defective GPR50-mediated mitophagy impairs this process, causing excessive ROS accumulation. The latter further impairs mitochondria and exaggerates mitophagy stress, thus forming a vicious cycle. We surmise that amelioration of ROS production disrupts this vicious cycle by attenuating mitophagy stress to a normal condition, where GPR50-mediated mitophagy is not required. This explains why the administration of mitoQ at an earlier embryonic stage is sufficient to rescue the impaired social memory and recognition of *Gpr50*^*−/y*^ mice. Considering that elevated ROS levels are broadly detected in the brains of ASD patients [8, 9, 64], we herein offer a potential therapeutic strategy in the earlier intervention of this neurodevelopmental disorder.

## Materials and methods

### Plasmids and siRNA

GFP-LC3 was described previously [65]. MitoDsRed was described [19]. The cDNA encoding WT GPR50 (human) or mutant GPR50 was inserted into the FLAG sequence at the C terminal and cloned into the pcDNA3.1 (+) vector between the BamHI and NotI sites. The mutation information of GPR50 mutants is as follows: GPR50^T532A^, a.a. T^532^ were mutated into A^532^, GPR50^Δ502-505^, a.a. 502-505 were deleted. All constructs were confirmed by sequencing. The sequence of siRNA was as follows: GPR50 siRNA (5’-GGAUCUUCAGUGUGCGCAATT-3’), scrambled siRNA (5’-UUCUCCGAACGUGUCACGUTT-3’) (Genepharma).

### Mice

*Gpr50*^*−/−*^ mice were generated by replacing the second exon with the Lacz-neo cassette and maintained on a C57BL/6 N background by crossing heterozygous transgenic mice with C57BL/6 N breeders. *Gpr50*^*−/y*^ mice were obtained by hybridization between *Gpr50*^*−/−*^ mice and *Gpr50*^*+/y*^ mice. For PCR genotyping, the following primers were used (F1: 5’-ATCCGGGGGTACCGCGTCGAG-3’; R1: 5’-TACCTCCACCTCCTCCAGCAT-3’; F2: 5’-CAGAGTCACCTGGGACTTGCT-3’; R2: 5’-CACAACGGGTTCTTCTGTTAGTCC-3’; F3: 5’-CAGAGTCACCTGGGACTTGCT-3; R3: 5’-GTAGCAGTAACGGTTGATGGCAATG-3’). A product size of 390 bp was obtained for the WT mouse. The product sizes of 353 bp and 357 bp were identified for GPR50 knockout mice together. All mice were group-housed with 3-5 same-sex cage mates in standard mouse cages in a pathogen-free barrier facility on a 12-h light-dark cycle with lights on at 07:00 and a controlled temperature range of 22–25 °C. Food and water were provided ad lobitum. Behavioral tests were performed during the light phase. All experimental procedures were preapproved by the Ethics Committee of Soochow University and conformed to the Institutional Animal Care and Use Committee guidelines of Soochow University (reference number: 202211A0519).

### Generation of GPR50 CRISPR-Cas9 KO cell lines

For the generation of GPR50 KO HEK293T cells, single guide RNAs (sgRNAs) were designed using the online CRISPR design tool (Red CottonTM, Guangzhou, China, https://en.rc-crispr.com/). The exon region of GPR50 was selected to be targeted by CRISPR/Cas9 genome editing. A ranked list of sgRNAs was generated with specificity and efficiency scores. The pairs of oligos for two targeting sites were annealed and ligated to the YKO-RP006 vector (Ubigene Bioscience Co. Ltd., Guangzhou, China). The YKO-RP006-hRABL6 [gRNA] plasmids containing each target sgRNA sequence were transfected into cells with Lipofectamine 3000 (Thermo Fisher Scientific). 24–48 h after the transfection, puromycin was added to screen the cells. After antibiotic selection, cells were diluted using the limited dilution method and inoculated into a 96-well plate. Single GPR50 KO clones were performed after being cultured for 2–4 weeks and validated by PCR, western blotting, and Sanger sequencing.

### Cell culture and transfection

HEK293T (female), HEK293, HeLa (female), and MEF cells were obtained from the American Type Culture Collection (ATCC). Cells were maintained at 37 °C with 5% CO_2_ in DMEM-GlutaMAX (Hyclone, SH30243.01) medium supplemented with 10% FBS (Gibco, 005) and 100 U ml^−^^1^ penicillin/streptomycin (Gibco, 15140163). Primary neurons were isolated from the hippocampus and cortex of newborn mouse pubs. The hippocampus and cortex were dissected from the whole brain in ice-cold PBS. The tissues were digested with 0.25% trypsin for 15 min at 37 °C, followed by gentle mechanical trituration. Neurons were cultured in neurobasal-A (Gibco, 10888022) medium supplemented with 2% B27 (Gibco, 17504044), 1% Glumax (Gibco, 35050061) and 1% Penicillin-Streptomycin (P/S) at 37 °C. Primary cultured neurons were transfected with WT GPR50 and GPR50 ASD mutant plasmids using Lipofectamine 2000 (Thermo Fisher Scientific).

### Drug treatment

The *gpr50*^*−/y*^ and *gpr50*^*-/+*^ mice were allowed to mate at 5 pm. The vaginal plugs were checked at 8 am the next day. The mice with vaginal plugs were supposed to be in pregnancy for 0.5 days. The *gpr50*^*-/+*^ mice that were pregnant for 14 days were administrated with mitoQ (New Zealand, 70443110246) orally at 5 mg/kg/d, which were mixed into the food chaw. The pubs were genotyped after birth, and *gpr50*^*−/y*^ pups were fed food containing mitoQ until they were subjected to behavioral tests at 2 months old. *Gpr50*^*+/y*^ and *gpr50*^*−/y*^ mice, fed food with normal saline, served as controls.

Cells were treated with 400 nM bafilomycin A1 (Selleck, S1413) for 12–24 h to block the fusion of autophagosomes to lysosomes. Cells were treated with 50 μM CCCP (Yeasen, China, 40333ES60) for 2 h, 6 h, and 12 h to induce mitochondrial damage.

### Analysis of colocalization of LC3 and MitoDsRed and fragmented mitochondria

The quantification was performed as described previously [19]. HeLa cells co-transfected with LC3-GFP and MitoDsRed plasmid were imaged with a confocal microscope. The colocalization ratio of LC3-GFP and MitoDsRed were analyzed by Image-Pro Plus 6.0 software (Media Cybernetics, Silver Spring, MD) as described. After correcting the background, Pearson’s correlation coefficient was calculated as the colocalization ratio. The mitochondrial fluorescent signals display as network or baculiform shape in normal conditions. We used Image J to measure normal and fragmented mitochondria. Considering the network structure formed through mitochondrial fusion and fission, we assessed a minimum of 15 mitochondria per cell and computed their average length. Mitochondria with an average length equal to or less than 5 μm were categorized as undergoing fragmentation, whereas those exceeding 5 μm were deemed normal. The percentage of cells with fragmented mitochondria was counted [66, 67].

### Proximity ligation assay (PLA)

Cells were transfected with GPR50 WT, GPR50 LIR mutant, and GPR50 ASD mutant plasmids for 30 h. Cells were then fixed with 4% paraformaldehyde for 20 min at RT. Cells were permeabilized in PBS containing 0.3% triton for 5 min, then washed with PBS twice. After being blocked in Duolink block solution for 2 h at RT, cells were incubated with anti-LC3 (Novus, NB100-2220) and mouse anti-FLAG (Sigma-Aldrich, F3165) antibodies which were diluted in Duolink antibody dilution buffer overnight at 4 °C. The following morning, cells were washed for 10 min in washing buffer A, followed by adding the appropriate Duolink secondary antibodies (Sigma-Aldrich, DUO92002, DUO92004) diluted and mixed according to the manufacturer’s instruction. Cells were incubated for 30 min at 37 °C, after which cells were washed with washing buffer A twice, each 5 min. Ligation and amplification steps of the PLA were performed using the Duolink in situ Green Starter kit (Sigma-Aldrich, DUO92014) according to the manufacturer’s instructions. Cells were then mounted in a Prolong Gold mounting medium with DAPI (Invitrogen, P36941). Images were acquired on a Zeiss LSM900 Confocal microscope. PLA spots were counted using Image J software (NIH, Bethesda, MA, USA).

### Quantification of dendritic complexity

Primary neurons were isolated from the hippocampus and cortex of newborn mouse pubs and cultured in neurobasal-A (Gibco, 10888022) medium supplemented with 2% B27 (Gibco, 17504044), 1% Glumax (Gibco, 35050061) and 1% Penicillin-Streptomycin (P/S) at 37 °C. The cultured neurons were transfected with GPR50-FLAG and immunostained for MAP2. The images were captured by an LSM780 confocal microscope (Zeiss, Jena). Sholl analysis was performed using the Image J software under the following parameters (start radius: 2 μm, end radius: 30 μm, radius step: 5 μm). The numbers of intersections at radial intervals of 2 μm starting from the central point of the soma were identified and averaged to create the mean sholl curve.

### Co-immunoprecipitation (Co-IP)

Cultured cells were lysed in RIPA buffer (50 mM Tris-HCl pH 7.5, 150 mM NaCl, 0.5% NP40, 10% glycerol) containing protease inhibitor at 4 °C for 30 min and centrifuged at 16,000 × g for 15 min at 4 °C. The supernatants were collected and subjected to protein quantification using a BCA protein assay kit (Thermo Fisher Scientific, 23225). Cell lysates were incubated with FLAG-conjugated beads (Bimake, B26102) for 12 h at 4 °C. The beads were washed with ice-cold RIPA buffer containing protease inhibitors. Input and co-precipitated fractions were analyzed using SDS–PAGE and immunoblotting. For co-immunoprecipitation of endogenous GPR50 and LC3, brain homogenates were extracted with lysis buffer (50 mM Tris-HCl pH7.4, 150 mM NaCl, 1% NP-40, 0.1% SDS, 10% glycerol) containing protease inhibitor and phosphatase inhibitor. The lysates were incubated with an anti-GPR50 antibody (Proteintech, 21514-1-AP) or anti-LC3 antibody (Novus, NB100-2220) overnight at 4°C and precipitated with Protein A/G agarose beads (Santa Cruz, sc-2003). Supernatants were subjected to SDS–PAGE and immunoblotting.

### BiCAP interactome analysis and sample preparation for MS

HEK 293 T cells co-transfected with the constructs V1–GPR50 WT and V2–GPR50 WT. After 30 h, cells were lysed with RIPA Lysis Buffer (50 mM Tris-HCl pH 7.5, 150 mM NaCl, 0.5 mM EDTA, 12000 rpm1% NP-40, 0.25% sodium deoxycholate, Roche EDTA-free protease inhibitor cocktail and NEM), followed by incubation with GFP-trap beads (Proteintech) overnight at 4 °C on a rotating platform. Briefly, beads were washed and using 8 M urea with 100 mM Tris-Cl (pH 8.5) to denature proteins, then, 10 mM Tris(2-Carboxyethyl)-Phosphine HCl (TCEP, Thermo Scientific) for reduction and 15 mM iodoacetamide (IAA, Sigma) for alkylation were added for reduction and alkylation, respectively. The sample was digested with Trypsin at 1:50 (w/w) (Promega) overnight and stopped by 5% Formic Acid (FA, Thermo Scientific), the peptide mixture was desalted by MonoSpinTM C18 column (GL Science). Desalted mixture was dried with a SpeedVac and resuspened in 0.1% FA for MS analysis.

### HPLC-tandem MS (MS/MS) analysis of peptides

The peptide mixture was analyzed by a home-made 30 cm-long pulled-tip analytical column (75 μm ID packed with ReproSil-Pur C18-AQ 1.9 μm resin, Dr. Maisch GmbH), the column was then placed in-line with an Easy-nLC 1200 nano HPLC (Thermo Scientific) for mass spectrometry analysis. The analytical column temperature was set at 55 °C during the experiments. The mobile phase and elution gradient used for peptide separation were as follows: 0.1% formic acid in water as buffer A and 0.1% formic acid in 80% acetonitrile as buffer B, 0–1 min, 5%–10% B; 1–96 min, 10–40% B; 96–104 min, 40%–60% B, 104–105 min, 60%–100% B, 105–120 min, 100% B. The flow rate was set as 300 nL/min.

Data-dependent MS/MS analysis was performed with a Q Exactive Orbitrap mass spectrometer (Thermo Scientific). Peptides eluted from the LC column were directly electrosprayed into the mass spectrometer with the application of a distal 2.5-kV spray voltage. A cycle of one full-scan MS spectrum (m/z 300-1800) was acquired followed by top 20 MS/MS events, sequentially generated on the first to the twentith most intense ions selected from the full MS spectrum at a 30% normalized collision energy. Full scan resolution was set to 70,000 with automated gain control (AGC) target of 3e6. MS/MS scan resolution was set to 17,500 with isolation window of 1.8 m/z and AGC target of 1e5. The number of microscans was one for both MS and MS/MS scans and the maximum ion injection time was 50 and 100 ms, respectively. The dynamic exclusion settings used were as follows: charge exclusion, 1 and >8; exclude isotopes, on; and exclusion duration, 30 seconds. MS scan functions and LC solvent gradients were controlled by the Xcalibur data system (Thermo Scientific).

### Metabolic extracellular flux analysis

The cellular extracellular acidification rate (ECAR) was determined using the Seahorse XF Glycolytic Rate Assay Kit (Agilent, Santa Clara, 103344-100) on the Seahorse XFe-24 Extracellular Flux Analyzer (Agilent, Santa Clara, USA), following the manufacturer’s protocol. Briefly, cultured cells were plated into a Seahorse XF-24 cell culture microplate (Agilent, 102342-100) overnight. One day before the experiment, the XFe24 sensor cartridge was hydrated overnight at 37 °C in a non-CO_2_ incubator with Seahorse XF calibrant (Agilent, 100840-000). Cells were rinsed and incubated in the Seahorse XF DMEM assay medium (Agilent, 103575-100) supplemented with 1 mM pyruvate sodium, 2 mM glutamine, and 10 mM glucose at 37 °C in a non-CO_2_ incubator for 1 h. Cell culture microplates were then loaded into the Seahorse XFe24 Analyzer. The test of glycolysis rate was performed as follows: baseline, 3 cycles; injection of Rotenone/Antimycin A (final concentration: 0.5 μM), 3 cycles; injection of 2-DG (final concentration: 50 mM), 5 cycles. Each cycle is composed of mix 3 min, wait 2 min, and measure 3 min. After completion of the measurements, the amount of protein within the plate was determined by BCA. Data are presented as pH changes per min in the medium using the Seahorse Wave 2.6.0 version (Agilent), normalized to total protein content. Glycolic Proton Efflux Rate (glycoPER), referring to the proton efflux rate brought by the production of lactic acid during glycolysis, was calculated as the differences between the total PER and the mitochondrial PER. Mitochondrial PER refers to the proton efflux rate from respiratory sources (contribution of mitochondrial/TCA cycle-derived CO_2_ to extracellular acidification). Mitochondrial PER was calculated by measuring OCR before and after adding Rot/AA using the Buffer Factor (BF, 2.8) and CO_2_ contribution factor (CCF).

### Enrichment of mitochondria

HEK293T cells were treated with 50 μM CCCP (Yeasen, China, 40333ES60) for 2, 6, and 12 h and collected. Mitochondria were isolated using the Cell Mitochondria Separation Kit (Beyotime Co., Nantong, China, C3601) as instructed by the manufacturer. Briefly, cells were collected and homogenized on ice in mitochondrial separation reagent A containing 1 mM PMSF (Beyotime Co., Nantong, China, ST506). Cell homogenates were centrifuged at 600 g at 4 °C for 10 min, and then the supernatant was collected and centrifuged at 11,000 g at 4 °C for 10 min. The precipitate was collected and resuspended in mitochondrial buffer A. The supernatant was collected as the cytoplasmic fraction depleted mitochondria.

### High-resolution respirometry

After washing with pre-cooled PBS, the region of the cortex and hippocampus were dissected and put into a tube containing MIR05 respiratory buffer (20 mM HEPES, 110 mM sucrose,10 mM KH_2_PO4, 20 mM taurine, 60 mM lactobionic acid, 3 mM MgCl_2_, 0.5 mM EGTA [pH 7.1], 1 mg/ml fatty acid-free BSA, catalase 280 U/ml). The tissues were homogenized on ice, and the mitochondrial oxygen consumption rate (OCR) was measured via O_2_K (Oroboros instruments). Briefly, the tissue homogenates were placed in the respiration chamber in MIR05. After the baseline recording, the substrate pyruvate (5 mM), glutamate (10 mM), and malate (2 mM) as the substrates of complex I. When the respiration was stabilized, the leak value of complex I was obtained. The maximum oxidative phosphorylation value of complex I was obtained by adding ADP (2.5 mM). Cytochrome C (10 μM) was added to verify the integrity of the mitochondrial membrane. Succinate (10 mM) was added to obtain complexes I and II’s maximum oxidative phosphorylation value. The maximum electron transport capacity was obtained by adding uncoupling agent CCCP (1.5 μM). The maximum electron transport capacity of complex II was obtained by adding rotenone (complex I inhibitor) (0.5 μM). Antimycin A (inhibitor of complex III) (2.5 μM) was added to obtain ROX non-mitochondrial respiration. Add Ascorbate (2 mM) and TMPD (0.5 mM) to detect the respiratory function of complex IV. All measurements were performed at 37 °C.

### Detection of Reactive oxygen (ROS)

Cells transfected with GPR50 WT or GPR50 mutant plasmids were treated for 50 μM CCCP for 2 h. Following the manufacturer’s instructions, the ROS test was determined using a ROS assay kit (Beyotime, S0033S). 10^5^ cells were incubated with CellROX (5 μM) in a cell incubator for 30 mins at 37 °C and washed three times with PBS. Cells were then fixed with 4% PFA for 15 min. The immunofluorescent density was determined under the Zeiss confocal microscope.

The mitochondrial superoxide was detected by using MitoSOX Red (Invitrogen). Brain slices isolated acutely were incubated for 4 h in carboxygenated ACSF at 32 °C. 5 μM MitoSOX Red was added to the brain slices and allowed for incubation for 10 min. Slices were then fixed with ice-cold 4% PFA in PBS overnight at 4 °C and cut into 30 μm sections. Brain slices were mounted onto slides with a mounting medium containing DAPI (Vector Laboratories). Slices were imaged using a Zeiss confocal microscope. All parameters (pinhole, gain, contrast, offset) were held constants for all sections from the same experiment.

### Mitochondrial membrane potential (ΔΨm) assay

The ΔΨm was measured using the Mitochondrial Membrane Potential Assay Kit with TMRM (Beyotime, C2001S) following the manufacturer’s instructions. The TMRE probe was diluted with neurobasal A culture medium and incubated with cultured cells for 30 min. Cells were imaged using a Zeiss fluorescence microscope. All parameters (pinhole, gain, contrast, offset) were held constant for all sections from the same experiment.

### Analysis of ATP levels

ATP levels were analyzed using the ATP detection kit (Beyotime, S0027). The cultured cells were washed with PBS and lysed with 100 μl ATP lysate buffer on ice. The cell lysates were centrifuged at 12,000 g at 4 °C for 5 min, and the supernatants were collected. The ATP detection solution was diluted with ATP detection diluent in a 1:4 ratio. Sample supernatants were mixed with ATP detection solution and standard solution in a 96-well plate following a ratio at 10 μl: 100 μl: 10 μl and analyzed under a luminometer. The ATP levels were calculated based on the standard curve.

### Transmission electron microscopy (TEM)

TEM was conducted in collaboration with the Electron Microscope Facility at Soochow University. Mice were anesthetized with isoflurane and transcardially perfused with ice-cold PBS, followed by a fixing solution comprising 2% paraformaldehyde (PFA) and 3% glutaraldehyde in 0.1 M sodium cacodylate buffer (pH 7.4). Brain specimens were then immersed in this fixing solution at 4 °C overnight. The corpus callosum was dissected into 1 mm³ blocks and further post-fixed overnight in the same solution at 4 °C. Subsequently, the tissues underwent two 15 min washes with 0.1 M phosphoric acid, followed by a 1 h post-fixation step in 1% OsO4 in cacodylate buffer at room temperature. After dehydration with a series of diluted acetone solutions, the specimens were embedded in Lx-112 (Ladd Research Industries). Following polymerization, ultrathin sections ranging from 40 to 60 nm in thickness were prepared and stained with 3% uranyl acetate and lead citrate. Imaging was performed using a Tecnai G2 Spirit BioTwin transmission electron microscope (USA). The normal morphology of mitochondrial inner membranes forms cristae, which increase membrane surface area to accommodate more biochemical reactions. Consequently, mitochondrial cristae are often arranged in uniform and orderly lamellar structures. In summary, we assessed the width of at least 10 cristae per mitochondrion and calculated their average width. Mitochondria with an average width equal to or less than 20 nm were classified as normal, while those exceeding 20 nm were classified as swollen. Mitochondrial morphology within neurons in the hippocampal CA1 region was analyzed from three mice per group using Image J software (NIH, Bethesda, MA, USA).

### Behavioral tests

2-month-old *Gpr50*^*+/y*^, *Gpr50*^*−/y*^, and *Gpr50*^*−/y*^ mice were subjected to behavioral tests. The experimenter was blinded to the genotype during testing. All behavioral tests were performed in a dimly lit room without noise interference. The experimental apparatus used in every test was cleaned with 30% alcohol after each tested subject.

### Three-chamber social preference test

Three-chamber social preference test was described previously [68]. The tested mice were placed in a transparent three-chamber box and allowed to shuttle freely for 10 min. Then, two empty metal cages were placed on both sides of the transparent box. The mice were allowed to explore the three chambers freely for 10 min. Then, one of the metal cages was placed in a stranger mouse of the same age and sex. The test mice were allowed to explore for 10 min among the three chambers, and the time spent by the test mice in the lateral chamber containing either stranger mice or empty cages and the time spent interacting with either stranger mice or empty cages were recorded. In the final stage, another unfamiliar mouse was placed in a metal cage on the other side, and the time spent in the lateral chamber containing either the unfamiliar or familiar mouse and the time spent interacting with the unfamiliar or familiar mouse was recorded.

### Buried food test

The tested mice were individually housed for more than 24 h, followed by a three-day acclimatization period. All food was removed for 24 h before testing, but water was freely available. After this period, the mice were placed in a clean rat cage for 5 min of acclimatization. Following this, the mice were removed, the bedding was replaced, and food was buried 3 cm under the bedding in the lower right corner of the cage (the same corner was used in every trial). The tested mice were then placed into the cage with the buried food, and the cage was covered. An observer moved 2 meters away from the cage. The mouse was placed back in the center of the rat cage and given a maximum of 15 min to find the food. The time the mouse took to find the food (indicated by the mouse uncovering it, picking it up, or starting to eat it) was recorded in seconds. If the mouse didn’t find the food within 15 min, the time was recorded as 15 min.

### Statistical analysis

All data analyses were performed using GraphPad Prism 8.0 to produce graph values. All graph values are presented as mean ± SEM. All statistical analyses were performed using SPSS 26.0 or GraphPad Prism 8.0. The number of mice included in each experiment was based on standards established in the literature rather than being predetermined by statistical methods. Tests for normality and equal variances were used to determine the appropriate statistical test to employ. Data were analyzed using Student’s t-test (a comparison of the difference between the two groups) after confirming the homogeneity of variance and normal distribution and one-way ANOVA (a comparison between multiple groups) followed by LSD or Dunnett T3 post hoc tests. Statistical details of experiments such as statistical tests, statistical values, and the information related to n can be found in the figure legends. Significance in differences was accepted at *p* < 0.05 (*p < 0.05; **p < 0.01; ***p < 0.001).

### Supplementary information

Supplementary Figure 1

Supplementary Figure 2

Supplementary Figure 3

Supplementary information

## Data Availability

All data supporting this study are presented in this published article and in its Supplementary information files. Raw data of all results can also be acquired form figshare database (http://figshare.com) 10.6084/m9.figshare.26014606.
