# Supplementary figures and images for: GPR50 regulates neuronal development as a mitophagy receptor
Source: Cell Death Dis. 2024 Aug 15;15(8):591. doi: 10.1038/s41419-024-06978-y (PMC11324738; doi:10.1038/s41419-024-06978-y)

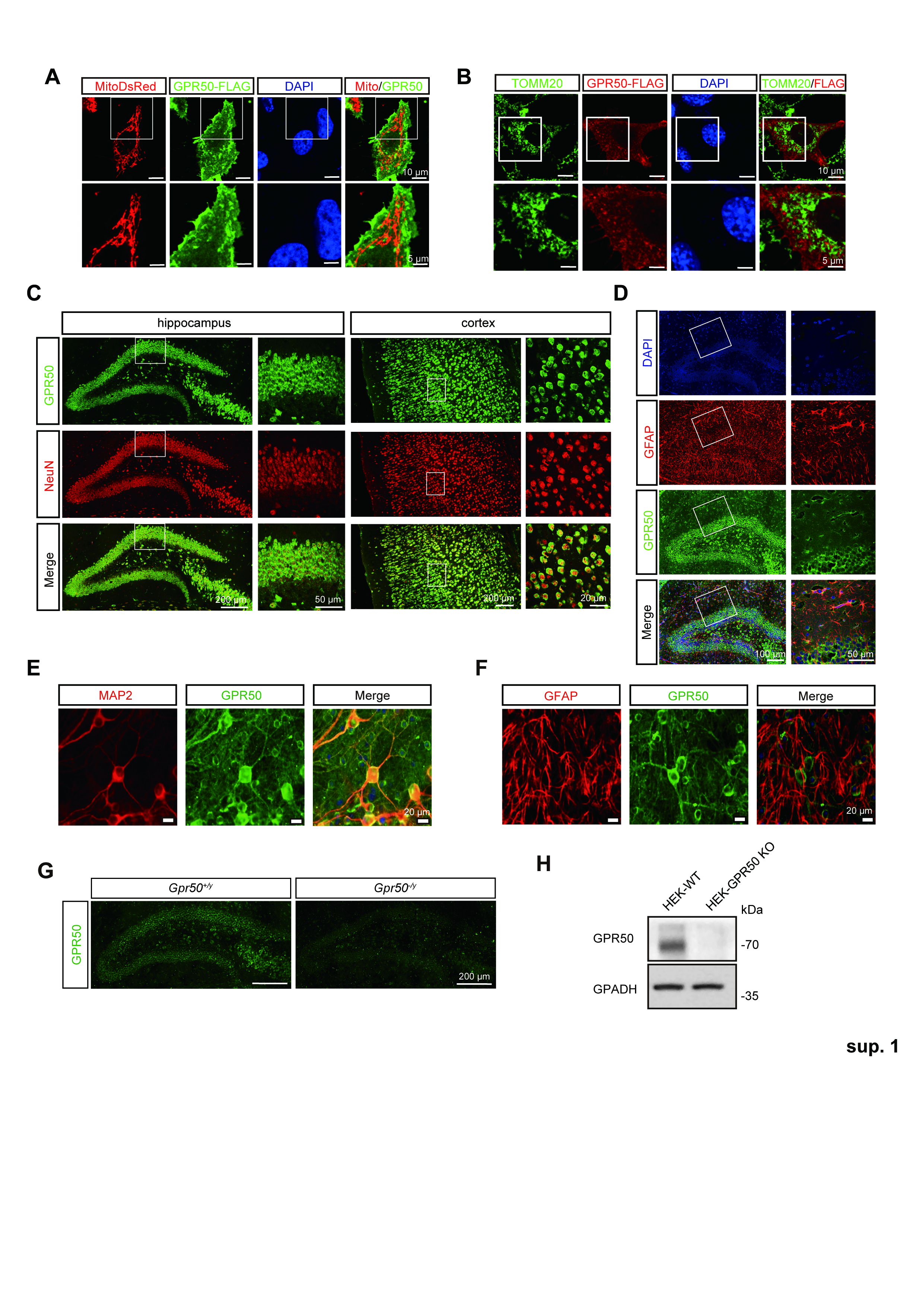

Supplement: Supplementary file 1 — Supplementary Figure 1 [file 41419_2024_6978_MOESM1_ESM.tif]

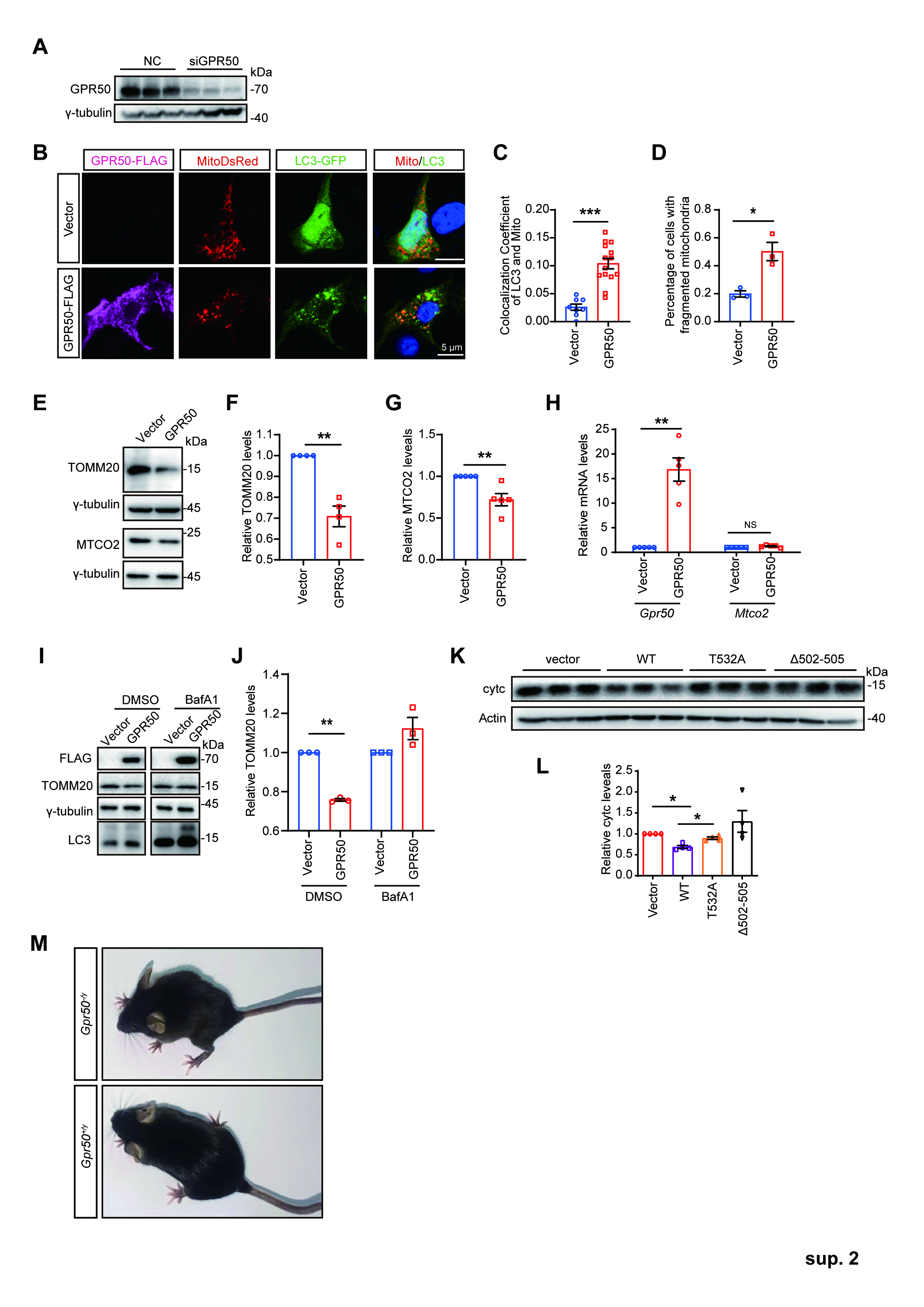

Supplement: Supplementary file 2 — Supplementary Figure 2 [file 41419_2024_6978_MOESM2_ESM.tif]

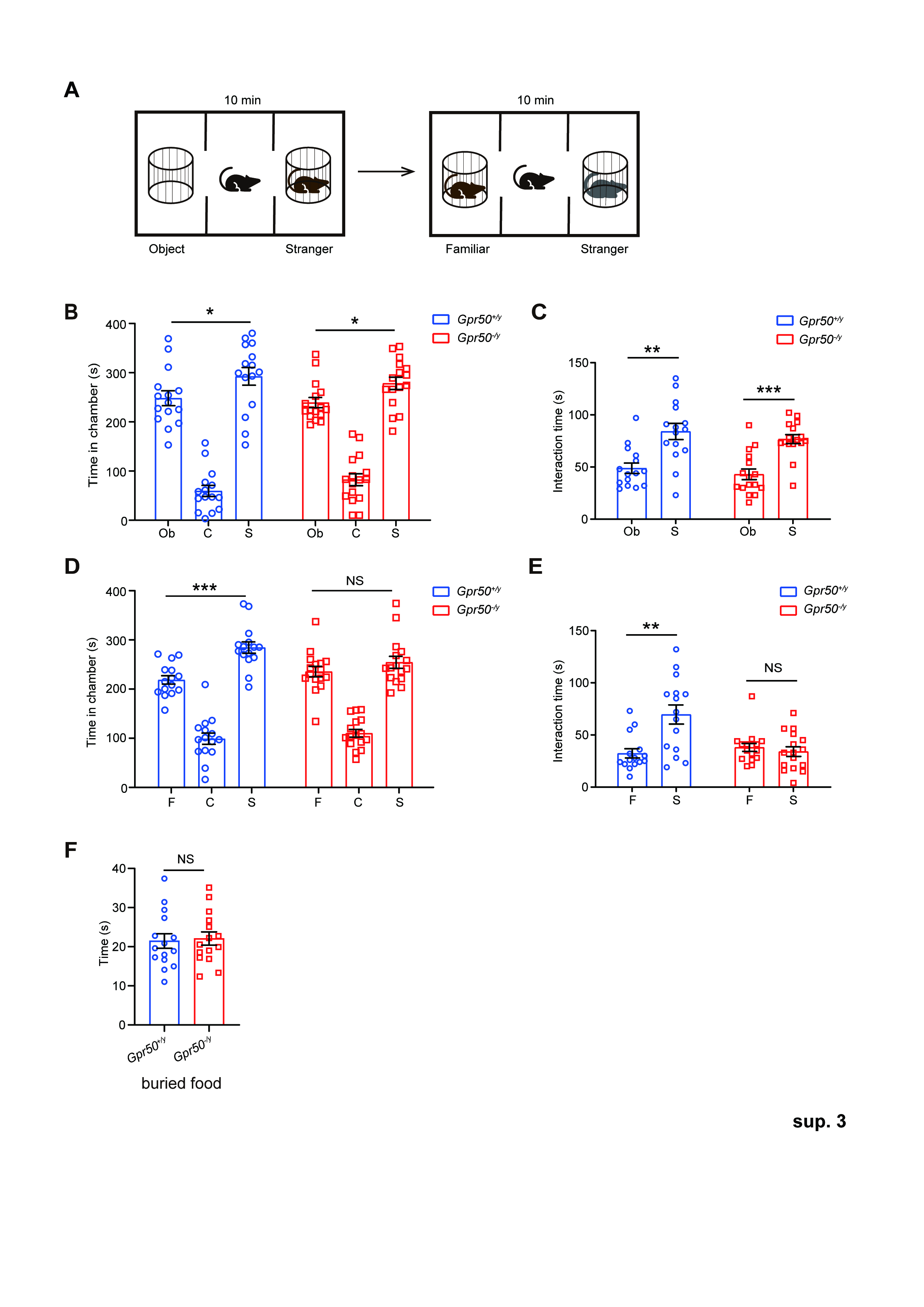

Supplement: Supplementary file 3 — Supplementary Figure 3 [file 41419_2024_6978_MOESM3_ESM.tif]
