# Supplementary material for: GPR50 regulates neuronal development as a mitophagy receptor
Source: Cell Death Dis. 2024 Aug 15;15(8):591. doi: 10.1038/s41419-024-06978-y (PMC11324738; doi:10.1038/s41419-024-06978-y)
Supplement: Supplementary file 4 — Supplementary information [file 41419_2024_6978_MOESM4_ESM.pdf]

Supplementary Figure 1 Expression of GPR50 in mouse brain.

(A, B) HEK293 cells transfected with GPR50-FLAG and mito-RFP were immunostained for FLAG and TOMM20. Scale bars: 5 μm.

(C-F) The coronal sections of the hippocampus and cortex of adult C57/BL6 mice (C, D) and the cultured primary neurons were co-immunostained for GPR50, NeuN, MAP2, or GFAP (E, F). Scale bars: 200 μm in the images with lower magnification, 50 μm (hippocampus) and 20 μm (cortex) in the images with higher magnification.

(G, H) Detection of GPR50 in *Gpr50*$^{+/y}$ or *Gpr50*$^{-/y}$ mice and HEK-GPR50 KO cells using immunofluorescence staining (G) and western blotting (H). Scale bars: 200 μm.

Supplementary Figure 2 Overexpression of GPR50 enhances mitophagy.

(A) Western blotting detection of GPR50 Knockdown efficiency by SiRNA.

(B-D) HEK293 cells co-transfected with mito-RFP, LC3-GFP, and GPR50-FLAG were immunostained for FLAG and imaged. Scale bars: 5 μm. Colocalization Coefficient of Mito-RFP and LC3-GFP (C). Percentage of cells with fragmented mitochondria (D).

Student's t-test for colocalization Coefficient ($t_{19.316} = -7.134$, $p = 0.000$). n = 8-14 cells from 3 independent experiments (C). Student's t-test for percentage of cells ($t_4 = -4.367$, $p = 0.012$). n =3 independent experiments (D).

(E-J) Western blotting analysis of TOMM20 and MTCO2 levels in GPR50-FLAG transfected HeLa cells (E), which were treated with Baf-A1 (I, J). Relative levels of TOMM20 (F, J) and MTCO2 (G). Q-PCR analysis of mRNA levels of *Gpr50* and *Mtco2* (H).

Student's t-test for TOMM20 ($t_6 = 5.913$, $p = 0.001$) (F), MTCO2 ($t_8 = 3.835$, $p = 0.005$) (G), Gpr50 ($t_4 = -6.739$, $p = 0.001$), *Mtco2* mRNA ($t_4 = -2.285$, $p = 0.084$) (H). n = 4-5 independent experiments (F-H).

Student's t-test for TOMM20-DMSO ($t_2 = 36.685$, $p = 0.001$), TOMM20-Baf-A1($t_2 = -2.163$, $p = 0.163$). n =3 independent experiments (I, J).

(K, L) Western blotting analysis of Cytc levels in WT GPR50-FLAG or 502-505 truncation (Δ502-505), T532A transfected in HEK293T cells (K). Relative Cytc levels (L).

One-way ANOVA ($F_{3,12} = 3.686$, $p = 0.043$) followed by Dunnett T3 post hoc tests for Vector versus WT ($p = 0.011$), WT versus T532A ($p = 0.022$), WT versus Δ502-505 ($p = 0.329$). n =3

independent experiments (K, L).

(M) Body size of adult *Gpr50*$^{-/y}$ and *Gpr50*$^{+/y}$ mice.

Supplementary Figure 3 Deficiency of GPR50 results in defective social behaviors in mice.

(A-E) Three chamber tests.

(A) Schematic description of three-chamber tests.

(B-E) Three chamber tests in *Gpr50*$^{+/y}$ and *Gpr50*$^{-/y}$ mice. Time the mice spent in chambers containing either Object (Ob in B) or Familiar mice (F in D) versus Stranger (S in B, D) mice. Time the mice spent interacting with either Object (Ob in C) or Familiar mice (F in E) versus Stranger (S in C, E) mice.

One-way ANOVA ($F_{5, 87}$ = 57.586, p = 0.000) followed by LSD post hoc tests for *Gpr50*$^{+/y}$ Object versus *Gpr50*$^{+/y}$ Stranger (p = 0.024), *Gpr50*$^{-/y}$ Object versus *Gpr50*$^{-/y}$ Stranger (p = 0.038) (B). Student's t-test for *Gpr50*$^{+/y}$ (t_{28} = -3.850, p = 0.001) and *Gpr50*$^{-/y}$ (t_{30} = -5.076, p = 0.000) (C). One-way ANOVA ($F_{5, 87}$ = 54.627, p = 0.000) followed by LSD post hoc tests for *Gpr50*$^{-/y}$ Familiar versus *Gpr50*$^{+/y}$ Stranger (p = 0.000), *Gpr50*$^{-/y}$ Familiar versus *Gpr50*$^{-/y}$ Stranger 2 (p = 0.199) (D). Student's t-test for *Gpr50*$^{+/y}$ ($t_{20.205}$ = -3.661, p = 0.002) and *Gpr50*$^{-/y}$ (t_{30} = 0.657, p = 0.516) (E). n = 15-16 mice per genotype (B-E).

(F) Buried food test. Student's t-test (t_{28} = -0.249, p = 0.805). n = 15 mice per genotype (F).

Data are presented as mean ± SEM. **p*<0.05; ***p*<0.01; ****p*<0.001. n.s, non-significance.

Supplementary materials and methods

Antibodies. Anti-MAP2 antibody (Abnova, MAB11155; 1:200 for immunofluorescence); anti-FLAG antibody (Sigma-Aldrich, F3165; 1:3000 for western blot; 1:500 for immunofluorescence); anti-TOMM20 antibody (abcam, AB186734, 1:1000 for western blot; 1:200 for immunofluorescence); anti-synaptophysin (SYN) antibody (abcam, ab32127; 1:100 for immunofluorescence); anti-GPR50 antibody (Proteintech Group, 21514-1-AP; 1:1000 for western blot; 1:100 for immunofluorescence); anti-GPR50 antibody (Cell Signaling Technology, 14032S; 1:1000 for western blot); anti-LC3B antibody (Novus Biologicals, NB100-2220; 1:1000 for western blot); anti-γ-tubulin antibody (Sigma-Aldrich, T6557; 1:3000 for western blot); anti-MTCO2 antibody (abcam, ab198286; 1:1000 for western blot); anti-Timm23 antibody (Sigma-Aldrich, HPA031408; 1:1000 for western blot); anti-GAPDH antibody (Sigma-Aldrich, SAB1405848; 1:2000 for western blot); anti-GFP antibody (Sigma-Aldrich, G1544; 1:1000 for western blot); anti-β-tubulin antibody (abcam, ab209866; 1:3000 for western blot); anti-Cytochrome c (cytc) antibody (Anclonal Technology, Ab110325; 1:1000 for western blot); anti-NeuN (Cell Signaling Technology, 12943S; 1:500 for immunofluorescence); anti-GFAP antibody (Cell Signaling Technology, 80788S; 1:500 for immunofluorescence); anti-mouse IgG peroxidase (abcam, ab7003; 1:5000 for western blot); anti-rabbit IgG peroxidase (Thermo Fisher Scientific, A-21206; 1:5000 for western blot); anti-mouse Alexa Fluor 555 (Invitrogen, A-32773; 1:1000 for immunofluorescence); anti-rabbit Alexa Fluor 594 (Invitrogen, A-21207; 1:1000 for immunofluorescence); anti-mouse Alexa Fluor 488 (Invitrogen, A-21202; 1:1000 for immunofluorescence); anti-rabbit Alexa Fluor 488 (Thermo Fisher Scientific, R37118; 1:1000 for immunofluorescence); anti-mouse Alexa Fluor 647 (Thermo Fisher Scientific, A21235; 1:1000 for immunofluorescence); anti-rabbit Alexa Fluor 647 (Thermo Fisher Scientific, A31573; 1:1000 for immunofluorescence); anti-goat Alexa Fluor 647 (Merck Millipore, AP180SA6; 1:1000 for immunofluorescence); anti-goat Alexa Fluor 555 (abcam, ab150130; 1:1000 for immunofluorescence).

Immunofluorescence staining. For immunostainings, cells were fixed with 4% PFA at room temperature for 15 min and permeabilized with 0.3% Triton X-100 for 5 min. For immunostaining on brain sections, the cryosections of mouse brains were washed 3 times with PBS containing 0.5% Triton X-100. Then, the brain sections or the fixed cells were incubated with 10% FBS for 1 h at room temperature to block non-specific binding. Next, the diluted primary antibodies were added to the sections or cells and incubated at 4 °C overnight. After washing, the brain sections or cells were incubated with the corresponding secondary antibodies at room temperature for 1 h, and then mounted with a medium containing DAPI (SouthernBiotech, 0100-20). Images were acquired by a Laser confocal microscope (ZEISS, LSM 900). The images were analyzed with Image-ProPlus 6.0 image analysis software (Media Cybernetics, MD, USA).

Western Blotting Analysis. Brain homogenates were extracted with brain lysis buffer (10 mM of Tris-HCl, pH 9.0, 150 mM of NaCl, 1% Sodium deoxycholate, 0.5% Triton X-100, 0.5% SDS, 2 mM EDTA) supplemented with protease inhibitor cocktail (Roche, 11697498001). For cultured cells, the cells were lysed with RIPA lysis buffer (Beyotime, P0013D) containing a protease inhibitor cocktail for 30 min on ice. Samples were centrifuged at 14000 g for 15 min at 4 °C; supernatants were collected. Protein extracts were subjected to SDS-PAGE electrophoresis and transferred to the PVDF membrane. Following a standard protocol, the PVDF membrane (Merck Millipore, IPVH00010) was incubated with a primary antibody and the corresponding horseradish peroxide (HRP)-conjugated secondary antibody. The protein signals were detected by ECL (Merck Millipore, WBKLS). The images were captured with an E-Gel Imager (E-Blot, China). The intensity of each protein signal on the membrane was measured as grayscale values using Image J software (NIH, Bethesda, MA, USA), corrected for background intensity, and normalized to the internal control.

mRNA extraction and quantitative transcription-polymerase chain reaction (qPCR). Total cellular RNA was isolated using Trizol Reagent (Sigma-Aldrich, T9424).

An equal amount of the first-strand cDNAs were synthesized with the FastQuant RT Kit (Tiangen Biotech, KR108). PCR reactions were performed with Taq DNA polymerase (Takara, RR02MQ). The following primers were used. Human *GPR50*: CTGAGCCAGTTACAGTGCCAGATG (Forward), GCGATTGCCACGATGTTGAAGATG (Reverse); Human *GAPDH*: TCGGAGTCAACGGATTTGGT (Forward), TTCCCGTTCTCAGCCTTGAC (Reverse).

Fig1. A

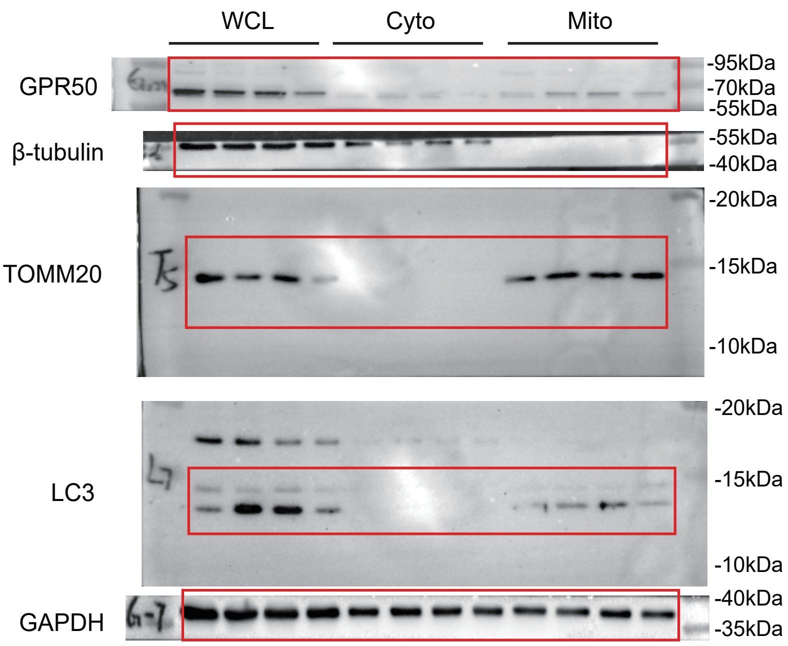

Fig2. I

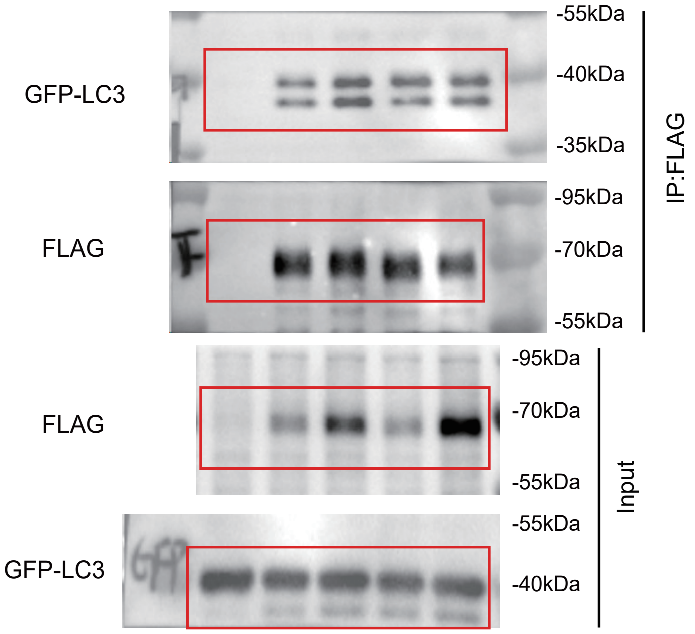

Fig2. K

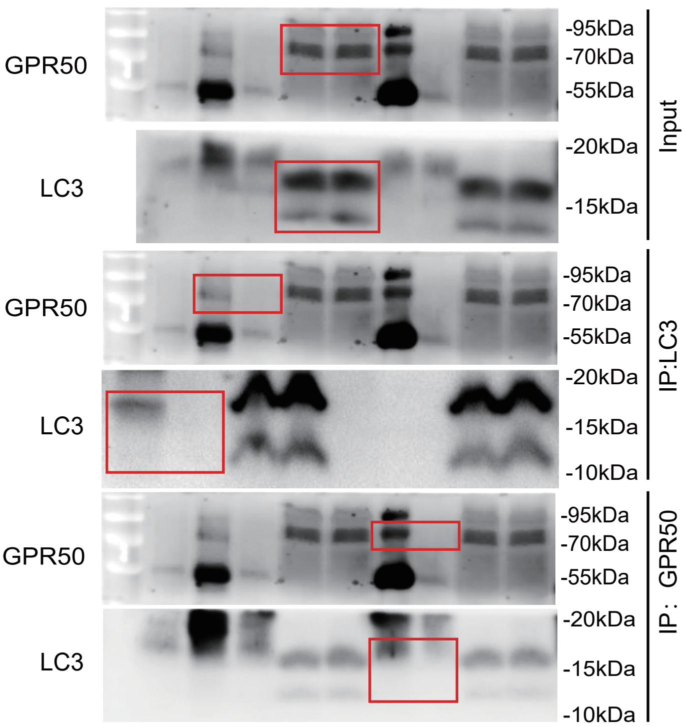

Fig3. A

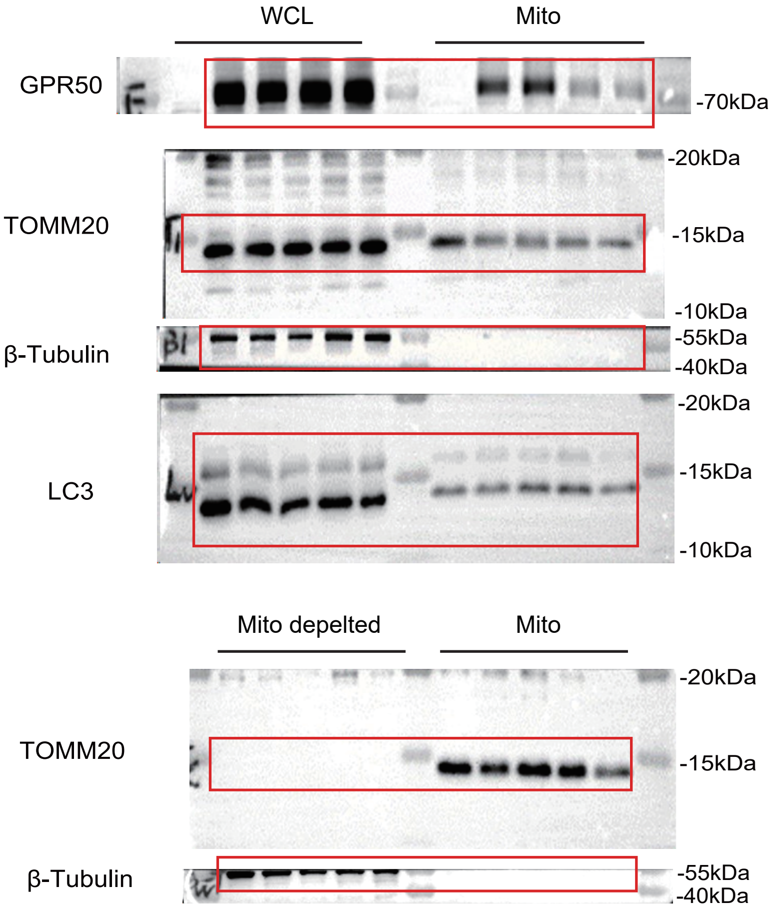

Fig4. D

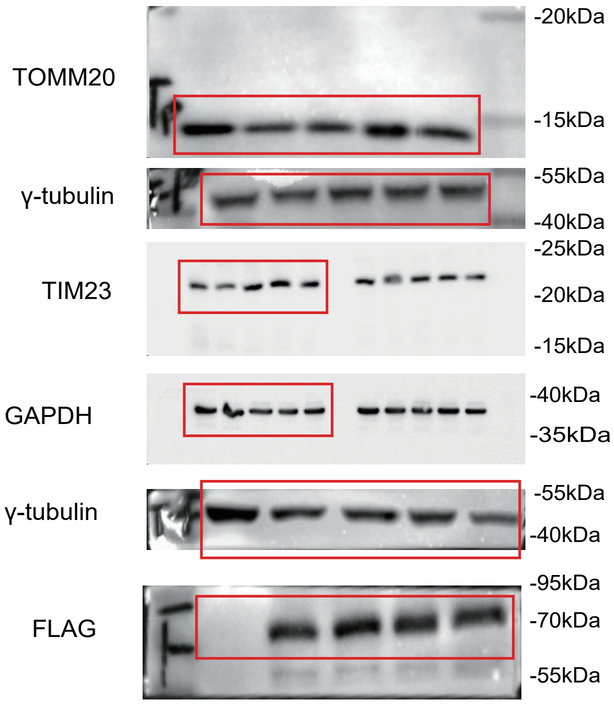

Fig4. G

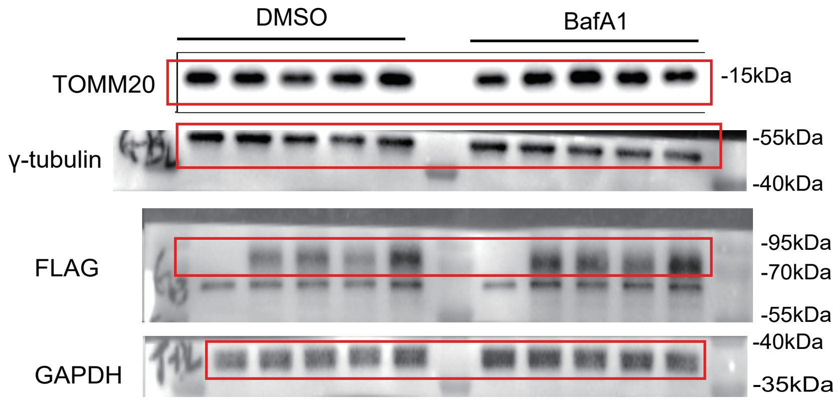

Fig5. G

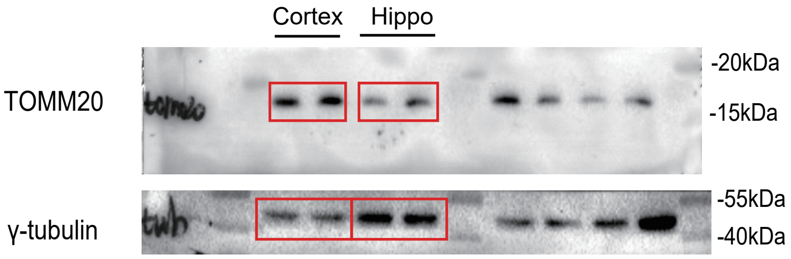

Sup1. H

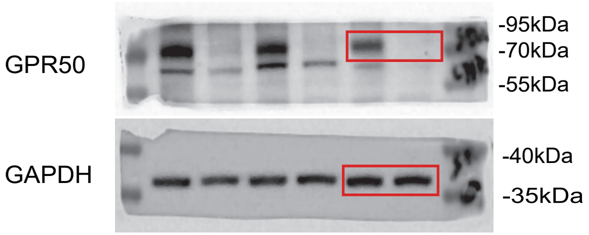

Sup2. K

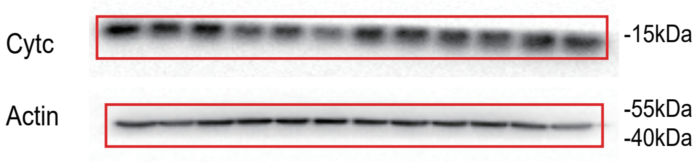

Sup2. A

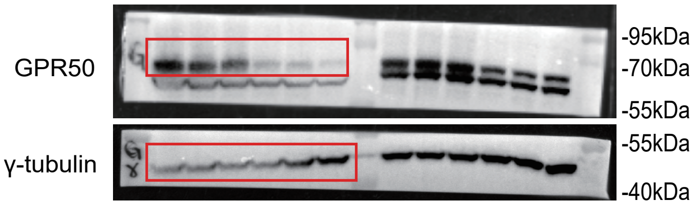

Sup2. I

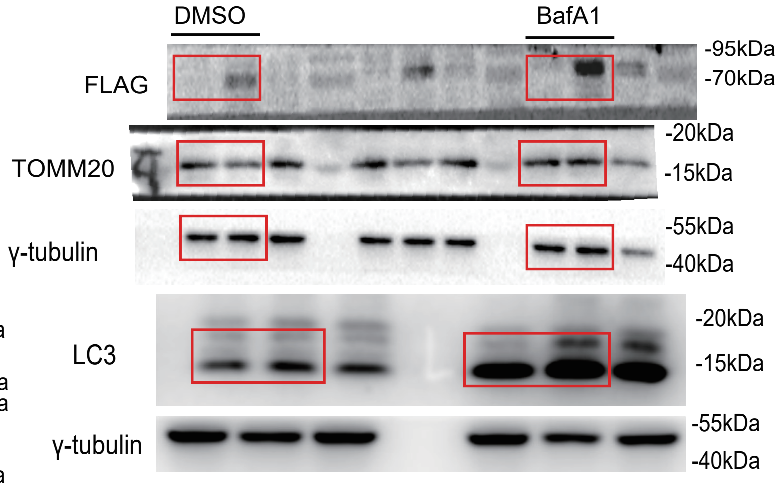

Sup2. E

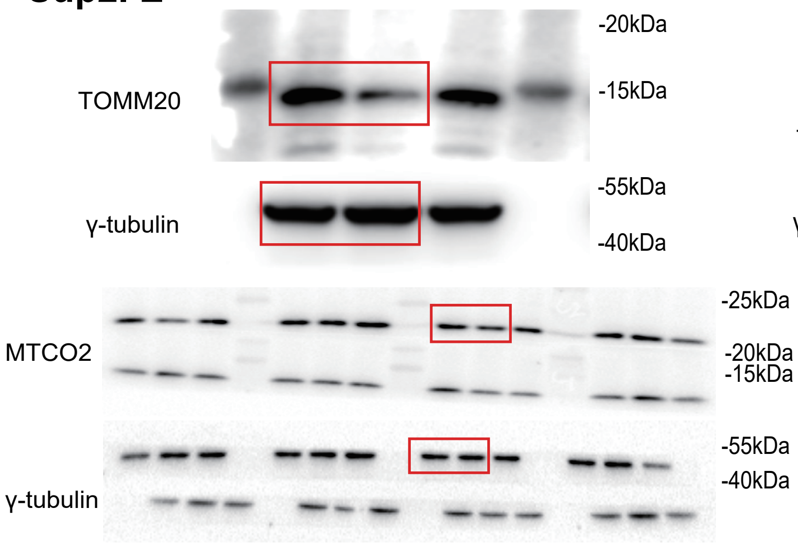

Sup. 5

Raw Data

Figure 1B

western blot of TOMM20

CCCP-0h	CCCP-2h	CCCP-6h	CCCP-12h
1	0.841782	0.796116	0.726066
1	0.948639	0.873151	0.634886
1	0.867274	0.73595	0.449409
1	0.890494	0.935777	0.803687
1	0.928595	0.875624	0.747456

Figure 1C

western blot of GPR50

mitocondrial

0	1	1	1	1	1
2	1.224364	1.27886	1.271024	1.59868	1.23719
6	1.50169	2.564283	1.651956	2.63321	1.89678
12	1.237434	1.93811	1.435871	1.50611	0.97085

WCL

0	1	1	1	1	1
2	1.298374	0.833995	1.129983	1.05814	0.85418
6	1.652076	0.942873	1.187833	0.94452	1.32602
12	1.392859	0.708925	1.089061	0.88694	1.07318

Figure 1D

western blot of LC3 II

mitocondrial

0	1	1	1	1	1
2	1.57361	1.314801	1.391432	1.28324	1.52406
6	2.912127	2.215425	3.123946	2.21127	2.01525
12	1.705409	0.799197	1.603625	1.0222	0.90278

WCL

0	1	1	1	1	1
2	1.67361	1.314801	1.591432	1.58324	1.62406
6	1.912127	1.549927	2.261496	2.07875	2.01525
12	1.705409	0.799197	1.603625	1.0222	0.90278

Figure 1F

Colocalization coefficients between GPR50 and TOMM20

Vector	cccp 2h	cccp 6h	cccp 12h	cccp 24h	cccp 36h	cccp 48h
0	0.152079	0.024064	0.139199	0.06932	0.11863	0.05345
0	0.158902	0.227187	0.103087	0.08444	0.06442	0.05566
0	0.037363	0.250028	0.096593	0.04201	0.05942	0.08745
0	0.220765	0.306075	0.153153	0.05343	0.031	0.23719
0	0.379342	0.43642	0.11525	0.09927	0.03442	0.05686
0	0.1123	0.37822	0.274272	0.08862	0.06377	0.06205
0	0.144694	0.308002	0.11937	0.18544	0.0703	0.24894
0	0.111798	0.420853	0.101923	0.23373	0.0383	0.12466
0	0.076475	0.127077	0.093905	0.14733	0.05501	0.03274
0	0.135218	0.066178	0.295147	0.06902	0.0528	0.06368
0	0.249819	0.07643	0.0838	0.08563	0.06009	0.07898
0	0.178301	0.294605	0.085286	0.08566	0.14526	0.06321
0	0.149775	0.360642	0.20895	0.14651	0.11957	0.10612
0	0.161561	0.432855	0.273259	0.12358	0.08502	0.03703
0	0.05889	0.493891	0.303108	0.07245	0.01432	0.11082
0	0.117118	0.145182	0.367967	0.06988	0.02805	0.02278
	0.130078	0.028818	0.182597	0.05594	0.03702	0.02462
	0.062826	0.114366	0.188667	0.04853	0.07726	0.12461
	0.069424	0.273134	0.222729	0.15082	0.04875	0.02613
	0.07235	0.208143	0.064434	0.24714	0.05936	0.02368
	0.069424	0.296229	0.195755	0.13251	0.37275	0.00731
	0.068143	0.248252	0.075241	0.02088	0.22962	0.13528
	0.10169	0.124061	0.094394	0.02508	0.1329	0.17078
	0.10194	0.124061	0.093786	0.02154	0.12755	0.06179
		0.148413	0.102539	0.01807	0.05901	0.05702
		0.27578	0.340955	0.03808	0.12109	0.06893
		0.188698	0.334606	0.02312	0.12764	0.04738
		0.137151	0.132995	0.02858	0.11924	0.01191
			0.047131	0.13692	0.16135	0.05418
				0.07599	0.12776	0.13396
				0.11288	0.13958	
				0.06164	0.03815	
				0.1415		
				0.03628		

Figure 2F

ELISA analysis of LIR and LC3

	H_2O			LIR			LIR-mut		
0	0.0745	0.0577	0.0912	0.5834	0.51	0.5023	0.4881	0.5181	0.4683
1	0.0582	0.0489	0.0648	0.5974	0.5186	0.4731	0.4002	0.3992	0.4191
2.5	0.0525	0.0675	0.0356	1.186	1.1147	1.1487	0.3958	0.4183	0.3605
5	0.0517	0.0721	0.0466	3.0967	3.1288	3.3292	0.4055	0.4316	0.4424
10	0.0498	0.0291	0.0783	3.5306	3.7011	3.8365	0.3886	0.4302	0.4567
20	0.0518	0.0437	0.0387	3.625	3.6403	3.8343	0.4099	0.4611	0.406
40	0.0489	0.0535	0.0598	3.4192	3.6807	3.9111	0.5549	0.5967	0.4306

Figure 2H

PLA of GPR50 binding LC3

Ctrl	WT	mLIR	Δ502-505	T532A	Ctrl	WT	mLIR	Δ502-505	T532A
1	33	7	13	19	0	18	5	24	14
1	37	5	17	15	0	17	9	17	11
1	20	15	19	12	0	19	13	13	17
1	30	10	12	29	0	23	3	11	11
1	33	12	18	16	0	23	4	15	20
1	25	6	11	24	3	19	9	12	25
0	31	5	19	13	2	22	12	13	19
0	29	7	13	16	2	16	7	17	18
0	28	8	19	29	1	20	3	14	18
0	34	7	7	15	1	21	6	15	25
0	24	2	14	25	1	28	9	9	9
0	22	2	20	27	1	24	7	19	22
0	20	4	9	14	0	17	3	12	10
0	22	4	10	18	0	30	10	9	17
0	29	5	11	26	0	39	7	14	19
0	28	2	21	16	0	23	3	21	11
0	27	5	10	17	0	25	2	9	19
0	20	3	9	15	0	22	3	16	16
0	32	8	28	20	0	23	17	8	17
2	26	4	21	16	0	29	7	14	19
2	28	3	18	21	0	25	6	15	17
1	25	3	13	21	0	22	11	13	15
1	21	2	8	14	0	15	6	14	13
0	23	2	10	14	0	36	6	17	14
0	24	2	10	16	0	22	4	15	14
0	32	10	15	14	0	35	6	9	14
0	41	4	11	10	0	18	7	20	16
0	32	7	15	17	0	21	2	16	16
0	31	14	13	14	0	17	11	13	11
0	29	4	19	15	0	20	3	18	21
0	30	5	13	17	0	21	7	12	15
0	21	2	21	23	0	36	6	11	23
0	27	4	17	19	0	20	4	20	13
0	25	4	14	14	2	18	4	23	21
0	32	2	13	18	1	26	5	27	18
0	30	4	10	18	1	20	14	16	11
0	32	4	16	10	1	29	2	21	16
0	19	3	17	16	0	25	3	19	25
1	23	10	17	14	0	20	7	22	15
1	20	3	20	18	0	23	9	15	14
1	33	8	12	21	0	16	11	26	16
1	25	14	10	11	0	12	5	24	12
1	27	8	8	21	0	18	5	18	11
0	25	5	14	15	0	24	9	31	22
0	33	5	13	16	0	16	7	13	12
0	21	8	12	13	0	21	10	16	13
0	25	5	11	10	0	17	2	13	16
0	27	3	17	13	0	32	3	20	13
0	20	3	13	13	2	18	12	17	17
0	15	3	23	15	2	27	2	15	13
0	21	11	27	14	1	29	8	14	8
0	24	5	19	17	1	16	4	17	8
0	14	4	16	13	1	20	6	16	12
0	21	13	20	12	0	18	10	10	15
0	25	5	20	18	0	30	2	9	19
0	23	8	16	16	0	18	5	8	15
2	28	3	16	14	0	22	10	15	14
2	22	4	17	10	0	28	3	9	11
2	21	5	26	16	0	24	9	12	17
2	15	5	18	12	0	36	5	20	10
1	27	5	19	15	4	36	6	16	15
1	25	4	13	11	3	41	14	14	18
1	31	10	16	15	2	22	9	15	11
1	23	8	17	9	2	45	3	11	13
1	21	7	15	17	1	31	5	12	21
0	33	6	21	19	1	24	6	15	13
0	20	4	17	12	1	43	4	18	19
0	20	10	13	13	0	18	8	9	22
0	16	10	17	17	0	20	4	24	17
0	39	7	18	23	0	33	8	14	25
0	36	8	17	26	0	22	11	16	11
0	25	8	14	22	0	24	3	11	16
0	23	6	13	23	0	21	5	13	
0	18	5	15	20	0	43	3	13	
0	27	11	9	21	0	32		15	
2	30	2	14	25	0	21		23	
2	26	8	15	15	2	21		14	
3	35	13	15	17	2	30		18	
1	38	6	10	28		28		19	
1	29	7	15	19		37			
1	27	7	11	16		24			
0	21	4	16	10		31			
0	21	3	17	9					
0	27	9	13	24					
0	40	8	12	19					
0	33	7	15	13					
0	22	9	17	13					

Figure 2J

CO-IP of GFP-LC3 pulled down by GPR50

Vector	WT	mLIR	Δ502-505	T532A
0	1	0.44899	0.564714	0.7067
0	1	0.70058	0.638979	0.6252
0	1	0.81607	0.870689	0.843
0	1	0.61869	0.760232	0.7478

Figure 3B

western blot of GPR50

mitochondria

WT	1	1	1	1	1
mLIR	1.09566	1.08104	0.867927	1.1006	0.67541
△502-505	0.70985	0.65154	0.842357	0.5752	0.70729
T532A	0.39392	0.71369	0.613543	0.8517	0.58903

whole cell lysate

WT	1	1	1	1	1
mLIR	0.98423	0.94615	0.945629	0.9683	1.01536
△502-505	1.01628	0.82453	1.132843	0.9808	1.05953
T532A	0.95039	0.89795	1.126032	0.9016	0.90415

Figure 3D

Pearson coefficient of GPR50 and TOMM20

WT	mLIR	Δ502-505	T532A
0.228888	0.28326	0.03002	0.016438
0.249011	0.03553	0.23457	0.053651
0.234774	0.27763	0.11383	0.122386
0.260906	0.34389	0.14453	0.134768
0.203667	0.13184	0.07817	0.091534
0.252722	0.28791	0.01374	0.081676
0.183099	0.21498	0.0477	0.110768
0.236974	0.18336	0.01557	0.017261
0.192737	0.16008	0.15817	0.25764
0.299239	0.09457	0.10091	0.067792
0.229574	0.19059	0.15542	0.275401
0.196558	0.13477	0.07137	0.105312
0.296916	0.03813	0.35508	0.073582
0.300475	0.11176	0.37595	0.227727
0.194874	0.20453	0.13555	0.09691
0.205625	0.114	0.17268	
	0.34787	0.2168	
	0.05557	0.01221	
	0.08744	0.15728	
	0.18162	0.071	
		0.19953	
		0.19931	
		0.05462	
		0.32625	
		0.01136	

Figure 4B

Colocalization coefficient of LC3 and Mito

DMSO		CCCP	
NC	siGPR50	NC	siGPR50
0.07521	0.01949	0.225322	0.05588
0.11575	0.0061	0.192936	0.06872
0.08376	0.1292	0.177681	0.06687
0.0964	0.04123	0.166823	0.01608
0.07575	0.08988	0.20692	0.04387
0.10729	0.14901	0.104487	0.07663
0.0994	0.09771	0.081283	0.07589
0.08342	0.06234	0.121884	0.03085
0.10286	0.08113	0.176821	0.03326
0.05771	0.06934	0.206387	0.06227
0.10586	0.0542	0.096801	0.01122
0.07911	0.02147	0.066903	0.08546
0.13159	0.02047	0.187004	0.0485
0.06291		0.096387	0.04468
0.03028		0.239115	0.07259
		0.269198	0.07005
		0.187677	0.046
		0.188747	
		0.294492	

Figure 4C

Percentage of cells with fragmented mitochondria

DMSO		CCCP	
NC	siGPR50	NC	siGPR50
0.192	0.206	0.96	0.88
0.1	0.25	1	0.812
0.206	0.219	1	0.821

Figure 4E

western blot of TOMM20

Vector	WT	mLIR	Δ502-505	T532A
1	0.67271	1.14186	1.340757	1.29177
1	0.75501	1.37971	1.139408	1.30395
1	0.66454	1.43171	1.529313	1.60637

Figure 4F

western blot of TIM23

Vector	WT	mLIR	Δ502-505	T532A
1	1.11501	0.95163	1.11593	1.08967
1	1.08027	0.94107	1.07814	1.04041
1	1.11071	0.93359	1.09654	1.07474

Figure 4H

western blot of TOMM20

	DMSO				Baf-A1	
Vector	1	1	1	1	1	1
WT	0.87306	0.83135	0.86177	1.50924	1.7305	1.36748
mLIR	1.17916	1.39167	1.22728	1.07877	1.52516	1.20794
△502-505	1.12596	1.24484	1.28333	1.76201	1.45084	1.55472
T532A	1.46888	1.25292	1.20369	1.51901	1.319	1.82113

Figure 4J

Pearson coefficient between mito-RFP and GFP-LC3

Vector	WT	mLIR	Δ502-505
0.0423	0.16747	0.19732	0.06122
0.02432	0.27456	0.13221	0.0049
0.03377	0.24249	0.10894	0.13227
0.01916	0.36818	0.14216	0.00382
0.1119	0.28453	0.02598	0.01108
0.01561	0.16384	0.05763	0.0496
0.10737	0.37465	0.38647	0.04615
0.10782	0.25619	0.0509	0.10508
0.04513	0.28787	0.05232	0.09028
0.08325	0.3307	0.13989	0.23057
0.00783	0.19872	0.07176	0.22912
0.10113	0.26514	0.02532	0.06539
0.00837	0.1132	0.07111	0.06753
0.21573	0.09856	0.1662	0.13595
0.09664	0.16914	0.18846	0.13251
0.09693	0.0692	0.15883	0.09046
0.08032	0.31933	0.12221	0.14638
0.13564	0.17637	0.28756	0.13712
0.02638	0.38345	0.37608	0.15813
	0.21318	0.13201	0.01347
	0.3368	0.03757	0.23391
	0.23368		0.06562
	0.16156		0.04625
	0.18036		0.27562
	0.17232		0.31524
	0.21041		0.33016
	0.13048		0.1646
	0.50575		0.04673
			0.36157
			0.21586

Figure 4K

GFP [+]Mito[+]FLAG[+] puncta per cell

WT	mLIR	Δ502-505	T532A
11	2	1	3
7	2	2	3
5	2	3	3
10	3	4	4
11	3	2	5
7	3	5	3
11	5	2	4
15	4	3	4
15	4	4	2
9	4	4	3
13	5	4	1
11	6	4	3
20	7	4	4
12	3	5	3
12	4	7	4
6	6	5	5
7	2	4	4
10	4	3	4
15	7	1	7
10	4	4	3
10	5	3	3
10	3	4	3
7	4	4	3
10	4	5	2
7	4	5	3
7	10	4	4
7	4	2	4
17	4	3	2
15	6	2	3
8	4	3	4
10	7	5	7
8	3	3	4
12	4	3	2
15	4	6	3
12	6	3	3
10	6	2	5

Figure 4L

Pearson coefficient between FLAG⁺ and GFP-LC3⁺

WT	mLIR	Δ502-505	T532A
0.36208	0.09401	0.08066	0.48509
0.3904	0.43737	0.22969	0.16984
0.54058	0.31688	0.26756	0.49398
0.54579	0.17061	0.1813	0.57224
0.52532	0.10449	0.24571	0.45629
0.10354	0.15562	0.23284	0.58457
0.51047	0.18238	0.54022	0.38872
0.36951	0.15912	0.57753	0.01084
0.62898	0.09562	0.32613	0.1469
0.59524	0.11695	0.03065	0.00369
0.45078	0.51643	0.39581	0.17267
0.43111	0.45623	0.04833	0.48025
0.3107	0.04579	0.56648	0.42099
0.56068	0.2563	0.17367	0.16528
0.40321	0.11776	0.24221	0.70679
0.40617	0.41149	0.41463	0.15667
0.59625	0.74999	0.2181	0.44802
0.51983	0.03515	0.28877	0.2766
0.42987	0.38461	0.23545	0.41603
0.24338	0.41332	0.36809	0.08124
0.32033	0.26002	0.21536	0.48289
0.47307	0.56866	0.11152	0.17406
0.46888	0.5598	0.42439	0.39004
0.68029	0.61709	0.36269	0.44546
0.36901		0.21254	0.44114
0.74902		0.31444	0.19958
0.50738		0.33042	0.32934
0.29253		0.207	0.53834
0.64842		0.33201	0.41415
0.72432		0.15571	0.24208
0.47443		0.45308	0.51474
0.18512		0.71801	0.45265
0.63073		0.54901	0.39522
0.74207		0.47936	0.55842
0.66659			0.13843
0.34543			0.01675
0.61465			0.13526
			0.12286

Figure 5C

TMRE for HEK cells

Hek-WT	Vector	WT	mLIR	Δ502-505	T532A
56.965	12.206	59.754	7.129	7.836	31.889
52.203	7.145	18.178	12.206	7.867	34.476
49.427	9.27	39.842	7.145	7.224	47.298
50.885	13.592	38.517	13.592	7.654	4.791
49.082	11.297	40.708	11.297	8.061	7.769
43.538	12.52	17.014	12.52	8.774	5.915
52.216	6.921	16.107	6.824	6.891	5.978
49.779	7.846	44.032	7.846	8.805	10.733
41.769	11.937	15.221	11.937	13.168	15.408
44.638	7.341	52.154	7.341	8.802	24.645
52.24	16.094	44.032	16.094	15.793	14.379
49.682	13.214	53.757	13.214	9.219	14.437
43.817	7.533	18.806	7.533	9.4	22.92
45.4	7.126	20.835	7.067	9.207	18.211
51.555	8.475	18.35	8.475	13.406	14.942
45.297	3.995	19.565	7.344	7.465	16.503
45.167	7.121	19.741	7.121	9.002	16.118
67.074	45.371	30.67	46.009	6.533	25.699
65.668	37.934	57.249	43.234	8.11	14.452
64.785	46.087	62.617	43.319	48.875	16.543
64.409	43.353	59.949	45.186	49.162	15.726
64.696	45.997	60.311	38.54	45.657	47.619
64.742	13.794	53.746	43.596	50.546	41.385
61.204	50.487	58.191	43.55	49.983	43.98
65.01	35.103	62.949	40.027	53.171	46.013
65.218	36.693	53.339	38.477	37.727	49.475
35.744	39.761	56.566	42.298	53.269	43.028
29.68	36.241	54.464	43.453	46.054	44.507
37.328	37.117	62.19	43.467	31.211	42.244
44.409	41.012	38.54	44.032	39.961	30.159
40.869	26.663	43.596	39.842	40.072	31.311
37.706	24.596	43.55	38.517	35.952	44.298
27.024	44.733	42.041	40.708	33.365	38.63
26.288	12.339	38.477	42.315	36.327	41.856
27.054	10.509	42.298	42.222	36.049	36.111
28.919	12.297	43.453	36.516	11.854	28.282
26.045	8.204	43.467	42.911	11.131	15.057
31.893		62.617	35.329		
60.311		59.949	38.83		
53.746		55.829	40.894		
58.191		52.422	33.958		
62.949			32.131		
53.339					
56.566					
54.464					

Figure 5D

TMRE for primary neurons

$G^{+}Y$	Vector	WT	mLIR	Δ502-505	T532A
12.31	7.94	11.7	16.46	4.9	5.18
9.8	3.75	7.48	7.57	9.73	10.22
13.44	8.57	9.6	16.94	7.93	10.15
8.18	19.35	13.43	16.92	20.96	4.59
44.98	18.45	15.19	13.56	6.62	18.52
16.11	6.61	13.97	7.48	8.64	4.22
14.67	19.62	25.32	4.89	7.54	8.67
13.17	11.26	13.51	18.81	6.83	6.61
6.84	7.94	20.13	10.82	11.11	19.37
11.35	4.6	17	19.09	10.72	15.36
21.95	10.53	19.95	4.38	15.52	12.02
33.69	4.9	15.57	11.97	8.57	13.49
30.44	4.37	7.95	10.35	6.44	13.26
23.29	9.95	6.98	6.93	10.14	11.18
31.78	6.56	17.36	7.78	4.06	5.6
13.14	24.33	36.89	14.73	4.59	26.49
15.37	23.82	25.35	7.17	4.29	11.9
42.78	22.51	7.89	9.2	3.79	7.49
14.66	4.74	11.56	17.45	6.51	4.65
24.56	26.1	13.68	11.61	5.14	4.91
11.54	12.4	5.21	13.09	4.15	4.41
44.93	22.27	14.41	11.94	4.69	13.11
16.48	13.65	6.66	15.42	4.4	13.01
46.27	16.34	21.86	11.66	4.11	7.3
56.69	12.02	13.08	7.18	3.52	3.6
20.72	16.27	23.75	17.53	3.45	5.13
18.37	8.08	21.05		4.98	8.14
26.25	12.35	12.09		6.15	4.76
21.88	22.45	11.96		7.16	20.28
24.11	8.97	37.51		14.97	22.86
13.24	11.79	15.17		30.84	23.63
9.29	5.67	15.65		16.13	26.75
28.2	11.73	9.55		16.17	
23.79	4.56	7.01			
10.71	11.46	16.62			
10.8	15.72	11.99			
19.38	8.71	22.38			
15.49	12.17	33.33			
10.95	11.91	26.12			
18.73	11.25	31.06			
13.39	2.37	32.81			
24.22	16.94	15.48			
8.09	13.05				
11.55	6.2				
10.32	11.14				
11.24					
17.25					
17.62					

Figure 5F

TEM analysis of mitochondria in $GPR50^{+/Y}$ and $GPR50^{-/Y}$ mice

$Gpr50^{+}/y$		$Gpr50^{-}/y$	
Normal	Swollen	Normal	Swollen
0.25	0.5625	0.33333	0.66667
0.5	0.33333	0.2	0.8
0.466667	0.33333	0.33333	0.66667
0.5	0.33333	0.33333	0.33333
0.6	0.4	0.28571	0.57143
0.833333	0.16667	0.28571	0.42857
0.714286	0.28571	0.33333	0.66667
0.777778	0.11111	0.25	0.75
0.8	0	0.375	0.5
0.625	0.375	0.25	0.625
0.333333	0.16667	0	0.8
0.818182	0.09091	0.16667	0.83333
0.666667	0.22222	0.11111	0.77778
0.818182	0.09091	0.44444	0.55556
0.7	0.2	0.25	0.5
0.785714	0.14286	0.33333	0.5
0.363636	0.27273	0.2	0.4
0.545455	0.36364	0.25	0.75
0.416667	0.58333	0.46154	0.53846
0.681818	0.18182	0.38462	0.61539
0.272727	0.72727	0.25	0.5
0	0.75	0.2	0.8
0.375	0.5625	0.26667	0.66667
0.472222	0.5	0.3	0.7
0.166667	0.75	0.25	0.66667
0.222222	0.66667	0	1

Figure 5H

western blot of TOMM20

	Cortex			Hippocampus		
WT	1	1	1	1	1	1
KO	1.34216	1.20416	1.55132	1.16226	1.26972	1.22107

Figure 5J

OCR

Gpr50 $^{+/y}$

cortex	PGM	9.9707	14.8606	13.471	30.3253	15.3398	29.9977
	ADP	23.9076	49.5271	44.3896	51.1395	34.3208	47.1039
	S	49.4437	80.341	67.2055	73.8676	87.3157	70.6461
	ROT	37.5823	46.5835	39.7136	37.7936	92.2522	88.7563
	AS+TMPD	172.711	172.499	153.729	222.318	216.05	180.734
hippocampus	PGM	15.3398	29.9977	14.0363	13.3089	11.3289	13.0079
	ADP	34.3208	47.1039	38.3907	35.3531	32.5583	35.7012
	S	87.3157	70.6461	66.7957	67.3744	74.472	66.8259
	ROT	64.8494	56.7307	46.4988	41.5099	52.5023	51.8847
	AS+TMPD	216.05	180.734	199.049	160.547	148.209	198.385

gpr50 $^{-/y}$

cortex	PGM	8.8193	13.7022	12.2193	24.767	11.2464	24.3522
	ADP	18.617	39.7433	27.5408	38.9187	23.946	38.7319
	S	41.8491	67.2164	53.7214	64.0207	58.9681	71.6519
	ROT	35.005	39.6993	35.3402	39.0405	59.9295	76.7142
	AS+TMPD	147.625	141.566	131.893	199.769	188.708	144.399
hippocampus	PGM	11.2464	24.3522	5.5738	10.4498	10.6363	12.4199
	ADP	23.946	38.7319	7.5145	27.0082	29.4267	25.3982
	S	58.9681	71.6519	25.2233	58.4326	64.8014	66.2698
	ROT	43.5493	54.7823	21.5693	38.7032	44.9544	51.6454
	AS+TMPD	188.708	144.399	141.919	151.08	136.575	169.299

Figure 5K

The max ETS capacity

cortex						
Gpr50 $^{+/y}$	56.3889	84.0905	70.0038	68.3389	92.2522	88.7563
gpr50 $^{-/y}$	47.005	70.8977	59.7004	62.928	59.9295	76.7142
hippocampus						
Gpr50 $^{+/y}$	92.2522	88.7563	78.6465	73.8681	80.6496	71.7812
gpr50 $^{-/y}$	59.9295	76.7142	28.9214	64.7863	68.6528	71.9443

Figure 5L

spare respiratory capacity

cortex					
Gpr50 $^{+/y}$	5.4686	4.4956	3.9941	3.3847	5.1662
gpr50 $^{-/y}$	1.9765	3.6499	3.5107	3.8857	4.2127
hippocampus					
Gpr50 $^{+/y}$	5.1662	5.4686	4.5087	4.3618	4.3864
gpr50 $^{-/y}$	1.9684	4.5494	3.4035	3.8857	4.2127

Figure 5M

ATP levels

293T	KO	WT	mLIR	Δ502-505	T532A
1	0.306834	0.5599	0.24753	0.32866	0.39186
1	0.305109	0.62493	0.48814	0.42992	0.45211
1	0.313346	0.7626	0.65308	0.64319	0.58878
1	0.39253	0.68239	0.57554	0.57502	0.5499
1	0.361286	0.61871	0.44841	0.44488	0.45743
1	0.39404	0.62374	0.49619	0.50229	0.44488

Figure 5O

ROS levels in HEK-WT and GPR50-KO cells

Hek-293T	Vector	WT	mLIR	Δ502-505	T532A
8.97	26.5	7.88	8.66	10.39	9.38
7.15	7.39	7.59	15.64	9.05	24.42
7.24	25.19	8.87	9.4	10.74	19.92
7.21	7.57	11.41	12.23	12.85	12.92
6.23	7.34	13.12	12.57	10.68	14.58
10.83	8.44	12.68	10.89	8.6	18.71
7.27	8.7	13.81	17.89	11.32	11.39
6.16	7.1	9.35	12.75	8.94	20.68
7.11	31.878	8.56	9.01	8.91	12.73
7.35	7.32	10.64	16.04	11.72	14.07
7.64	8.57	7.34	24.46	14.03	14.02
7.64	10.74	8.87	35.09	9.28	10.86
7.93	7.4	7.55	26.93	12.57	9.31
7.83	17.33	6.02	14.59	13.26	7.99
7.34	12.11	9.31	11.3	9.17	12.2
7.23	8.78	8.07	12.14	11.32	10.79
7.91	19.48	12.79	14.44	26.18	10.5
7.92	7.83	8.91	9.78	24.7	13.05
9.1	17.98	8.6	9.1	25.26	11.26
8.89	8.89	7.82	14.38	17.33	10.69
8.97	36.5	7.4	9.13	28.1	11.46
9.03	31.75	7.26	10.99	13.21	9.39
7.9	30.22	7.33	10.5	27.17	10.05
7.79	23.25	7.7	30.89	21.97	10.67
14.81	33.37	7.71	27.06	29.06	10.84
7.42	32.46		14.44		
7.22			13.56		
11.19			23.61		
11.78			30.63		
			39.55		
			38.48		

Figure 5Q

ROS levels in primary neurons

Gpr50 $^{+/y}$	*gpr50* $^{-/y}$	*gpr50* $^{-/y}$ + mitoQ
33.0419	32.2366	22.27529
29.4436	34.1793	20.33446
18.7656	34.0227	17.24051
29.5627	36.4193	29.73736
26.1581	30.0967	18.93607
26.5629	36.1959	18.86918
23.7343	25.6828	17.27168
24.1629	20.7334	20.57757
24.2144	27.78	20.932
29.6801	24.4617	12.75902
20.5106	13.3238	22.57153
16.972	27.3898	34.33945
27.3852	33.5829	17.30914
19.5025	31.3996	18.73634
5.31634	18.5433	21.62832
25.0711	33.5611	11.38856
8.27334	33.9369	9.995916
28.6208	31.2524	8.92248
25.1414	33.9369	12.59013
8.81099	33.5611	11.03403
19.8749	38.8855	5.971649
28.8346	29.1854	20.08487
25.759	36.3585	6.037732
26.8451	30.2892	19.74738
10.3105	10.8307	8.142531
33.6142	13.1539	16.36288
24.6201	37.613	20.53396
6.24034	37.613	31.72386
25.3455	27.9862	31.31571
8.31834	49.0695	32.13622
23.7772	17.5122	37.10197
27.9925	37.2395	34.11978
15.2162	43.9224	9.200317
26.9312	42.2167	18.9296
16.3341	36.7595	30.05401
25.1268	36.9719	21.04679
9.56873	32.8442	20.5941
	32.3755	27.00146
	33.0761	28.07657
	36.5329	
	38.8671	
	37.7431	
	46.0745	
	29.9756	

Figure 5R

MitoROS levels in the hippocampal

Gpr50 $^{'/y}$	*gpr50* $^{-/y}$	*gpr50* $^{-/y}$ + mitoQ
1	1.20047	0.8976051
1	1.58169	1.140895
1	1.47147	1.191332

Figure 6B

Total length of neuronal dendrites(GPR50$^{+/Y}$,GPR50$^{-/Y}$ and GPR50$^{-/Y}$ +WT)

G$^{'}$/Y	G^{-}/Y	G^{-}/Y+GPR50
1098.27	1299.58	1314.671
2647.83	1326.69	912.206
2699.05	573.069	1235.04
2340.11	854.518	796.05
2348.72	1329.74	1572.677
2001.02	1673.11	841.214
2012.18	744.816	2077.77
2974.99	978.937	682.352
2054.62	1261.08	2970.063
2797.13	635.204	1396.674
2110.88	694.657	2226.163
2393.95	760.364	1721.491
2709.7	1070.05	1249.495
1587.11	502.465	774.516
4144.29	1011.84	1347.09
2494.36	1157.84	
2158.17	573.565	
1624.26	849.131	
1334.97	2477.26	
2683.15	995.163	
	1074.56	
	981.543	
	1335.02	
	899.53	
	1218.08	
	1234.68	
	884.538	
	908.9	
	776.989	
	1565.71	

Figure 6C

Total number of neuronal dendrites (GPR50$^{+/Y}$,GPR50$^{-/Y}$ and GPR50$^{-/Y}$ +WT)

G$^{'}$/Y	G^{-}/Y	G^{-}/Y+GPR50
12	2	6
14	2	4
11	2	5
5	4	6
7	6	5
9	4	5
7	2	8
9	2	4
7	4	4
9	7	4
6	2	3
5	3	5
12	3	4
4	2	6
14	2	3
10	3	
10	2	
10	3	
3	9	
8	3	
	4	
	4	
	3	
	2	
	3	
	4	
	2	
	2	
	3	
	3	

Figure 6D

Numbers of neuronal dendrites (GPR50$^{+/Y}$,GPR50$^{-/Y}$ and GPR50$^{-/Y}$ +WT)

G+/Y

10	1	1	1	1	4	1	1	1	2	1	1	1	4	1	2	1	5
20	1	1	2	1	4	1	1	1	2	1	1	1	6	1	1	4	10
30	11	1	9	1	5	1	1	1	2	1	1	3	5	2	2	7	16
40	9	2	13	1	11	1	4	1	7	1	3	8	2	4	4	7	11
50	13	6	5	6	8	3	2	1	3	3	3	6	8	6	10	8	16
60	6	8	13	6	12	3	4	1	6	4	4	11	14	10	10	7	24
70	9	5	14	9	18	3	4	4	10	4	6	13	12	12	8	11	20
80	8	6	9	8	14	4	4	5	6	11	6	20	12	13	7	14	18
90	9	10	14	9	9	7	6	8	9	13	7	28	17	11	7	10	9
100	6	7	10	13	6	7	11	10	7	19	10	22	11	7	12	7	12
110	6	7	9	17	3	6	10	11	9	17	11	18	10	5	12	8	11
120	8	9	8	18	3	7	11	11	9	8	9	14	11	9	14	4	7
130	5	10	9	24	4	5	10	13	8	4	9	9	9	8	18	10	9
140	9	6	8	13	3	5	6	12	7	8	6	16	9	6	21	6	7
150	7	6	9	11	4	8	7	13	8	8	9	8	12	10	14	9	9
160	9	8	6	8	6	8	7	12	9	7	7	9	7	6	16	9	7
170	4	6	10	12	3	7	5	14	6	6	11	9	12	5	18	7	11
180	6	4	13	12	4	5	6	14	5	8	6	8	11	4	11	8	8
190	7	8	13	8	5	8	11	10	8	7	11	13	10	5	14	9	8
200	8	6	17	12	8	9	9	18	7	8	9	11	15	8	16	8	7
210	5	7	14	9	7	7	11	19	9	10	9	11	13	8	16	5	5
220	2	11	16	7	9	10	10	19	6	10	12	11	15	9	19	7	4
230	4	8	12	7	8	10	10	20	9	12	12	9	14	6	13	11	7
240	3	9	13	10	7	10	8	16	7	10	11	13	23	6	17	5	3
250	3	9	11	5	11	6	8	20	7	9	14	13	16	7	24	8	8
260	3	11	14	9	9	9	13	22	9	12	13	15	25	8	18	9	6
270	3	11	14	11	17	7	11	14	10	8	12	15	21	7	18	3	5
280	5	12	13	7	12	6	9	18	12	6	17	15	14	14	24	8	11
290	4	12	14	8	14	5	11	18	9	8	14	10	17	4	23	9	9
300	5	8	16	8	15	5	8	26	7	6	15	7	23	2	21	7	6

G^{-}/Y

10	1	1	1	1	1	2	1	7	9	4	6	1	1	1
20	1	1	1	1	1	1	1	5	12	11	8	1	1	1
30	2	3	3	1	1	4	2	5	8	11	9	2	2	2
40	4	4	2	1	3	2	1	4	7	6	12	2	5	5
50	3	4	2	3	4	5	6	2	4	3	11	5	7	7
60	4	5	4	3	6	6	4	3	9	4	10	4	6	6
70	8	6	10	2	11	9	4	2	4	4	7	4	4	4
80	9	8	8	5	4	7	3	3	4	4	3	3	5	5
90	12	6	7	3	4	7	8	3	3	5	2	4	4	4
100	6	3	3	4	5	7	7	2	4	3	4	4	2	2
110	7	2	2	3	4	11	5	3	3	3	6	4	5	5
120	6	3	2	4	7	11	7	1	3	3	4	5	5	5
130	5	2	3	5	4	12	6	4	3	4	3	2	2	2
140	5	3	2	3	4	10	5	3	3	3	2	3	4	4
150	7	2	2	7	3	8	9	4	3	4	4	3	1	1
160	4	2	3	5	6	5	7	2	4	3	3	5	2	2
170	4	2	4	6	3	8	10	6	3	3	2	4	3	3
180	9	2	8	7	3	5	6	3	2	4	3	4	2	2

190	7	5	5	7	2	3	5	2	3	6	2	2	2	2
200	3	3	4	3	1	5	6	1	3	3	2	3	2	2
210	4	4	4	5	3	9	10	3	6	3	3	2	1	1
220	3	3	9	4	3	11	5	3	3	3	2	1	2	2
230	7	3	7	3	4	8	8	2	2	3	3	1	0	1
240	4	5	5	2	4	9	1	1	4	2	3	1	1	1
250	5	8	9	3	6	7	8	1	4	4	1	1	1	1
260	7	1	1	7	1	5	8	2	5	7	3	1	1	1
270	1	5	1	5	5	3	1	1	3	5	2	1	1	1
280	1	7	6	2	1	9	6	5	4	5	4	1	1	1
290	8	3	8	2	6	1	4	1	4	4	1	1	1	1
300	1	6	8	6	4	9	5	9	7	3	2	1	1	1

G-/Y+GPR50

10	1	3	1	1	1	1	4	3	6	2	2	1	2	5	3	1
20	1	7	1	1	3	1	3	6	2	1	1	6	3	9	8	3
30	1	5	1	1	6	3	4	8	1	1	6	7	1	8	9	7
40	1	10	2	1	6	4	3	9	3	1	7	8	4	2	7	7
50	3	8	5	2	7	5	4	2	3	6	6	5	4	2	5	5
60	4	7	5	3	10	4	8	9	3	5	5	4	6	3	7	9
70	8	2	13	7	6	7	5	9	10	11	4	6	4	4	6	7
80	9	3	8	7	2	8	5	13	16	8	5	7	3	4	5	9
90	9	3	4	9	2	11	4	14	4	10	7	4	7	1	5	5
100	12	3	14	4	2	6	4	4	2	8	4	3	2	1	4	5
110	10	3	14	4	3	11	5	3	4	11	6	5	5	2	4	10
120	7	4	10	5	4	16	4	5	6	13	6	4	8	2	3	9
130	3	4	11	6	5	10	5	7	5	11	8	3	3	2	8	10
140	3	3	8	6	5	12	6	4	10	14	7	4	5	1	4	6
150	4	2	8	6	7	15	8	4	9	10	4	5	9	3	8	6
160	3	4	15	3	5	10	7	5	9	7	5	5	8	2	10	8
170	3	5	16	5	11	9	7	7	7	12	6	3	7	3	5	7
180	2	3	22	3	9	10	8	6	7	14	9	5	5	2	3	6
190	2	5	21	7	5	8	7	9	4	10	7	6	8	2	3	4
200	2	4	19	3	8	12	5	3	6	15	5	8	4	3	3	6
210	2	2	12	7	7	7	10	6	4	12	12	11	5	1	3	7
220	2	2	14	4	6	7	9	6	5	7	7	11	5	2	1	7
230	2	5	12	3	4	6	10	6	5	12	7	13	6	2	2	7
240	3	2	19	3	8	10	10	6	5	6	14	11	6	3	4	4
250	6	2	13	5	10	5	7	2	2	3	10	11	7	3	6	4
260	5	2	12	2	6	6	5	1	6	6	11	7	6	3	2	7
270	4	2	9	4	8	9	6	2	9	12	5	12	8	2	3	6
280	3	4	12	10	9	8	4	4	7	4	9	14	7	7	3	8
290	4	4	10	7	3	9	4	5	5	6	13	11	8	4	2	8
300	5	4	14	5	1	7	5	4	6	2	11	7	4	10	3	5

Figure 6F

Total length of neuronal dendrites ($GPR50^{-/Y}$ or $GPR50^{-/Y}$ mice were transfected with GPR50-FLAG WT, mLIR, Δ502-505 or T532A)

Vector	WT	mLIR	Δ502-505	T532A
405.978	2751.81	851.056	564.933	381.056
709.8	1997.75	689.957	781.351	792.049
1363.9	3893.69	226.824	562.756	441.91
952.246	1312.17	981.938	620.638	691.607
865.286	1228.7	409.546	713.587	1090.72
805.727	1476.21	106.84	566.266	419.099
525.977	539.769	1125.82	517.55	351.311
269.008	2349.59	835.134	489.931	1206.35
533.374	585.802	1036.02	532.613	207.65
555.131	732.289	656.484	576.069	277.238
1110.26	1473.85	406.651	501.416	670.3
219.586	757.963	308.532	537.237	921.773
326.084	611.061	440.392	563.607	787.011
505.744	2144.74	294.338	335.687	829.338
509.259	946.483	317.735	680.061	839.159
239.117	1258.89	276.218	627.778	451.519
291.533	860.971	380.877	481.231	322.287
380.008	1275.6	545.214	385.987	576.048
579.534	905.3	433.884	809.16	226.739
195.013	1831.07	277.417	1006.13	500.891
482.056	1830.99	453.795	433.102	497.64
140.243	1603.56	388.542	930.89	352.486
841.403	2137.44	760.799	946.853	553.565
498.023	1939.52	371.041	630.336	627.497
888.218	2043.75	714.168	305.314	614.477
229.308	798.214	511.914	509.762	392.924
371.334	1323.28	1742.9	516.515	777.062
	939.347	981.042	618.215	867.003
	1379.73	593.865	231.67	1313.96
	1380.42	435.347	668.247	296.417
	2184.67	1298.55		
	2198.37	535.505		
	1789.64	1009.32		
	744.842	675.187		
		3275.01		
		1537.43		
		545.725		
		419.379		

Figure 6G

Total number of neuronal dendrites ($GPR50^{-/Y}$ or $GPR50^{-/Y}$ mice were transfected with GPR50-FLAG WT,mLIR, Δ502-505 or T532A)

Vector	WT	mLIR	Δ502-505	T532A
2	5	3	3	2
2	5	2	2	2
3	9	3	3	2
3	5	2	3	3
3	3	3	3	3
4	3	2	3	2
3	3	2	3	2
1	5	3	4	3
2	3	3	4	2
2	4	2	2	3
2	4	3	2	2
2	5	2	5	4
3	4	3	5	3
4	5	2	2	3
2	4	2	5	3
2	7	2	5	3
3	5	3	2	1
2	3	3	3	3
3	4	3	3	2
2	4	1	3	2
4	5	2	3	2
2	7	4	3	3
5	7	3	2	3
3	7	3	3	5
4	6	2	2	6
2	2	4	4	3
3	4	3	4	4
	3	5	2	4
	3	4	1	5
	4	3	3	
	7	1		
	6	4		
	4	2		
	2	3		
		3		
		2		
		4		

Figure 6H

Numbers of neuronal dendrites (GPR50$^{-/Y}$ or GPR50$^{-/Y}$ mice were transfected with GPR50-FLAG WT,mLIR， Δ502-505 or T532A)

PC

10	1	2	2	2	3	2	2	1	2
20	3	2	2	2	3	2	2	3	2
30	4	2	2	3	3	2	2	4	3
40	4	2	2	3	3	2	3	5	3
50	4	2	2	3	3	3	3	5	3
60	4	2	2	3	3	3	3	5	3
70	3	0	2	3	3	3	3	5	3
80	2	0	2	3	3	3	3	5	3
90	1	0	2	3	3	3	3	4	3
100	1	0	2	3	3	2	3	4	3
110	1	0	2	3	2	1	3	4	3
120	1	0	2	3	2	0	3	4	3
130	1	0	2	3	2	0	3	3	3
140	1		2	3	2		3	2	3
150	1	0	2	6	2	0	3	1	6
160	1	0	1	1	2	0	2	0	1
170	1	0	1	1	2	0	2	0	1
180	0	0	1	1	2	0	2	0	1
190	0	0	1	1	2	0	2	0	1
200	0	0	1	1	2	0	2	0	1

WT

10	2	2	2	2	1	1	2	1	2
20	2	2	2	4	1	1	1	2	2
30	2	2	4	5	2	1	1	3	1
40	2	3	5	5	2	1	1	5	1
50	8	3	5	4	7	3	1	6	1
60	4	3	5	4	7	4	2	6	1
70	5	3	6	4	8	4	2	6	1
80	3	3	7	4	9	4	2	7	1
90	2	3	7	6	11	4	3	5	2
100	2	3	7	4	10	4	3	5	2
110	2	3	7	4	7	4	3	5	3
120	2	3	7	4	9	4	3	4	3
130	2	3	7	4	9	4	3	4	3
140	3	3	7	4	9	4	3	5	3
150	3	2	7	4	9	4	3	5	3
160	2	2	7	4	9	3	3	5	3
170	2	2	6	2	9	3	3	6	3
180	2	2	6	3	7	3	2	6	3
190	2	2	6	3	7	3	2	4	3
200	2	2	6	3	7	3	2	3	3

mLIR

10	1	2	1	1	2	1	2
20	2	2	2	2	2	3	2
30	2	2	3	3	2	4	3
40	3	2	4	6	2	4	3
50	3	2	4	5	2	4	4
60	3	2	4	5	2	4	4
70	3	2	4	5	2	4	4
80	3	2	4	5	2	4	4
90	3	2	4	5	2	4	4
100	3	2	4	5	2	4	4
110	3	2	3	5	2	4	4
120	2	2	3	4	2	4	4
130	2	2	3	3	2	2	4
140	2	2	3	3	2	2	4
150	2	2	2	3	2	2	4
160	2	2	1	3	2	2	4
170	2	2	1	3	2	2	4
180	2	2	1	2	2	2	4
190	2	4	1	1	1	1	4
200	2	1	1	1	1	1	4

Δ502-505

10	2	2	4	1	1
20	2	1	4	1	1
30	2	1	4	1	1
40	4	1	4	2	2
50	4	1	4	3	3
60	4	1	4	3	3
70	4	1	4	3	3
80	4	2	4	3	3
90	2	4	4	3	3
100	2	3	4	3	3
110	1	3	3	3	3
120	1	3	3	3	3
130	1	3	3	3	3
140	1	3	3	3	3
150	1	1	3	3	3
160	1	1	3	2	2
170	1	1	2	2	2
180	1	1	2	1	2
190	1	1	2	1	2
200	1	1	2	1	2

T532A

10	2	2	1	1	1	1	1
20	2	2	4	1	2	2	1
30	2	2	5	3	3	2	1
40	3	2	5	4	3	4	1
50	3	2	5	5	3	3	2
60	3	2	5	5	3	3	3
70	3	2	5	5	3	4	3
80	3	2	5	5	3	4	4
90	3	2	5	5	3	4	5
100	3	2	5	4	3	4	5
110	3	2	5	4	3	4	5
120	1	2	4	4	3	3	5
130	1	2	4	4	3	3	5
140	1	2	4	3	3	3	5
150	1	2	4	3	3	5	4
160	1	2	4	3	3	3	3
170	1	2	2	3	2	3	3
180	1	2	2	3	2	3	2
190	1	2	1	2	2	3	2
200	1	2	0	1	2	3	2

Figure 7B

Time in chamber-Ob+S1

Gpr50 $^{+/y}$

Ob	268	305	144	264	312	181	272	243	201	258	180	199	225	234
C	38	180	82	25	40	52	47	26	77	76	43	48	69	92
S1	294	115	374	311	248	367	281	331	322	266	377	353	306	274

gpr50 $^{-/y}$

Ob	155	234	210	262	200	330	221	248	248	141	108	102	159	214
C	115	59	34	48	50	64	36	54	17	124	183	31	115	23
S1	330	307	356	290	350	206	343	298	335	335	309	467	326	363

gpr50 $^{-/y}$ +mitoQ

Ob	239	239	204	114	218	211	220	163	224	234	245	207	186	243
C	57	54	159	68	84	86	80	68	18	78	25	48	46	66
S1	304	307	237	418	298	303	300	369	358	288	330	345	368	291

Figure 7C

Interaction time-Ob+S1

Gpr50 $^{+/y}$

Ob	79	98	56	82	161	33	61	76	49	43	45	57	62	53
S1	98	95	89	108	115	119	117	156	140	137	170	172	119	93

gpr50 $^{-/y}$

Ob	31	45	37	23	44	32	36	44	28	33	15	33	41	58
S1	75	107	69	119	90	85	73	83	91	88	76	107	108	119

gpr50 $^{-/y}$ +mitoQ

Ob	45	70	49	35	53	31	27	37	42	72	75	57	36	53
S1	184	126	103	137	121	99	67	136	108	151	217	130	147	160

Supplementary Figure 3B

Time in chamber-Ob+S1

Gpr50+/y

Ob	348	197	221	254	271	263	252	227	369	225	153	311	185	239	206
C	77	71	61	47	94	44	47	3	22	15	157	136	58	46	14
S1	175	332	318	299	235	293	301	370	209	360	290	153	357	315	380

Gpr50-/y

Ob	277	229	224	211	200	320	252	222	221	237	260	203	337	194	215	225
C	49	132	45	123	83	10	40	83	90	10	74	97	82	57	175	168
S1	274	239	331	266	317	270	308	295	289	353	266	300	181	349	210	207

Supplementary Figure 3C

Interaction time-Ob+S1

Gpr50+/y

Ob	97	35	44	61	68	32	29	38	73	56	43	48	30	46	32
S1	43	91	62	66	112	83	73	92	23	135	128	72	107	88	86

Gpr50-/y

Ob	23	16	33	30	23	33	40	71	60	67	31	90	44	43	54	30
S1	102	87	79	74	72	91	99	52	76	93	72	73	32	75	78	73

Supplementary Figure 3D

Time in chamber-F1+S2

Gpr50+/y

F1	263	194	215	191	157	200	226	238	222	208	271	187	269	239	198
C	73	121	72	136	75	130	97	16	93	97	39	209	109	101	116
S2	264	285	313	273	368	270	277	373	285	295	290	204	222	260	286

Gpr50-/y

F1	198	252	276	239	134	260	250	230	222	220	224	206	222	337	243	256
C	57	106	101	75	92	137	109	155	120	104	133	158	157	71	89	97
S2	345	242	223	286	374	203	241	215	258	276	243	236	221	192	268	247

Supplementary Figure 3E

Interaction time-F1+S2

Gpr50+/y

F1	24	22	73	30	10	22	26	55	60	36	32	24	28	18	25
S2	19	39	115	36	54	28	85	89	132	89	108	23	72	65	90

Gpr50-/y

F1	87	32	41	35	28	20	26	35	44	27	41	41	46	40	44	21
S2	56	15	41	21	71	33	18	4	56	28	51	33	45	16	21	36

mouse information and statistics

Figure 7B

Three chamber tests-time in chamber-Ob and S1

Group	Number of sample	Statistical test	One-tailed or two-tailed	*P* value	Significance	Summary	Degrees of freedom	T value	F value
Gpr50 $^{+/y}$	15 mice (15 male)	One-way ANOVA followed by LSD post hoc tests	One-tailed	0.024 (Ob vs S1)	Yes	*	5	n/a	$F_{5, 87}$ = 57.586
gpr50 $^{-/y}$	16 mice (16 male)			0.038 (Ob vs S1)	Yes	*			

Figure 7C

Three chamber tests-Interaction time-Ob and S1

Group	Number of sample	Statistical test	One-tailed or two-tailed	*P* value	Significance	Summary	Degrees of freedom	T value	F value
Gpr50 $^{+/y}$	15 mice (15 male)	Unpaired Student's t-test	Two-tailed	0.0006 (Ob vs S1)	Yes	***	28	-3.85	n/a
gpr50 $^{-/y}$	16 mice (16 male)	Unpaired Student's t-test	Two-tailed	0.000 (Ob vs S1)	Yes	***	30	-5.076	

Figure 7D

Three chamber tests-time in chamber-F1 and S2

Group	Number of sample	Statistical test	One-tailed or two-tailed	*P* value	Significance	Summary	Degrees of freedom	T value	F value
Gpr50 $^{+/y}$	15 mice (15 male)	One-way ANOVA followed by LSD post hoc tests	One-tailed	0.000 (F1 vs S2)	Yes	***	5	n/a	$F_{5, 87}$ = 54.627
gpr50 $^{-/y}$	16 mice (16 male)			0.199 (F1 vs S2)	No	ns			

Figure 7E

Three chamber tests-Interaction time-F1 and S2

Group	Number of sample	Statistical test	One-tailed or two-tailed	*P* value	Significance	Summary	Degrees of freedom	T value	F value
Gpr50 $^{+/y}$	15 mice (15 male)	Unpaired Student's t-test	Two-tailed	0.002 (F1 vs S2)	Yes	**	20.205	-3.661	n/a
gpr50 $^{-/y}$	16 mice (16 male)	Unpaired Student's t-test	Two-tailed	0.516 (F1 vs S2)	No	ns	30	0.657	

Buried food test

Figure 7F

Group	Number of sample	Statistical test	One-tailed or two-tailed	*P* value	Significance	Summary	Degrees of freedom	T value	F value
Gpr50 $^{+/y}$	15 mice (15 male)	Unpaired Student's t-test	Two-tailed	0.805 (*Gpr50* $^{+/y}$ vs *gpr50* $^{-/y}$)	No	ns	28	-0.249	n/a
gpr50 $^{-/y}$	15 mice (15 male)								

Figure 5J

OCR-cortex

Group	Number of sample	Statistical test	One-tailed or two-tailed	*P* value	Significance	Summary	Degrees of freedom	T value	F value
Leak	*Gpr50* $^{+/y}$: 6 mice (male); *gpr50* $^{-/y}$: 6 mice (male)	Unpaired Student's t-test	Two-tailed	0.000 (*Gpr50* $^{+/y}$ vs *gpr50* $^{-/y}$)	Yes	***	5	10.684	n/a
CI		Unpaired Student's t-test	Two-tailed	0.000 (*Gpr50* $^{+/y}$ vs *gpr50* $^{-/y}$)	Yes	***	5	8.351	n/a
CI & CII		Unpaired Student's t-test	Two-tailed	0.000 (*Gpr50* $^{+/y}$ vs *gpr50* $^{-/y}$)	Yes	***	5	17.792	n/a
CII		Unpaired Student's t-test	Two-tailed	0.032 (*Gpr50* $^{+/y}$ vs *gpr50* $^{-/y}$)	Yes	*	5	2.937	n/a
CIV		Unpaired Student's t-test	Two-tailed	0.000 (*Gpr50* $^{+/y}$ vs *gpr50* $^{-/y}$)	Yes	***	5	11.43	n/a

OCR-hippocampus

Group	Number of sample	Statistical test	One-tailed or two-tailed	*P* value	Significance	Summary	Degrees of freedom	T value	F value
Leak	*Gpr50* $^{+/y}$: 6 mice (male); *gpr50* $^{-/y}$: 6 mice (male)	Unpaired Student's t-test	Two-tailed	0.005(*Gpr50* $^{+/y}$ vs *gpr50* $^{-/y}$)	Yes	**	5	4.733	n/a
CI		Unpaired Student's t-test	Two-tailed	0.013 (*Gpr50* $^{+/y}$ vs *gpr50* $^{-/y}$)	Yes	*	5	3.774	n/a
CI & CII		Unpaired Student's t-test	Two-tailed	0.056 (*Gpr50* $^{+/y}$ vs *gpr50* $^{-/y}$)	No	ns	5	2.471	n/a
CII		Unpaired Student's t-test	Two-tailed	0.018 (*Gpr50* $^{+/y}$ vs *gpr50* $^{-/y}$)	Yes	*	5	3.482	n/a
CIV		Unpaired Student's t-test	Two-tailed	0.001 (*Gpr50* $^{+/y}$ vs *gpr50* $^{-/y}$)	Yes	*	5	6.329	n/a

Figure 5K

The max ETS capacity

Group	Number of sample	Statistical test	One-tailed or two-tailed	*P* value	Significance	Summary	Degrees of freedom	T value	F value
cortex	*Gpr50* $^{+/y}$: 6 mice (male); *gpr50* $^{-/y}$: 6 mice (male)	Unpaired Student's t-test	Two-tailed	0.000 (*Gpr50* $^{+/y}$ vs *gpr50* $^{-/y}$)	Yes	***	5	9.659	n/a
hippocampus		Unpaired Student's t-test	Two-tailed	0.018(*Gpr50* $^{+/y}$ vs *gpr50* $^{-/y}$)	Yes	*	5	3.461	n/a

Figure 5L

The spare respiratory capacity

Group	Number of sample	Statistical test	One-tailed or two-tailed	*P* value	Significance	Summary	Degrees of freedom	T value	F value
cortex	*Gpr50* $^{+/y}$: 5 mice (male); *gpr50* $^{-/y}$: 5 mice (male)	Unpaired Student's t-test	Two-tailed	0.001 (*Gpr50* $^{+/y}$ vs *gpr50* $^{-/y}$)	Yes	**	8	4.91	n/a
hippocampus		Unpaired Student's t-test	Two-tailed	0.028(*Gpr50* $^{+/y}$ vs *gpr50* $^{-/y}$)	Yes	*	4	3.357	n/a

Figure 5G

western blot of TOMM20

Group	Number of sample	Statistical test	One-tailed or two-tailed	*P* value	Significance	Summary	Degrees of freedom	T value	F value
cortex	*Gpr50* $^{+/y}$: 3 mice (male); *gpr50* $^{-/y}$: 3 mice (male)	Unpaired Student's t-test	Two-tailed	0.022 (*Gpr50* $^{+/y}$ vs *gpr50* $^{-/y}$)	Yes	*	4	-3.626	n/a
hippocampus	*Gpr50* $^{+/y}$: 3 mice (male); *gpr50* $^{-/y}$: 3 mice (male)	Unpaired Student's t-test	Two-tailed	0.002 (*Gpr50* $^{+/y}$ vs *gpr50* $^{-/y}$)	Yes	**	4	-7.007	n/a

Figure 8B

Three chamber tests-time in chamber-Ob and S1

Group	Number of sample	Statistical test	One-tailed or two-tailed	*P* value	Significance	Summary	Degrees of freedom	T value	F value
Gpr50 $^{+/y}$	14 mice (14 male)	One-way ANOVA followed by LSD post hoc	One-tailed	0.000 (Ob vs S1)	Yes	***	8	n/a	$F_{8, 117}$ = 68.037
gpr50 $^{-/y}$	14 mice (14 male)			0.000 (Ob vs S1)	Yes	***			
gpr50 $^{-/y}$ + mitoQ	14 mice (14 male)			0.000 (Ob vs S1)	Yes	***			

Figure 8C

Three chamber tests-Interaction time-Ob and S1

Group	Number of sample	Statistical test	One-tailed or two-tailed	*P* value	Significance	Summary	Degrees of freedom	T value	F value
Gpr50 $^{+/y}$	14 mice (14 male)	One-way ANOVA followed by Dunnett T3	One-tailed	0.000 (Ob vs S1)	Yes	***	5	n/a	$F_{5, 78}$ = 32.58
gpr50 $^{-/y}$	14 mice (14 male)			0.000 (Ob vs S1)	Yes	***			
gpr50 $^{-/y}$ + mitoQ	14 mice (14 male)			0.000 (Ob vs S1)	Yes	***			

Figure 8D

Three chamber tests-time in chamber-F1 and S2

Group	Number of sample	Statistical test	One-tailed or two-tailed	*P* value	Significance	Summary	Degrees of freedom	T value	F value
Gpr50 $^{+/y}$	14 mice (14 male)	One-way ANOVA followed by LSD post hoc	One-tailed	0.000 (Ob vs S1)	Yes	***	8	n/a	$F_{8, 123}$ = 70.486
gpr50 $^{-/y}$	14 mice (14 male)			0.226 (Ob vs S1)	No	ns			
gpr50 $^{-/y}$ + mitoQ	14 mice (14 male)			0.005 (Ob vs S1)	Yes	**			

Figure 8E

Three chamber tests-Interaction time-F1 and S2

Group	Number of sample	Statistical test	One-tailed or two-tailed	*P* value	Significance	Summary	Degrees of freedom	T value	F value
Gpr50 $^{+/y}$	14 mice (14 male)	One-way ANOVA followed by LSD post hoc	One-tailed	0.014 (Ob vs S1)	Yes	*	5	n/a	$F_{5, 78}$ = 5.273
gpr50 $^{-/y}$	14 mice (14 male)			0.61 (Ob vs S1)	No	ns			
gpr50 $^{-/y}$ + mitoQ	14 mice (14 male)			0.000 (Ob vs S1)	Yes	***			

GG/Access	PSMs/GG	PSMs/GG	PSMs/GG	SYMBOL	AVERAGE	AVERAGE+	LOG(A+1)
Q9Y6Y8	8	7	7	SEC23IP	7.333333	8.333333	3.058894
Q9Y6N7	5	5	4	ROBO1	4.666667	5.666667	2.5025
Q9Y6N1	3	3	4	COX11	3.333333	4.333333	2.115477
Q9Y6M1	13	13	13	IGF2BP2	13	14	3.807355
Q9Y6K0	4	3	5	CEPT1	4	5	2.321928
Q9Y6J9	1	1	1	TAF6L	1	2	1
Q9Y6I9	9	8	7	TEX264	8	9	3.169925
Q9Y6E2	4	3	4	BZW2	3.666667	4.666667	2.222392
Q9Y697	1	2	3	NFS1	2	3	1.584963
Q9Y679	21	24	21	AUP1	22	23	4.523562
Q9Y678	5	4	4	COPG1	4.333333	5.333333	2.415037
Q9Y676	7	7	6	MRPS18B	6.666667	7.666667	2.938599
Q9Y613	12	10	10	FHOD1	10.66667	11.66667	3.544321
Q9Y5Y2	4	4	4	NUBP2	4	5	2.321928
Q9Y5X3	3	2	3	SNX5	2.666667	3.666667	1.874469
Q9Y5V3	12	20	18	MAGED1	16.66667	17.66667	4.142958
Q9Y5T5	7	5	6	USP16	6	7	2.807355
Q9Y5Q9	7	7	7	GTF3C3	7	8	3
Q9Y5Q8	9	7	8	GTF3C5	8	9	3.169925
Q9Y5P6	2	1	2	GMPPB	1.666667	2.666667	1.415037
Q9Y5M8	15	14	13	SRPRB	14	15	3.906891
Q9Y5L0	6	7	7	TNPO3	6.666667	7.666667	2.938599
Q9Y5B6	2	1	1	PAXBP1	1.333333	2.333333	1.222392
Q9Y5A9	20	19	22	YTHDF2	20.33333	21.33333	4.415037
Q9Y520	13	14	11	PRRC2C	12.66667	13.66667	3.77259
Q9Y4Z0	4	5	4	LSM4	4.333333	5.333333	2.415037
Q9Y4X5	3	5	2	ARIH1	3.333333	4.333333	2.115477
Q9Y4W6	19	16	18	AFG3L2	17.66667	18.66667	4.222392
Q9Y4W2	7	7	8	LAS1L	7.333333	8.333333	3.058894
Q9Y4R8	33	29	33	TELO2	31.66667	32.66667	5.029747
Q9Y4P3	40	38	42	TBL2	40	41	5.357552
Q9Y4C2	3	4	8	TCAF1	5	6	2.584963
Q9Y4B6	34	31	39	DCAF1	34.66667	35.66667	5.156504
Q9Y478	1	2	2	PRKAB1	1.666667	2.666667	1.415037
Q9Y450	3	4	1	HBS1L	2.666667	3.666667	1.874469
Q9Y3Z3	13	15	13	SAMHD1	13.66667	14.66667	3.874469
Q9Y3Y2	8	7	7	CHTOP	7.333333	8.333333	3.058894
Q9Y3U8	22	21	15	RPL36	19.33333	20.33333	4.345775
Q9Y3R5	1	1	1	DOP1B	1	2	1
Q9Y3L5	6	3	1	RAP2C	3.333333	4.333333	2.115477
Q9Y3I0	22	25	23	RTCB	23.33333	24.33333	4.604862
Q9Y3F4	17	18	18	STRAP	17.66667	18.66667	4.222392
Q9Y3E5	12	14	13	PTRH2	13	14	3.807355
Q9Y3D9	6	7	5	MRPS23	6	7	2.807355
Q9Y3D8	3	2	3	AK6	2.666667	3.666667	1.874469
Q9Y3D6	2	1	2	FIS1	1.666667	2.666667	1.415037
Q9Y3D5	2	3	5	MRPS18C	3.333333	4.333333	2.115477
Q9Y3D3	7	9	9	MRPS16	8.333333	9.333333	3.222392
Q9Y3D0	5	6	7	CIAO2B	6	7	2.807355
Q9Y3C8	1	2	1	UFC1	1.333333	2.333333	1.222392
Q9Y3C6	3	2	3	PPIL1	2.666667	3.666667	1.874469
Q9Y3C1	9	7	4	NOP16	6.666667	7.666667	2.938599
Q9Y3B2	5	1	5	EXOSC1	3.666667	4.666667	2.222392
Q9Y399	13	13	13	MRPS2	13	14	3.807355
Q9Y383	29	30	28	LUC7L2	29	30	4.906891

Q9Y314	4	5	6	NOSIP	5	6	2.584963
Q9Y312	7	4	4	AAR2	5	6	2.584963
Q9Y305	2	1	1	ACOT9	1.333333	2.333333	1.222392
Q9Y2X3	17	21	17	NOP58	18.33333	19.33333	4.273018
Q9Y2U8	2	4	1	LEMD3	2.333333	3.333333	1.736966
Q9Y2T2	3	4	4	AP3M1	3.666667	4.666667	2.222392
Q9Y2R9	17	19	19	MRPS7	18.33333	19.33333	4.273018
Q9Y2R5	5	7	5	MRPS17	5.666667	6.666667	2.736966
Q9Y2R4	11	10	11	DDX52	10.66667	11.66667	3.544321
Q9Y2Q9	8	6	7	MRPS28	7	8	3
Q9Y2L1	7	6	5	DIS3	6	7	2.807355
Q9Y2I1	4	4	3	NISCH	3.666667	4.666667	2.222392
Q9Y2H6	2	4	3	FNDC3A	3	4	2
Q9Y2G8	5	4	8	DNAJC16	5.666667	6.666667	2.736966
Q9Y295	24	20	20	DRG1	21.33333	22.33333	4.481127
Q9Y289	3	3	3	SLC5A6	3	4	2
Q9Y287	5	5	3	ITM2B	4.333333	5.333333	2.415037
Q9Y285	12	17	13	FARSA	14	15	3.906891
Q9Y265	24	25	23	RUVBL1	24	25	4.643856
Q9Y256	2	3	3	RCE1	2.666667	3.666667	1.874469
Q9Y241	4	1	2	HIGD1A	2.333333	3.333333	1.736966
Q9Y230	20	16	16	RUVBL2	17.33333	18.33333	4.196397
Q9Y224	22	20	20	RTRAF	20.66667	21.66667	4.437405
Q9Y221	6	6	4	NIP7	5.333333	6.333333	2.662965
Q9UQ35	1	1	2	SRRM2	1.333333	2.333333	1.222392
Q9UPY5	1	2	2	SLC7A11	1.666667	2.666667	1.415037
Q9UPY3	19	22	24	DICER1	21.66667	22.66667	4.5025
Q9UPU5	7	6	11	USP24	8	9	3.169925
Q9UPT8	4	5	6	ZC3H4	5	6	2.584963
Q9UPT5	3	2	6	EXOC7	3.666667	4.666667	2.222392
Q9UPP1	4	6	4	PHF8	4.666667	5.666667	2.5025
Q9UPN7	23	23	25	PPP6R1	23.66667	24.66667	4.624491
Q9UNX4	15	20	15	WDR3	16.66667	17.66667	4.142958
Q9UNQ2	16	15	17	DIMT1	16	17	4.087463
Q9UNM6	5	7	7	PSMD13	6.333333	7.333333	2.874469
Q9UNL2	7	6	6	SSR3	6.333333	7.333333	2.874469
Q9UNK9	2	3	3	ANGEL1	2.666667	3.666667	1.874469
Q9UNF1	17	21	18	MAGED2	18.66667	19.66667	4.297681
Q9UN86	21	16	17	G3BP2	18	19	4.247928
Q9UN37	1	3	4	VPS4A	2.666667	3.666667	1.874469
Q9UMS4	34	33	34	PRPF19	33.66667	34.66667	5.115477
Q9UM00	5	5	6	TMCO1	5.333333	6.333333	2.662965
Q9ULX6	10	12	12	AKAP8L	11.33333	12.33333	3.624491
Q9ULX3	7	8	7	NOB1	7.333333	8.333333	3.058894
Q9ULW3	3	2	3	ABT1	2.666667	3.666667	1.874469
Q9ULW0	7	5	4	TPX2	5.333333	6.333333	2.662965
Q9ULJ3	2	2	2	ZBTB21	2	3	1.584963
Q9ULC4	2	5	4	MCTS1	3.666667	4.666667	2.222392
Q9ULA0	1	3	2	DNPEP	2	3	1.584963
Q9UL54	7	4	4	TAOK2	5	6	2.584963
Q9UL25	3	3	4	RAB21	3.333333	4.333333	2.115477
Q9UKV5	4	4	5	AMFR	4.333333	5.333333	2.415037
Q9UKN8	11	7	14	GTF3C4	10.66667	11.66667	3.544321
Q9UKM7	2	3	2	MAN1B1	2.333333	3.333333	1.736966
Q9UKF6	4	6	7	CPSF3	5.666667	6.666667	2.736966
Q9UKA4	4	4	3	AKAP11	3.666667	4.666667	2.222392

Q9UK41	1	4	3	VPS28	2.666667	3.666667	1.874469
Q9UJZ1	4	3	5	STOML2	4	5	2.321928
Q9UJV9	21	21	23	DDX41	21.66667	22.66667	4.5025
Q9UJS0	20	25	24	SLC25A13	23	24	4.584963
Q9UJ14	4	2	2	GGT7	2.666667	3.666667	1.874469
Q9UII4	22	23	23	HERC5	22.66667	23.66667	4.564785
Q9UIG0	2	3	4	BAZ1B	3	4	2
Q9UIA9	1	2	3	XPO7	2	3	1.584963
Q9UI30	5	4	7	TRMT112	5.333333	6.333333	2.662965
Q9UI26	7	10	8	IPO11	8.333333	9.333333	3.222392
Q9UI12	3	4	4	ATP6V1H	3.666667	4.666667	2.222392
Q9UI10	28	23	26	EIF2B4	25.66667	26.66667	4.736966
Q9UHX1	15	14	15	PUF60	14.66667	15.66667	3.969626
Q9UHW5	1	1	1	GPN3	1	2	1
Q9UHV9	1	2	1	PFDN2	1.333333	2.333333	1.222392
Q9UHI8	4	3	3	ADAMTS1	3.333333	4.333333	2.115477
Q9UHI6	30	30	35	DDX20	31.66667	32.66667	5.029747
Q9UHB9	18	15	16	SRP68	16.33333	17.33333	4.115477
Q9UH99	3	5	4	SUN2	4	5	2.321928
Q9UH62	10	10	8	ARMCX3	9.333333	10.33333	3.369234
Q9UGR2	9	13	16	ZC3H7B	12.66667	13.66667	3.77259
Q9UGP8	24	29	28	SEC63	27	28	4.807355
Q9UG63	36	38	39	ABCF2	37.66667	38.66667	5.273018
Q9UG56	3	5	4	PISD	4	5	2.321928
Q9UFG5	2	2	2	C19orf25	2	3	1.584963
Q9UET6	6	6	8	FTSJ1	6.666667	7.666667	2.938599
Q9UEG4	10	8	6	ZNF629	8	9	3.169925
Q9UDY4	3	3	3	DNAJB4	3	4	2
Q9UDW1	2	1	2	UQCR10	1.666667	2.666667	1.415037
Q9UBX3	7	10	12	SLC25A10	9.666667	10.66667	3.415037
Q9UBV8	3	4	2	PEF1	3	4	2
Q9UBV7	1	4	3	B4GALT7	2.666667	3.666667	1.874469
Q9UBU6	1	1	1	FAM8A1	1	2	1
Q9UBS4	3	4	4	DNAJB11	3.666667	4.666667	2.222392
Q9UBN7	2	3	1	HDAC6	2	3	1.584963
Q9UBM7	11	13	11	DHCR7	11.66667	12.66667	3.662965
Q9UBF2	6	6	7	COPG2	6.333333	7.333333	2.874469
Q9UBD5	3	4	3	ORC3	3.333333	4.333333	2.115477
Q9UBB9	6	4	7	TFIP11	5.666667	6.666667	2.736966
Q9UBB6	3	2	4	NCDN	3	4	2
Q9UBB4	17	12	15	ATXN10	14.66667	15.66667	3.969626
Q9P2N5	9	10	12	RBM27	10.33333	11.33333	3.5025
Q9P2J5	6	6	6	LARS1	6	7	2.807355
Q9P2I0	4	5	2	CPSF2	3.666667	4.666667	2.222392
Q9P2E9	40	47	42	RRBP1	43	44	5.459432
Q9P2E5	3	1	4	CHPF2	2.666667	3.666667	1.874469
Q9P287	1	1	1	BCCIP	1	2	1
Q9P258	33	36	31	RCC2	33.33333	34.33333	5.101538
Q9P1F3	1	1	1	ABRACL	1	2	1
Q9P0L0	5	5	4	VAPA	4.666667	5.666667	2.5025
Q9P0J0	6	5	5	NDUFA13	5.333333	6.333333	2.662965
Q9P035	49	49	46	HACD3	48	49	5.61471
Q9P032	5	7	4	NDUFAF4	5.333333	6.333333	2.662965
Q9P015	8	7	5	MRPL15	6.666667	7.666667	2.938599
Q9P003	6	5	7	CNIH4	6	7	2.807355
Q9NZL9	1	1	2	MAT2B	1.333333	2.333333	1.222392

Q9NZI8	49	53	52	IGF2BP1	51.33333	52.33333	5.709658
Q9NZB2	4	4	5	FAM120A	4.333333	5.333333	2.415037
Q9NZ01	17	16	19	TECR	17.33333	18.33333	4.196397
Q9NYU2	1	1	2	UGGT1	1.333333	2.333333	1.222392
Q9NYR9	1	1	1	NKIRAS2	1	2	1
Q9NYP7	1	1	1	ELOVL5	1	2	1
Q9NYK5	5	6	5	MRPL39	5.333333	6.333333	2.662965
Q9NYF8	4	7	8	BCLAF1	6.333333	7.333333	2.874469
Q9NY12	3	8	6	GAR1	5.666667	6.666667	2.736966
Q9NXW2	4	2	5	DNAJB12	3.666667	4.666667	2.222392
Q9NXR7	1	2	2	BABAM2	1.666667	2.666667	1.415037
Q9NXF7	4	3	3	DCAF16	3.333333	4.333333	2.115477
Q9NXF1	8	9	8	TEX10	8.333333	9.333333	3.222392
Q9NXE4	1	3	5	SMPD4	3	4	2
Q9NX61	9	12	12	TMEM161A	11	12	3.584963
Q9NX58	18	14	12	LYAR	14.66667	15.66667	3.969626
Q9NX24	3	4	5	NHP2	4	5	2.321928
Q9NX20	6	6	7	MRPL16	6.333333	7.333333	2.874469
Q9NWW5	3	4	2	CLN6	3	4	2
Q9NW64	2	2	3	RBM22	2.333333	3.333333	1.736966
Q9NW13	19	15	17	RBM28	17	18	4.169925
Q9NW08	1	1	2	POLR3B	1.333333	2.333333	1.222392
Q9NVP1	32	29	30	DDX18	30.33333	31.33333	4.969626
Q9NVN8	21	19	19	GNL3L	19.66667	20.66667	4.369234
Q9NVJ2	1	2	1	ARL8B	1.333333	2.333333	1.222392
Q9NVI7	30	31	33	ATAD3A	31.33333	32.33333	5.01495
Q9NVI1	21	20	19	FANCI	20	21	4.392317
Q9NVH0	9	9	8	EXD2	8.666667	9.666667	3.273018
Q9NV70	4	3	2	EXOC1	3	4	2
Q9NUQ6	4	4	2	SPATS2L	3.333333	4.333333	2.115477
Q9NUD5	6	7	5	ZCCHC3	6	7	2.807355
Q9NU22	44	49	52	MDN1	48.33333	49.33333	5.624491
Q9NTJ3	27	31	21	SMC4	26.33333	27.33333	4.77259
Q9NTI5	5	6	9	PDS5B	6.666667	7.666667	2.938599
Q9NSE4	3	1	2	IARS2	2	3	1.584963
Q9NSD9	11	11	12	FARSB	11.33333	12.33333	3.624491
Q9NS86	10	8	8	LANCL2	8.666667	9.666667	3.273018
Q9NS69	2	2	2	TOMM22	2	3	1.584963
Q9NRZ9	14	14	15	HELLS	14.33333	15.33333	3.938599
Q9NRZ5	5	3	5	AGPAT4	4.333333	5.333333	2.415037
Q9NRX2	4	3	2	MRPL17	3	4	2
Q9NRW3	6	7	9	APOBEC3C	7.333333	8.333333	3.058894
Q9NRR4	2	1	1	DROSHA	1.333333	2.333333	1.222392
Q9NRL2	3	2	7	BAZ1A	4	5	2.321928
Q9NRG9	3	4	4	AAAS	3.666667	4.666667	2.222392
Q9NR77	4	2	3	PXMP2	3	4	2
Q9NR50	22	29	22	EIF2B3	24.33333	25.33333	4.662965
Q9NR30	79	76	74	DDX21	76.33333	77.33333	6.273018
Q9NR09	1	1	2	BIRC6	1.333333	2.333333	1.222392
Q9NQX7	3	6	5	ITM2C	4.666667	5.666667	2.5025
Q9NQT5	4	6	9	EXOSC3	6.333333	7.333333	2.874469
Q9NQT4	5	7	3	EXOSC5	5	6	2.584963
Q9NQC3	2	3	4	RTN4	3	4	2
Q9NQ55	8	10	10	PPAN	9.333333	10.33333	3.369234
Q9NQ29	14	16	16	LUC7L	15.33333	16.33333	4.029747
Q9NPQ8	4	2	4	RIC8A	3.333333	4.333333	2.115477

Q9NPI6	1	1	2	DCP1A	1.333333	2.333333	1.222392
Q9NPE3	1	1	2	NOP10	1.333333	2.333333	1.222392
Q9NPD3	4	3	5	EXOSC4	4	5	2.321928
Q9NP81	6	7	9	SARS2	7.333333	8.333333	3.058894
Q9NP72	5	6	7	RAB18	6	7	2.807355
Q9NNW5	26	25	23	WDR6	24.66667	25.66667	4.681824
Q9HDC9	2	1	2	APMAP	1.666667	2.666667	1.415037
Q9HD45	6	8	6	TM9SF3	6.666667	7.666667	2.938599
Q9HD33	4	5	5	MRPL47	4.666667	5.666667	2.5025
Q9HCU5	18	19	18	PREB	18.33333	19.33333	4.273018
Q9HCM4	11	10	10	EPB41L5	10.33333	11.33333	3.5025
Q9HCG8	4	4	3	CWC22	3.666667	4.666667	2.222392
Q9HCE1	16	15	14	MOV10	15	16	4
Q9HCC0	5	6	8	MCCC2	6.333333	7.333333	2.874469
Q9HC36	16	18	18	MRM3	17.33333	18.33333	4.196397
Q9HC21	3	1	3	SLC25A19	2.333333	3.333333	1.736966
Q9HC07	4	5	5	TMEM165	4.666667	5.666667	2.5025
Q9HBE1	3	5	5	PATZ1	4.333333	5.333333	2.415037
Q9HB71	2	3	2	CACYBP	2.333333	3.333333	1.736966
Q9HAV4	35	32	35	XPO5	34	35	5.129283
Q9HAU5	5	3	4	UPF2	4	5	2.321928
Q9HA64	12	15	15	FN3KRP	14	15	3.906891
Q9H9S3	10	15	10	SEC61A2	11.66667	12.66667	3.662965
Q9H9J2	7	7	9	MRPL44	7.666667	8.666667	3.115477
Q9H9B4	5	5	4	SFXN1	4.666667	5.666667	2.5025
Q9H9A6	3	3	3	LRRC40	3	4	2
Q9H936	8	13	10	SLC25A22	10.33333	11.33333	3.5025
Q9H8V3	11	9	11	ECT2	10.33333	11.33333	3.5025
Q9H8H2	12	9	10	DDX31	10.33333	11.33333	3.5025
Q9H857	8	6	6	NT5DC2	6.666667	7.666667	2.938599
Q9H7E9	14	12	13	C8orf33	13	14	3.807355
Q9H7D7	1	3	1	WDR26	1.666667	2.666667	1.415037
Q9H7B2	7	5	4	RPF2	5.333333	6.333333	2.662965
Q9H773	3	3	3	DCTPP1	3	4	2
Q9H6S0	58	57	65	YTHDC2	60	61	5.930737
Q9H6R4	3	6	5	NOL6	4.666667	5.666667	2.5025
Q9H6H4	4	4	5	REEP4	4.333333	5.333333	2.415037
Q9H5Q4	3	3	4	TFB2M	3.333333	4.333333	2.115477
Q9H5K3	1	2	2	POMK	1.666667	2.666667	1.415037
Q9H5H4	4	1	4	ZNF768	3	4	2
Q9H583	18	21	23	HEATR1	20.66667	21.66667	4.437405
Q9H501	6	6	6	ESF1	6	7	2.807355
Q9H4A6	5	7	6	GOLPH3	6	7	2.807355
Q9H490	2	2	2	PIGU	2	3	1.584963
Q9H3U1	5	9	8	UNC45A	7.333333	8.333333	3.058894
Q9H3P7	5	5	3	ACBD3	4.333333	5.333333	2.415037
Q9H3N1	5	5	6	TMX1	5.333333	6.333333	2.662965
Q9H3K6	12	12	13	BOLA2	12.33333	13.33333	3.736966
Q9H2V7	3	2	2	SPNS1	2.333333	3.333333	1.736966
Q9H2U1	30	32	30	DHX36	30.66667	31.66667	4.984893
Q9H2J7	4	2	4	SLC6A15	3.333333	4.333333	2.115477
Q9H2H9	3	3	3	SLC38A1	3	4	2
Q9H2G4	1	1	1	TSPYL2	1	2	1
Q9H1I8	9	9	10	ASCC2	9.333333	10.33333	3.369234
Q9H1E5	2	1	2	TMX4	1.666667	2.666667	1.415037
Q9H1A4	6	7	6	ANAPC1	6.333333	7.333333	2.874469

Q9H0U9	7	4	7	TSPYL1	6	7	2.807355
Q9H0U3	20	19	18	MAGT1	19	20	4.321928
Q9H0S4	14	11	12	DDX47	12.33333	13.33333	3.736966
Q9H0H5	5	9	9	RACGAP1	7.666667	8.666667	3.115477
Q9H0D6	36	31	29	XRN2	32	33	5.044394
Q9H0C8	2	4	2	ILKAP	2.666667	3.666667	1.874469
Q9H0A0	38	46	41	NAT10	41.66667	42.66667	5.415037
Q9H078	11	8	6	CLPB	8.333333	9.333333	3.222392
Q9H061	5	5	5	TMEM126/	5	6	2.584963
Q9H000	3	1	2	MKRN2	2	3	1.584963
Q9GZS3	6	5	5	SKIC8	5.333333	6.333333	2.662965
Q9GZR7	24	24	25	DDX24	24.33333	25.33333	4.662965
Q9GZQ8	1	1	2	MAP1LC3B	1.333333	2.333333	1.222392
Q9C0J8	3	2	1	WDR33	2	3	1.584963
Q9C0E8	3	3	4	LNPK	3.333333	4.333333	2.115477
Q9C0C9	20	23	22	UBE2O	21.66667	22.66667	4.5025
Q9C0B1	4	4	3	FTO	3.666667	4.666667	2.222392
Q9C037	2	3	2	TRIM4	2.333333	3.333333	1.736966
Q9C005	3	2	4	DPY30	3	4	2
Q9BZX2	12	8	11	UCK2	10.33333	11.33333	3.5025
Q9BZI7	5	3	5	UPF3B	4.333333	5.333333	2.415037
Q9BZH6	19	15	17	WDR11	17	18	4.169925
Q9BZF1	7	7	11	OSBPL8	8.333333	9.333333	3.222392
Q9BZE4	26	34	28	GTPBP4	29.33333	30.33333	4.922832
Q9BZE1	3	4	4	MRPL37	3.666667	4.666667	2.222392
Q9BYN8	9	7	9	MRPS26	8.333333	9.333333	3.222392
Q9BYJ9	9	11	14	YTHDF1	11.33333	12.33333	3.624491
Q9BYD3	8	6	6	MRPL4	6.666667	7.666667	2.938599
Q9BYB4	2	1	3	GNB1L	2	3	1.584963
Q9BY77	2	2	1	POLDIP3	1.666667	2.666667	1.415037
Q9BY32	1	1	1	ITPA	1	2	1
Q9BXW9	27	27	32	FANCD2	28.66667	29.66667	4.890771
Q9BXW7	3	5	4	HDHD5	4	5	2.321928
Q9BXS4	2	5	4	TMEM59	3.666667	4.666667	2.222392
Q9BX40	2	2	3	LSM14B	2.333333	3.333333	1.736966
Q9BWF3	30	29	33	RBM4	30.66667	31.66667	4.984893
Q9BW92	19	20	17	TARS2	18.66667	19.66667	4.297681
Q9BW60	2	3	3	ELOVL1	2.666667	3.666667	1.874469
Q9BW27	4	3	5	NUP85	4	5	2.321928
Q9BW19	11	8	5	KIFC1	8	9	3.169925
Q9BVS4	7	9	6	RIOK2	7.333333	8.333333	3.058894
Q9BVQ7	8	9	11	SPATA5L1	9.333333	10.33333	3.369234
Q9BVP2	54	55	52	GNL3	53.66667	54.66667	5.77259
Q9BVK6	1	2	3	TMED9	2	3	1.584963
Q9BVI4	20	22	18	NOC4L	20	21	4.392317
Q9BVG9	2	2	3	PTDSS2	2.333333	3.333333	1.736966
Q9BV68	4	4	5	RNF126	4.333333	5.333333	2.415037
Q9BV44	3	3	6	THUMPD3	4	5	2.321928
Q9BV38	2	4	3	WDR18	3	4	2
Q9BUQ8	4	1	2	DDX23	2.333333	3.333333	1.736966
Q9BUN8	3	2	3	DERL1	2.666667	3.666667	1.874469
Q9BUJ2	30	33	34	HNRNPUL1	32.33333	33.33333	5.058894
Q9BUF5	78	72	70	TUBB6	73.33333	74.33333	6.215937
Q9BU76	7	7	6	C1orf35	6.666667	7.666667	2.938599
Q9BU23	6	8	7	LMF2	7	8	3
Q9BTY2	2	2	2	FUCA2	2	3	1.584963

Q9BTT6	7	9	9	LRRC1	8.333333	9.333333	3.222392
Q9BTE7	4	3	4	DCUN1D5	3.666667	4.666667	2.222392
Q9BTE3	1	1	1	MCMBP	1	2	1
Q9BTD8	7	8	8	RBM42	7.666667	8.666667	3.115477
Q9BTC0	10	11	10	DIDO1	10.33333	11.33333	3.5025
Q9BT40	7	4	9	INPP5K	6.666667	7.666667	2.938599
Q9BT22	8	8	10	ALG1	8.666667	9.666667	3.273018
Q9BSR8	1	1	1	YIPF4	1	2	1
Q9BSJ8	11	15	10	ESYT1	12	13	3.70044
Q9BSD7	10	11	13	NTPCR	11.33333	12.33333	3.624491
Q9BS26	1	1	2	ERP44	1.333333	2.333333	1.222392
Q9BRX2	11	13	10	PELO	11.33333	12.33333	3.624491
Q9BRP1	3	5	3	PDCD2L	3.666667	4.666667	2.222392
Q9BRJ7	5	5	6	NUDT16L1	5.333333	6.333333	2.662965
Q9BRJ6	7	6	9	C7orf50	7.333333	8.333333	3.058894
Q9BQG0	122	118	117	MYBBP1A	119	120	6.906891
Q9BQE3	125	124	121	TUBA1C	123.3333	124.3333	6.958069
Q9BQB6	3	3	3	VKORC1	3	4	2
Q9BQ75	20	24	21	CMSS1	21.66667	22.66667	4.5025
Q9BQ70	22	22	19	TCF25	21	22	4.459432
Q9BQ67	31	27	25	GRWD1	27.66667	28.66667	4.841302
Q9BQ52	3	2	1	ELAC2	2	3	1.584963
Q9BQ39	13	11	15	DDX50	13	14	3.807355
Q9BPX6	4	5	5	MICU1	4.666667	5.666667	2.5025
Q9BPX3	6	8	6	NCAPG	6.666667	7.666667	2.938599
Q99942	1	1	1	RNF5	1	2	1
Q99873	3	6	4	PRMT1	4.333333	5.333333	2.415037
Q99848	14	15	15	EBNA1BP2	14.66667	15.66667	3.969626
Q99832	6	8	6	CCT7	6.666667	7.666667	2.938599
Q99816	2	2	2	TSG101	2	3	1.584963
Q99805	1	1	1	TM9SF2	1	2	1
Q99755	5	5	6	PIP5K1A	5.333333	6.333333	2.662965
Q99742	7	6	3	NPAS1	5.333333	6.333333	2.662965
Q99733	19	18	19	NAP1L4	18.66667	19.66667	4.297681
Q99729	6	5	5	HNRNPAB	5.333333	6.333333	2.662965
Q99717	1	1	1	SMAD5	1	2	1
Q99714	3	3	6	HSD17B10	4	5	2.321928
Q99700	1	2	2	ATXN2	1.666667	2.666667	1.415037
Q99661	9	9	7	KIF2C	8.333333	9.333333	3.222392
Q99653	9	11	9	CHP1	9.666667	10.66667	3.415037
Q99623	1	2	4	PHB2	2.333333	3.333333	1.736966
Q99615	12	12	9	DNAJC7	11	12	3.584963
Q99598	1	3	1	TSNAX	1.666667	2.666667	1.415037
Q99575	14	11	12	POP1	12.33333	13.33333	3.736966
Q99497	12	13	12	PARK7	12.33333	13.33333	3.736966
Q99460	9	16	13	PSMD1	12.66667	13.66667	3.77259
Q99459	32	35	33	CDC5L	33.33333	34.33333	5.101538
Q99442	3	3	3	SEC62	3	4	2
Q96TA2	12	11	14	YME1L1	12.33333	13.33333	3.736966
Q96T76	57	57	59	MMS19	57.66667	58.66667	5.874469
Q96T37	2	3	2	RBM15	2.333333	3.333333	1.736966
Q96SB4	27	28	25	SRPK1	26.66667	27.66667	4.790077
Q96S52	2	2	4	PIGS	2.666667	3.666667	1.874469
Q96RT1	9	6	6	ERBIN	7	8	3
Q96QU8	6	4	7	XPO6	5.666667	6.666667	2.736966
Q96QE5	3	2	4	TEFM	3	4	2

Q96QC0	3	4	4	PPP1R10	3.666667	4.666667	2.222392
Q96PV6	5	5	9	LENG8	6.333333	7.333333	2.874469
Q96PU8	4	4	4	QKI	4	5	2.321928
Q96PK6	6	5	6	RBM14	5.666667	6.666667	2.736966
Q96P70	23	28	28	IPO9	26.33333	27.33333	4.77259
Q96P11	6	6	7	NSUN5	6.333333	7.333333	2.874469
Q96N67	2	3	4	DOCK7	3	4	2
Q96ME7	3	3	2	ZNF512	2.666667	3.666667	1.874469
Q96KR1	19	13	19	ZFR	17	18	4.169925
Q96KP1	6	3	7	EXOC2	5.333333	6.333333	2.662965
Q96KC8	5	4	4	DNAJC1	4.333333	5.333333	2.415037
Q96KA5	2	4	3	CLPTM1L	3	4	2
Q96K37	4	5	5	SLC35E1	4.666667	5.666667	2.5025
Q96JJ7	3	4	3	TMX3	3.333333	4.333333	2.115477
Q96JB2	4	1	4	COG3	3	4	2
Q96IX5	2	2	1	ATP5MK	1.666667	2.666667	1.415037
Q96I24	25	33	30	FUBP3	29.33333	30.33333	4.922832
Q96HS1	14	15	13	PGAM5	14	15	3.906891
Q96HR8	4	3	5	NAF1	4	5	2.321928
Q96HA7	4	3	4	TONSL	3.666667	4.666667	2.222392
Q96H79	16	11	12	ZC3HAV1L	13	14	3.807355
Q96GQ7	15	13	12	DDX27	13.33333	14.33333	3.841302
Q96GM8	7	7	7	TOE1	7	8	3
Q96GC9	1	4	3	VMP1	2.666667	3.666667	1.874469
Q96GA3	19	19	19	LTV1	19	20	4.321928
Q96G23	8	8	8	CERS2	8	9	3.169925
Q96FJ2	6	8	9	DYNLL2	7.666667	8.666667	3.115477
Q96F86	4	3	5	EDC3	4	5	2.321928
Q96EY7	30	31	32	PTCD3	31	32	5
Q96EY4	10	12	10	TMA16	10.66667	11.66667	3.544321
Q96EY1	15	15	16	DNAJA3	15.33333	16.33333	4.029747
Q96ES7	1	2	2	SGF29	1.666667	2.666667	1.415037
Q96ER3	15	13	15	SAAL1	14.33333	15.33333	3.938599
Q96EP5	1	3	2	DAZAP1	2	3	1.584963
Q96EL2	4	2	3	MRPS24	3	4	2
Q96EK4	3	3	3	THAP11	3	4	2
Q96E22	5	4	4	NUS1	4.333333	5.333333	2.415037
Q96DH6	8	8	15	MSI2	10.33333	11.33333	3.5025
Q96D53	9	11	8	COQ8B	9.333333	10.33333	3.369234
Q96D46	2	3	2	NMD3	2.333333	3.333333	1.736966
Q96D09	4	5	5	GPRASP2	4.666667	5.666667	2.5025
Q96D05	3	3	2	FAM241B	2.666667	3.666667	1.874469
Q96CW1	6	7	6	AP2M1	6.333333	7.333333	2.874469
Q96CS3	20	21	19	FAF2	20	21	4.392317
Q96CN7	6	5	5	ISOC1	5.333333	6.333333	2.662965
Q96CB9	3	2	2	NSUN4	2.333333	3.333333	1.736966
Q96C36	2	1	1	PYCR2	1.333333	2.333333	1.222392
Q96BN8	2	2	3	OTULIN	2.333333	3.333333	1.736966
Q96AY3	2	1	1	FKBP10	1.333333	2.333333	1.222392
Q96AG4	43	47	45	LRRC59	45	46	5.523562
Q96AB3	12	11	11	ISOC2	11.33333	12.33333	3.624491
Q96A65	7	11	7	EXOC4	8.333333	9.333333	3.222392
Q96A33	32	29	27	CCDC47	29.33333	30.33333	4.922832
Q969X5	3	2	2	ERGIC1	2.333333	3.333333	1.736966
Q969V3	17	21	16	NCLN	18	19	4.247928
Q969N2	2	1	2	PIGT	1.666667	2.666667	1.415037

Q969M3	5	4	6	YIPF5	5	6	2.584963
Q93077	21	19	18	H2AC6	19.33333	20.33333	4.345775
Q93063	1	1	1	EXT2	1	2	1
Q93009	17	21	21	USP7	19.66667	20.66667	4.369234
Q93008	25	22	28	USP9X	25	26	4.70044
Q92990	4	4	5	GLMN	4.333333	5.333333	2.415037
Q92974	11	10	10	ARHGEF2	10.33333	11.33333	3.5025
Q92973	19	22	21	TNPO1	20.66667	21.66667	4.437405
Q92947	6	9	8	GCDH	7.666667	8.666667	3.115477
Q92945	23	26	23	KHSRP	24	25	4.643856
Q92922	6	6	9	SMARCC1	7	8	3
Q92900	13	15	10	UPF1	12.66667	13.66667	3.77259
Q92896	7	4	7	GLG1	6	7	2.807355
Q92841	83	82	84	DDX17	83	84	6.392317
Q92804	2	3	4	TAF15	3	4	2
Q92796	3	2	4	DLG3	3	4	2
Q92769	9	8	9	HDAC2	8.666667	9.666667	3.273018
Q92759	2	2	3	GTF2H4	2.333333	3.333333	1.736966
Q92667	13	17	13	AKAP1	14.33333	15.33333	3.938599
Q92665	16	16	17	MRPS31	16.33333	17.33333	4.115477
Q92621	17	18	12	NUP205	15.66667	16.66667	4.058894
Q92620	5	7	8	DHX38	6.666667	7.666667	2.938599
Q92616	96	87	90	GCN1	91	92	6.523562
Q92615	8	10	11	LARP4B	9.666667	10.66667	3.415037
Q92604	5	6	4	LPGAT1	5	6	2.584963
Q92600	1	2	2	CNOT9	1.666667	2.666667	1.415037
Q92598	11	13	9	HSPH1	11	12	3.584963
Q92552	16	17	18	MRPS27	17	18	4.169925
Q92545	3	3	5	TMEM131	3.666667	4.666667	2.222392
Q92542	6	4	6	NCSTN	5.333333	6.333333	2.662965
Q92538	10	13	12	GBF1	11.66667	12.66667	3.662965
Q92522	13	12	11	H1-10	12	13	3.70044
Q92504	4	6	3	SLC39A7	4.333333	5.333333	2.415037
Q92499	29	24	31	DDX1	28	29	4.857981
Q8WYQ5	1	1	1	DGCR8	1	2	1
Q8WY22	5	6	4	BRI3BP	5	6	2.584963
Q8WXX5	10	11	12	DNAJC9	11	12	3.584963
Q8WXD5	4	3	1	GEMIN6	2.666667	3.666667	1.874469
Q8WWY3	2	4	3	PRPF31	3	4	2
Q8WWM7	2	4	2	ATXN2L	2.666667	3.666667	1.874469
Q8WWK9	10	12	10	CKAP2	10.66667	11.66667	3.544321
Q8WWC4	4	8	6	MAIP1	6	7	2.807355
Q8WWB7	2	1	1	GLMP	1.333333	2.333333	1.222392
Q8WW33	2	1	1	GTSF1	1.333333	2.333333	1.222392
Q8WVX9	9	8	8	FAR1	8.333333	9.333333	3.222392
Q8WVV9	10	11	12	HNRNPLL	11	12	3.584963
Q8WVM0	9	11	11	TFB1M	10.33333	11.33333	3.5025
Q8WVD3	2	2	1	RNF138	1.666667	2.666667	1.415037
Q8WUY1	3	2	3	THEM6	2.666667	3.666667	1.874469
Q8WUM4	4	3	6	PDCD6IP	4.333333	5.333333	2.415037
Q8WUM0	5	8	9	NUP133	7.333333	8.333333	3.058894
Q8WUA4	18	24	21	GTF3C2	21	22	4.459432
Q8WU90	9	10	10	ZC3H15	9.666667	10.66667	3.415037
Q8WTW3	2	5	5	COG1	4	5	2.321928
Q8TF76	2	1	2	HASPIN	1.666667	2.666667	1.415037
Q8TEY7	7	5	8	USP33	6.666667	7.666667	2.938599

Q8TEX9	35	38	40	IPO4	37.66667	38.66667	5.273018
Q8TER5	2	1	1	ARHGEF40	1.333333	2.333333	1.222392
Q8TEQ8	4	3	2	PIGO	3	4	2
Q8TEQ6	48	43	42	GEMIN5	44.33333	45.33333	5.5025
Q8TEM1	42	37	46	NUP210	41.66667	42.66667	5.415037
Q8TED1	3	5	5	GPX8	4.333333	5.333333	2.415037
Q8TDX7	3	4	3	NEK7	3.333333	4.333333	2.115477
Q8TDN6	20	21	20	BRIX1	20.33333	21.33333	4.415037
Q8TDD1	17	19	21	DDX54	19	20	4.321928
Q8TCY9	5	6	6	URGCP	5.666667	6.666667	2.736966
Q8TCJ2	10	9	12	STT3B	10.33333	11.33333	3.5025
Q8TCG1	25	22	21	CIP2A	22.66667	23.66667	4.564785
Q8TC07	2	6	4	TBC1D15	4	5	2.321928
Q8TAQ2	4	4	6	SMARCC2	4.666667	5.666667	2.5025
Q8TAG9	6	4	6	EXOC6	5.333333	6.333333	2.662965
Q8NI60	5	8	7	COQ8A	6.666667	7.666667	2.938599
Q8NI27	6	5	6	THOC2	5.666667	6.666667	2.736966
Q8NHQ9	19	15	19	DDX55	17.66667	18.66667	4.222392
Q8NG27	8	7	7	PJA1	7.333333	8.333333	3.058894
Q8NFW8	10	14	14	CMAS	12.66667	13.66667	3.77259
Q8NFF5	1	2	3	FLAD1	2	3	1.584963
Q8NF37	15	17	17	LPCAT1	16.33333	17.33333	4.115477
Q8NEZ5	2	1	1	FBXO22	1.333333	2.333333	1.222392
Q8NEW0	1	3	3	SLC30A7	2.333333	3.333333	1.736966
Q8NE71	16	21	16	ABCF1	17.66667	18.66667	4.222392
Q8NE01	12	15	15	CNNM3	14	15	3.906891
Q8NDZ4	9	9	9	DIPK2A	9	10	3.321928
Q8NCM8	1	1	2	DYNC2H1	1.333333	2.333333	1.222392
Q8NCA5	5	5	5	FAM98A	5	6	2.584963
Q8NC69	4	3	3	KCTD6	3.333333	4.333333	2.115477
Q8NC60	5	6	7	NOA1	6	7	2.807355
Q8NC51	34	36	34	SERBP1	34.66667	35.66667	5.156504
Q8NBU5	3	2	3	ATAD1	2.666667	3.666667	1.874469
Q8NBS9	6	5	7	TXNDC5	6	7	2.807355
Q8NBQ5	7	9	9	HSD17B11	8.333333	9.333333	3.222392
Q8NBN7	1	2	2	RDH13	1.666667	2.666667	1.415037
Q8NBM4	5	7	5	UBAC2	5.666667	6.666667	2.736966
Q8NB90	13	10	11	SPATA5	11.33333	12.33333	3.624491
Q8NB46	8	10	12	ANKRD52	10	11	3.459432
Q8NAT1	1	2	4	POMGNT2	2.333333	3.333333	1.736966
Q8N9T8	12	10	12	KRI1	11.33333	12.33333	3.624491
Q8N8A6	9	7	8	DDX51	8	9	3.169925
Q8N7H5	9	11	8	PAF1	9.333333	10.33333	3.369234
Q8N726	2	3	2	CDKN2A	2.333333	3.333333	1.736966
Q8N6R0	8	8	9	METTL13	8.333333	9.333333	3.222392
Q8N6M3	3	4	3	FITM2	3.333333	4.333333	2.115477
Q8N684	4	3	4	CPSF7	3.666667	4.666667	2.222392
Q8N5Y8	3	3	3	PARP16	3	4	2
Q8N5L8	3	4	3	RPP25L	3.333333	4.333333	2.115477
Q8N5K1	5	4	5	CISD2	4.666667	5.666667	2.5025
Q8N5F7	4	3	5	NKAP	4	5	2.321928
Q8N5C6	18	13	20	SRBD1	17	18	4.169925
Q8N5B7	4	4	4	CERS5	4	5	2.321928
Q8N3C0	22	20	20	ASCC3	20.66667	21.66667	4.437405
Q8N2K0	4	3	4	ABHD12	3.666667	4.666667	2.222392
Q8N2G8	1	2	3	GHDC	2	3	1.584963

Q8N201	5	2	7	INTS1	4.666667	5.666667	2.5025
Q8N1G2	2	1	2	CMTR1	1.666667	2.666667	1.415037
Q8N1F8	2	2	2	STK11IP	2	3	1.584963
Q8N1F7	10	11	10	NUP93	10.33333	11.33333	3.5025
Q8N122	1	3	3	RPTOR	2.333333	3.333333	1.736966
Q8IZP2	2	3	3	ST13P4	2.666667	3.666667	1.874469
Q8IZL8	4	4	5	PELP1	4.333333	5.333333	2.415037
Q8IZH2	20	21	22	XRN1	21	22	4.459432
Q8IZ69	11	6	9	TRMT2A	8.666667	9.666667	3.273018
Q8IY81	21	26	22	FTSJ3	23	24	4.584963
Q8IY37	14	13	18	DHX37	15	16	4
Q8IY17	15	11	10	PNPLA6	12	13	3.70044
Q8IXM3	2	3	2	MRPL41	2.333333	3.333333	1.736966
Q8IXI2	4	5	6	RHOT1	5	6	2.584963
Q8IXI1	4	4	7	RHOT2	5	6	2.584963
Q8IXB1	3	4	5	DNAJC10	4	5	2.321928
Q8IX12	1	1	1	CCAR1	1	2	1
Q8IWZ3	11	15	14	ANKHD1	13.33333	14.33333	3.841302
Q8IWX8	7	6	6	CHERP	6.333333	7.333333	2.874469
Q8IWS0	21	29	26	PHF6	25.33333	26.33333	4.718818
Q8IWA0	8	6	7	WDR75	7	8	3
Q8IVF7	5	6	6	FMNL3	5.666667	6.666667	2.736966
Q8IV08	10	10	10	PLD3	10	11	3.459432
Q8IUR0	2	2	1	TRAPPC5	1.666667	2.666667	1.415037
Q8IUF8	10	10	11	RIOX2	10.33333	11.33333	3.5025
Q86Y91	1	1	1	KIF18B	1	2	1
Q86Y56	5	11	5	DNAAF5	7	8	3
Q86Y39	1	2	1	NDUFA11	1.333333	2.333333	1.222392
Q86Y07	13	12	12	VRK2	12.33333	13.33333	3.736966
Q86XI2	19	19	17	NCAPG2	18.33333	19.33333	4.273018
Q86X55	1	1	1	CARM1	1	2	1
Q86VR2	7	9	13	RETREG3	9.666667	10.66667	3.415037
Q86VP6	11	11	14	CAND1	12	13	3.70044
Q86VM9	7	5	6	ZC3H18	6	7	2.807355
Q86VI3	3	4	6	IQGAP3	4.333333	5.333333	2.415037
Q86V81	11	14	14	ALYREF	13	14	3.807355
Q86UP2	65	60	67	KTN1	64	65	6.022368
Q86UL3	7	9	7	GPAT4	7.666667	8.666667	3.115477
Q86UK7	3	6	4	ZNF598	4.333333	5.333333	2.415037
Q86UE4	46	39	42	MTDH	42.33333	43.33333	5.437405
Q86U86	5	7	7	PBRM1	6.333333	7.333333	2.874469
Q86U38	19	17	19	NOP9	18.33333	19.33333	4.273018
Q86TB9	5	5	5	PATL1	5	6	2.584963
Q86T03	1	1	2	PIP4P1	1.333333	2.333333	1.222392
Q86SQ9	1	1	1	DHDDS	1	2	1
Q7Z7H8	5	5	4	MRPL10	4.666667	5.666667	2.5025
Q7Z7E8	3	3	4	UBE2Q1	3.333333	4.333333	2.115477
Q7Z6Z7	11	8	10	HUWE1	9.666667	10.66667	3.415037
Q7Z6E9	2	1	2	RBBP6	1.666667	2.666667	1.415037
Q7Z5H4	1	1	1	VN1R5	1	2	1
Q7Z4Q2	13	15	15	HEATR3	14.33333	15.33333	3.938599
Q7Z417	4	1	5	NUFIP2	3.333333	4.333333	2.115477
Q7Z3U7	19	25	30	MON2	24.66667	25.66667	4.681824
Q7Z2W9	5	3	5	MRPL21	4.333333	5.333333	2.415037
Q7Z2W4	53	57	63	ZC3HAV1	57.66667	58.66667	5.874469
Q7Z2T5	11	10	10	TRMT1L	10.33333	11.33333	3.5025

Q7RTV0	1	1	1	PHF5A	1	2	1
Q7LGA3	6	3	4	HS2ST1	4.333333	5.333333	2.415037
Q7L8L6	1	2	2	FASTKD5	1.666667	2.666667	1.415037
Q7L804	1	2	2	RAB11FIP2	1.666667	2.666667	1.415037
Q7L3T8	8	10	7	PARS2	8.333333	9.333333	3.222392
Q7L2J0	3	4	7	MEPCE	4.666667	5.666667	2.5025
Q7L2E3	108	113	107	DHX30	109.3333	110.3333	6.785725
Q7L0Y3	40	39	44	TRMT10C	41	42	5.392317
Q7KZN9	4	5	5	COX15	4.666667	5.666667	2.5025
Q7KZF4	24	22	27	SND1	24.33333	25.33333	4.662965
Q71U36	134	136	133	TUBA1A	134.3333	135.3333	7.080373
Q71RC2	19	16	18	LARP4	17.66667	18.66667	4.222392
Q71DI3	15	12	11	H3C	12.66667	13.66667	3.77259
Q70CQ2	93	94	104	USP34	97	98	6.61471
Q709F0	3	2	3	ACAD11	2.666667	3.666667	1.874469
Q6ZRQ5	3	4	3	MMS22L	3.333333	4.333333	2.115477
Q6ZRP7	3	5	3	QSOX2	3.666667	4.666667	2.222392
Q6ZNB6	11	11	15	NFXL1	12.33333	13.33333	3.736966
Q6YN16	3	4	1	HSDL2	2.666667	3.666667	1.874469
Q6YHU6	7	7	8	THADA	7.333333	8.333333	3.058894
Q6Y7W6	3	1	3	GIGYF2	2.333333	3.333333	1.736966
Q6UXN9	6	4	4	WDR82	4.666667	5.666667	2.5025
Q6UXM1	2	2	3	LRIG3	2.333333	3.333333	1.736966
Q6UX53	2	3	4	TMT1B	3	4	2
Q6UN15	6	5	3	FIP1L1	4.666667	5.666667	2.5025
Q6UB35	10	8	11	MTHFD1L	9.666667	10.66667	3.415037
Q6PKG0	41	41	38	LARP1	40	41	5.357552
Q6PJG6	13	11	14	BRAT1	12.66667	13.66667	3.77259
Q6PGP7	7	6	6	SKIC3	6.333333	7.333333	2.874469
Q6PCB5	4	2	5	RSBN1L	3.666667	4.666667	2.222392
Q6P996	4	6	7	PDXDC1	5.666667	6.666667	2.736966
Q6P6C2	4	4	3	ALKBH5	3.666667	4.666667	2.222392
Q6P5R6	11	12	10	RPL22L1	11	12	3.584963
Q6P4Q7	3	3	3	CNNM4	3	4	2
Q6P4A7	10	9	13	SFXN4	10.66667	11.66667	3.544321
Q6P3X3	3	4	5	TTC27	4	5	2.321928
Q6P2Q9	43	48	49	PRPF8	46.66667	47.66667	5.574909
Q6P1M0	12	10	11	SLC27A4	11	12	3.584963
Q6P1J9	8	4	5	CDC73	5.666667	6.666667	2.736966
Q6P161	1	3	5	MRPL54	3	4	2
Q6P158	38	40	36	DHX57	38	39	5.285402
Q6NXE6	3	4	1	ARMC6	2.666667	3.666667	1.874469
Q6NUQ1	2	3	5	RINT1	3.333333	4.333333	2.115477
Q6NTF9	3	1	3	RHBDD2	2.333333	3.333333	1.736966
Q6L8Q7	3	3	6	PDE12	4	5	2.321928
Q6IBW4	7	7	7	NCAPH2	7	8	3
Q6IAN0	11	13	11	DHRS7B	11.66667	12.66667	3.662965
Q6IAA8	2	3	2	LAMTOR1	2.333333	3.333333	1.736966
Q6DN90	2	2	1	IQSEC1	1.666667	2.666667	1.415037
Q6DKI1	8	9	9	RPL7L1	8.666667	9.666667	3.273018
Q6DD88	1	2	3	ATL3	2	3	1.584963
Q6AI08	1	2	2	HEATR6	1.666667	2.666667	1.415037
Q68E01	5	7	7	INTS3	6.333333	7.333333	2.874469
Q68CQ7	1	2	1	GLT8D1	1.333333	2.333333	1.222392
Q66K14	1	6	4	TBC1D9B	3.666667	4.666667	2.222392
Q659C4	8	10	10	LARP1B	9.333333	10.33333	3.369234

Q658Y4	7	7	8	FAM91A1	7.333333	8.333333	3.058894
Q5XKP0	9	9	10	MICOS13	9.333333	10.33333	3.369234
Q5VZL5	22	24	25	ZMYM4	23.66667	24.66667	4.624491
Q5VYK3	12	7	14	ECPAS	11	12	3.584963
Q5VWQ0	5	5	6	RSBN1	5.333333	6.333333	2.662965
Q5VV42	16	22	18	CDKAL1	18.66667	19.66667	4.297681
Q5VUA4	4	5	2	ZNF318	3.666667	4.666667	2.222392
Q5VTU8	3	2	4	ATP5F1EP2	3	4	2
Q5VTL8	5	3	4	PRPF38B	4	5	2.321928
Q5VST6	3	3	3	ABHD17B	3	4	2
Q5UIP0	13	12	14	RIF1	13	14	3.807355
Q5T9L3	4	3	3	WLS	3.333333	4.333333	2.115477
Q5T9A4	24	24	23	ATAD3B	23.66667	24.66667	4.624491
Q5T8P6	12	11	11	RBM26	11.33333	12.33333	3.624491
Q5T440	5	8	6	IBA57	6.333333	7.333333	2.874469
Q5T3I0	7	6	6	GPATCH4	6.333333	7.333333	2.874469
Q5T160	4	6	2	RARS2	4	5	2.321928
Q5SY16	7	4	3	NOL9	4.666667	5.666667	2.5025
Q5SWX8	2	2	1	ODR4	1.666667	2.666667	1.415037
Q5SSJ5	8	9	8	HP1BP3	8.333333	9.333333	3.222392
Q5SRE5	2	3	1	NUP188	2	3	1.584963
Q5SNT2	1	1	1	TMEM201	1	2	1
Q5QNW6	25	25	27	H2BC18	25.66667	26.66667	4.736966
Q5PRF9	8	8	5	SAMD4B	7	8	3
Q5MNZ6	3	2	5	WDR45B	3.333333	4.333333	2.115477
Q5JWR5	1	2	1	DOP1A	1.333333	2.333333	1.222392
Q5JWF2	4	3	4	GNAS	3.666667	4.666667	2.222392
Q5JTZ9	6	9	7	AARS2	7.333333	8.333333	3.058894
Q5JTH9	59	58	68	RRP12	61.66667	62.66667	5.969626
Q5JPH6	4	11	5	EARS2	6.666667	7.666667	2.938599
Q5JPE7	19	24	24	NOMO2	22.33333	23.33333	4.544321
Q5HYK3	5	5	6	COQ5	5.333333	6.333333	2.662965
Q5HYI8	2	2	5	RABL3	3	4	2
Q5H9R7	25	27	25	PPP6R3	25.66667	26.66667	4.736966
Q5DJT8	2	2	2	CT45A	2	3	1.584963
Q5C9Z4	26	27	28	NOM1	27	28	4.807355
Q5BKT4	1	1	1	ALG10	1	2	1
Q53S58	3	3	3	TMEM177	3	4	2
Q53H12	21	18	20	AGK	19.66667	20.66667	4.369234
Q53GQ0	12	14	17	HSD17B12	14.33333	15.33333	3.938599
Q53EP0	6	3	6	FNDC3B	5	6	2.584963
Q4VCS5	1	1	1	AMOT	1	2	1
Q49A26	11	13	8	GLYR1	10.66667	11.66667	3.544321
Q3ZCQ8	17	18	18	TIMM50	17.66667	18.66667	4.222392
Q3KQU3	7	8	7	MAP7D1	7.333333	8.333333	3.058894
Q2TAY7	10	5	5	SMU1	6.666667	7.666667	2.938599
Q2NL82	36	32	38	TSR1	35.33333	36.33333	5.183222
Q29RF7	4	9	8	PDS5A	7	8	3
Q1KMD3	11	8	9	HNRNPUL2	9.333333	10.33333	3.369234
Q16891	6	5	5	IMMT	5.333333	6.333333	2.662965
Q16795	1	3	5	NDUFA9	3	4	2
Q16778	23	23	26	H2BC21	24	25	4.643856
Q16777	24	22	21	H2AC20	22.33333	23.33333	4.544321
Q16718	5	4	5	NDUFA5	4.666667	5.666667	2.5025
Q16637	14	12	12	SMN	12.66667	13.66667	3.77259
Q16630	7	4	3	CPSF6	4.666667	5.666667	2.5025

Q16629	10	15	12	SRSF7	12.33333	13.33333	3.736966
Q16576	12	12	13	RBBP7	12.33333	13.33333	3.736966
Q16540	4	3	4	MRPL23	3.666667	4.666667	2.222392
Q16531	39	42	45	DDB1	42	43	5.426265
Q16527	14	13	14	CSRP2	13.66667	14.66667	3.874469
Q15904	6	7	7	ATP6AP1	6.666667	7.666667	2.938599
Q15773	2	1	1	MLF2	1.333333	2.333333	1.222392
Q15758	8	8	9	SLC1A5	8.333333	9.333333	3.222392
Q15717	5	2	6	ELAVL1	4.333333	5.333333	2.415037
Q15648	1	1	1	MED1	1	2	1
Q15646	4	2	4	OASL	3.333333	4.333333	2.115477
Q15645	6	9	8	TRIP13	7.666667	8.666667	3.115477
Q15637	3	3	4	SF1	3.333333	4.333333	2.115477
Q15633	3	2	3	TARBP2	2.666667	3.666667	1.874469
Q15629	5	5	4	TRAM1	4.666667	5.666667	2.5025
Q15572	2	1	2	TAF1C	1.666667	2.666667	1.415037
Q15477	3	4	5	SKIC2	4	5	2.321928
Q15437	8	9	10	SEC23B	9	10	3.321928
Q15436	14	10	16	SEC23A	13.33333	14.33333	3.841302
Q15427	2	2	3	SF3B4	2.333333	3.333333	1.736966
Q15393	23	24	24	SF3B3	23.66667	24.66667	4.624491
Q15392	21	21	21	DHCR24	21	22	4.459432
P61020	2	6	5	TOMM20	4.333333	5.333333	2.415037
Q15386	4	9	5	UBE3C	6	7	2.807355
Q15370	6	3	3	ELOB	4	5	2.321928
Q15369	5	4	6	ELOC	5	6	2.584963
Q15366	21	15	20	PCBP2	18.66667	19.66667	4.297681
Q15365	25	22	23	PCBP1	23.33333	24.33333	4.604862
Q15345	8	5	4	LRRC41	5.666667	6.666667	2.736966
Q15293	4	3	1	RCN1	2.666667	3.666667	1.874469
Q15269	4	5	7	PWP2	5.333333	6.333333	2.662965
Q15233	28	29	36	NONO	31	32	5
Q15185	1	2	3	PTGES3	2	3	1.584963
Q15120	4	5	7	PDK3	5.333333	6.333333	2.662965
Q15084	10	11	10	PDIA6	10.33333	11.33333	3.5025
Q15070	9	7	7	OXA1L	7.666667	8.666667	3.115477
Q15061	5	5	3	WDR43	4.333333	5.333333	2.415037
Q15058	4	2	4	KIF14	3.333333	4.333333	2.115477
Q15054	3	2	3	POLD3	2.666667	3.666667	1.874469
Q15050	18	16	13	RRS1	15.66667	16.66667	4.058894
Q15046	3	2	3	KARS1	2.666667	3.666667	1.874469
Q15043	3	3	2	SLC39A14	2.666667	3.666667	1.874469
Q15041	5	7	7	ARL6IP1	6.333333	7.333333	2.874469
Q15029	27	24	22	EFTUD2	24.33333	25.33333	4.662965
Q15024	7	8	5	EXOSC7	6.666667	7.666667	2.938599
Q15021	7	12	8	NCAPD2	9	10	3.321928
Q15020	8	13	13	SART3	11.33333	12.33333	3.624491
Q15014	3	3	4	MORF4L2	3.333333	4.333333	2.115477
Q15008	4	3	2	PSMD6	3	4	2
Q15003	1	1	1	NCAPH	1	2	1
Q14974	42	50	45	KPNB1	45.66667	46.66667	5.544321
Q14964	3	2	2	RAB39A	2.333333	3.333333	1.736966
Q14839	8	8	5	CHD4	7	8	3
Q14807	2	2	5	KIF22	3	4	2
Q14746	2	2	1	COG2	1.666667	2.666667	1.415037
Q14739	3	3	4	LBR	3.333333	4.333333	2.115477

Q14738	4	2	3	PPP2R5D	3	4	2
Q14697	5	7	6	GANAB	6	7	2.807355
Q14694	49	45	47	USP10	47	48	5.584963
Q14690	7	8	10	PDCD11	8.333333	9.333333	3.222392
Q14684	26	29	22	RRP1B	25.66667	26.66667	4.736966
Q14683	5	6	5	SMC1A	5.333333	6.333333	2.662965
Q14680	2	4	6	MELK	4	5	2.321928
Q14669	20	19	18	TRIP12	19	20	4.321928
Q14657	1	1	1	LAGE3	1	2	1
Q14574	3	4	1	DSC3	2.666667	3.666667	1.874469
Q14571	6	7	8	ITPR2	7	8	3
Q14534	3	3	2	SQLE	2.666667	3.666667	1.874469
Q14527	14	13	14	HLTF	13.66667	14.66667	3.874469
Q14498	37	36	30	RBM39	34.33333	35.33333	5.142958
Q14493	3	4	2	SLBP	3	4	2
Q14444	26	27	26	CAPRIN1	26.33333	27.33333	4.77259
Q14318	10	8	11	FKBP8	9.666667	10.66667	3.415037
Q14258	12	10	10	TRIM25	10.66667	11.66667	3.544321
Q14257	18	11	15	RCN2	14.66667	15.66667	3.969626
Q14232	9	5	9	EIF2B1	7.666667	8.666667	3.115477
Q14202	15	16	22	ZMYM3	17.66667	18.66667	4.222392
Q14165	6	6	7	MLEC	6.333333	7.333333	2.874469
Q14160	9	8	9	SCRIB	8.666667	9.666667	3.273018
Q14157	3	2	2	UBAP2L	2.333333	3.333333	1.736966
Q14137	10	11	10	BOP1	10.33333	11.33333	3.5025
Q14126	6	11	10	DSG2	9	10	3.321928
Q14103	15	11	14	HNRNPD	13.33333	14.33333	3.841302
Q14008	8	6	8	CKAP5	7.333333	8.333333	3.058894
Q13895	20	20	18	BYSL	19.33333	20.33333	4.345775
Q13868	7	9	7	EXOSC2	7.666667	8.666667	3.115477
Q13823	12	16	13	GNL2	13.66667	14.66667	3.874469
Q13765	1	1	1	NACA	1	2	1
Q13724	37	45	50	MOGS	44	45	5.491853
Q13642	1	2	1	FHL1	1.333333	2.333333	1.222392
Q13610	12	8	10	PWP1	10	11	3.459432
Q13596	1	1	1	SNX1	1	2	1
Q13585	401	422	392	GPR50	405	406	8.665336
Q13573	7	9	6	SNW1	7.333333	8.333333	3.058894
Q13547	11	10	11	HDAC1	10.66667	11.66667	3.544321
Q13523	11	9	11	PRPF4B	10.33333	11.33333	3.5025
Q13501	6	7	5	SQSTM1	6	7	2.807355
Q13435	26	27	24	SF3B2	25.66667	26.66667	4.736966
Q13428	6	4	7	TCOF1	5.666667	6.666667	2.736966
Q13418	3	3	2	ILK	2.666667	3.666667	1.874469
Q13405	1	3	1	MRPL49	1.666667	2.666667	1.415037
Q13367	1	1	1	AP3B2	1	2	1
Q13363	2	3	2	CTBP1	2.333333	3.333333	1.736966
Q13356	4	4	4	PPIL2	4	5	2.321928
Q13325	8	5	6	IFIT5	6.333333	7.333333	2.874469
Q13315	2	2	1	ATM	1.666667	2.666667	1.415037
Q13310	26	21	23	PABPC4	23.33333	24.33333	4.604862
Q13286	1	2	2	CLN3	1.666667	2.666667	1.415037
Q13283	20	18	16	G3BP1	18	19	4.247928
Q13263	16	20	15	TRIM28	17	18	4.169925
Q13247	17	21	18	SRSF6	18.66667	19.66667	4.297681
Q13243	13	10	12	SRSF5	11.66667	12.66667	3.662965

Q13242	26	25	23	SRSF9	24.66667	25.66667	4.681824
Q13206	9	8	10	DDX10	9	10	3.321928
Q13200	15	14	14	PSMD2	14.33333	15.33333	3.938599
Q13190	2	1	1	STX5	1.333333	2.333333	1.222392
Q13185	2	4	3	CBX3	3	4	2
Q13155	7	7	6	AIMP2	6.666667	7.666667	2.938599
Q13151	11	16	15	HNRNPA0	14	15	3.906891
Q13148	20	19	19	TARDBP	19.33333	20.33333	4.345775
Q13144	29	31	34	EIF2B5	31.33333	32.33333	5.01495
Q13123	2	3	1	IK	2	3	1.584963
Q13085	3	1	5	ACACA	3	4	2
Q13057	3	3	4	COASY	3.333333	4.333333	2.115477
Q13049	5	6	5	TRIM32	5.333333	6.333333	2.662965
Q13045	3	3	4	FLII	3.333333	4.333333	2.115477
Q13015	5	3	3	MLLT11	3.666667	4.666667	2.222392
Q12907	4	7	4	LMAN2	5	6	2.584963
Q12906	45	46	44	ILF3	45	46	5.523562
Q12905	39	33	37	ILF2	36.33333	37.33333	5.222392
Q12904	5	8	5	AIMP1	6	7	2.807355
Q12899	17	19	19	TRIM26	18.33333	19.33333	4.273018
Q12894	5	3	7	IFRD2	5	6	2.584963
Q12849	20	22	18	GRSF1	20	21	4.392317
Q12834	1	3	2	CDC20	2	3	1.584963
Q12824	1	2	1	SMARCB1	1.333333	2.333333	1.222392
Q12797	45	42	50	ASPH	45.66667	46.66667	5.544321
Q12789	30	19	21	GTF3C1	23.33333	24.33333	4.604862
Q12769	8	8	7	NUP160	7.666667	8.666667	3.115477
Q10570	6	5	3	CPSF1	4.666667	5.666667	2.5025
Q10471	1	1	1	GALNT2	1	2	1
Q09161	16	20	18	NCBP1	18	19	4.247928
Q09028	10	11	12	RBBP4	11	12	3.584963
Q08J23	30	26	27	NSUN2	27.66667	28.66667	4.841302
Q08AM6	4	3	3	VAC14	3.333333	4.333333	2.115477
Q08379	4	5	5	GOLGA2	4.666667	5.666667	2.5025
Q08211	98	93	101	DHX9	97.33333	98.33333	6.619609
Q08209	5	7	11	PPP3CA	7.666667	8.666667	3.115477
Q07955	18	19	20	SRSF1	19	20	4.321928
Q07864	13	14	15	POLE	14	15	3.906891
Q07666	13	10	11	KHDRBS1	11.33333	12.33333	3.624491
Q07065	48	49	47	CKAP4	48	49	5.61471
Q07021	10	9	10	C1QBP	9.666667	10.66667	3.415037
Q07020	35	32	31	RPL18	32.66667	33.66667	5.073249
Q06830	53	45	47	PRDX1	48.33333	49.33333	5.624491
Q06787	9	13	9	FMR1	10.33333	11.33333	3.5025
Q06330	3	5	4	RBPJ	4	5	2.321928
Q06265	3	2	2	EXOSC9	2.333333	3.333333	1.736966
Q06136	1	2	2	KDSR	1.666667	2.666667	1.415037
Q05932	5	2	5	FPGS	4	5	2.321928
Q05519	9	7	8	SRSF11	8	9	3.169925
Q04721	3	2	2	NOTCH2	2.333333	3.333333	1.736966
Q02978	20	26	23	SLC25A11	23	24	4.584963
Q02880	15	18	14	TOP2B	15.66667	16.66667	4.058894
Q02878	62	61	58	RPL6	60.33333	61.33333	5.938599
Q02543	63	48	53	RPL18A	54.66667	55.66667	5.798742
Q02241	5	5	5	KIF23	5	6	2.584963
Q01844	1	2	2	EWSR1	1.666667	2.666667	1.415037

Q01813	4	4	4	PFKP	4	5	2.321928
Q01780	20	18	17	EXOSC10	18.33333	19.33333	4.273018
Q01650	2	2	4	SLC7A5	2.666667	3.666667	1.874469
Q01130	10	8	10	SRSF2	9.333333	10.33333	3.369234
Q01105	2	2	2	SET	2	3	1.584963
Q01081	16	17	15	U2AF1	16	17	4.087463
Q00839	88	79	85	HNRNPU	84	85	6.409391
Q00610	3	3	2	CLTC	2.666667	3.666667	1.874469
Q00577	15	17	21	PURA	17.66667	18.66667	4.222392
Q00535	8	11	8	CDK5	9	10	3.321928
Q00403	1	2	1	GTF2B	1.333333	2.333333	1.222392
Q00341	16	21	13	HDLBP	16.66667	17.66667	4.142958
Q00325	36	39	41	SLC25A3	38.66667	39.66667	5.309855
Q00059	1	1	2	TFAM	1.333333	2.333333	1.222392
P99999	2	2	2	CYCS	2	3	1.584963
P84243	13	10	10	H3-3A/H3-	11	12	3.584963
P84103	21	20	15	SRSF3	18.66667	19.66667	4.297681
P84098	32	26	25	RPL19	27.66667	28.66667	4.841302
P84090	5	5	6	ERH	5.333333	6.333333	2.662965
P83881	16	13	14	RPL36A	14.33333	15.33333	3.938599
P83731	28	26	33	RPL24	29	30	4.906891
P82933	15	13	18	MRPS9	15.33333	16.33333	4.029747
P82932	4	4	5	MRPS6	4.333333	5.333333	2.415037
P82930	13	14	15	MRPS34	14	15	3.906891
P82921	3	4	4	MRPS21	3.666667	4.666667	2.222392
P82914	9	8	10	MRPS15	9	10	3.321928
P82675	10	9	9	MRPS5	9.333333	10.33333	3.369234
P82673	10	11	10	MRPS35	10.33333	11.33333	3.5025
P82663	13	14	10	MRPS25	12.33333	13.33333	3.736966
P82650	24	23	31	MRPS22	26	27	4.754888
P79522	3	3	3	PRR3	3	4	2
P78527	190	187	199	PRKDC	192	193	7.592457
P78406	5	2	7	RAE1	4.666667	5.666667	2.5025
P78395	2	3	3	PRAME	2.666667	3.666667	1.874469
P78371	3	2	3	CCT2	2.666667	3.666667	1.874469
P78362	16	14	13	SRPK2	14.33333	15.33333	3.938599
P78347	8	8	7	GTF2I	7.666667	8.666667	3.115477
P78346	8	11	9	RPP30	9.333333	10.33333	3.369234
P78316	6	11	11	NOP14	9.333333	10.33333	3.369234
P69905	1	1	2	HBA	1.333333	2.333333	1.222392
P68431	15	10	11	H3C	12	13	3.70044
P68400	21	21	26	CSNK2A1	22.66667	23.66667	4.564785
P68371	166	157	147	TUBB4B	156.6667	157.6667	7.300734
P68363	134	139	134	TUBA1B	135.6667	136.6667	7.094518
P68133	39	36	31	ACTA1	35.33333	36.33333	5.183222
P68104	48	44	39	EEF1A1	43.66667	44.66667	5.481127
P68036	3	4	3	UBE2L3	3.333333	4.333333	2.115477
P67870	12	13	14	CSNK2B	13	14	3.807355
P67809	14	14	16	YBX1	14.66667	15.66667	3.969626
P67775	6	8	8	PPP2CA	7.333333	8.333333	3.058894
P63244	34	30	31	RACK1	31.66667	32.66667	5.029747
P63241	11	12	14	EIF5A	12.33333	13.33333	3.736966
P63208	4	6	4	SKP1	4.666667	5.666667	2.5025
P63173	29	32	25	RPL38	28.66667	29.66667	4.890771
P63167	8	9	11	DYNLL1	9.333333	10.33333	3.369234
P63151	4	6	6	PPP2R2A	5.333333	6.333333	2.662965

P63010	10	10	11	AP2B1	10.33333	11.33333	3.5025
P62995	6	7	5	TRA2B	6	7	2.807355
P62993	3	3	4	GRB2	3.333333	4.333333	2.115477
P62987	25	26	22	UBA52	24.33333	25.33333	4.662965
P62979	40	38	34	RPS27A	37.33333	38.33333	5.260528
P62937	20	25	22	PPIA	22.33333	23.33333	4.544321
P62917	57	55	51	RPL8	54.33333	55.33333	5.790077
P62913	22	21	16	RPL11	19.66667	20.66667	4.369234
P62910	47	39	38	RPL32	41.33333	42.33333	5.403722
P62906	78	78	73	RPL10A	76.33333	77.33333	6.273018
P62899	25	25	23	RPL31	24.33333	25.33333	4.662965
P62891	9	8	7	RPL39	8	9	3.169925
P62888	35	28	31	RPL30	31.33333	32.33333	5.01495
P62879	7	10	9	GNB2	8.666667	9.666667	3.273018
P62875	1	1	2	POLR2L	1.333333	2.333333	1.222392
P62873	5	8	5	GNB1	6	7	2.807355
P62861	10	11	12	FAU	11	12	3.584963
P62857	13	9	11	RPS28	11	12	3.584963
P62854	23	24	19	RPS26	22	23	4.523562
P62851	31	27	21	RPS25	26.33333	27.33333	4.77259
P62847	38	38	31	RPS24	35.66667	36.66667	5.196397
P62841	21	20	22	RPS15	21	22	4.459432
P62829	44	31	35	RPL23	36.66667	37.66667	5.235216
P62826	5	2	6	RAN	4.333333	5.333333	2.415037
P62805	29	24	25	H4C	26	27	4.754888
P62753	62	52	61	RPS6	58.33333	59.33333	5.890771
P62750	34	37	37	RPL23A	36	37	5.209453
P62701	129	123	102	RPS4X	118	119	6.894818
P62633	4	1	5	CNBP	3.333333	4.333333	2.115477
P62424	105	106	102	RPL7A	104.3333	105.3333	6.718818
P62333	7	7	11	PSMC6	8.333333	9.333333	3.222392
P62318	9	9	12	SNRPD3	10	11	3.459432
P62316	6	5	5	SNRPD2	5.333333	6.333333	2.662965
P62314	4	3	3	SNRPD1	3.333333	4.333333	2.115477
P62308	4	3	3	SNRPG	3.333333	4.333333	2.115477
P62304	5	4	5	SNRPE	4.666667	5.666667	2.5025
P62280	47	39	40	RPS11	42	43	5.426265
P62277	61	66	69	RPS13	65.33333	66.33333	6.051662
P62273	14	16	13	RPS29	14.33333	15.33333	3.938599
P62269	49	44	45	RPS18	46	47	5.554589
P62266	28	24	27	RPS23	26.33333	27.33333	4.77259
P62263	42	40	39	RPS14	40.33333	41.33333	5.369234
P62258	11	12	8	YWHAE	10.33333	11.33333	3.5025
P62249	53	53	62	RPS16	56	57	5.83289
P62244	53	43	43	RPS15A	46.33333	47.33333	5.564785
P62241	55	52	52	RPS8	53	54	5.754888
P62195	15	7	10	PSMC5	10.66667	11.66667	3.544321
P62191	3	4	4	PSMC1	3.666667	4.666667	2.222392
P62140	17	22	23	PPP1CB	20.66667	21.66667	4.437405
P62081	48	41	43	RPS7	44	45	5.491853
P61978	54	54	53	HNRNPK	53.66667	54.66667	5.77259
P61964	10	8	12	WDR5	10	11	3.459432
P61962	6	5	7	DCAF7	6	7	2.807355
P61927	7	6	6	RPL37	6.333333	7.333333	2.874469
P61769	1	1	1	B2M	1	2	1
P61758	2	1	2	VBP1	1.666667	2.666667	1.415037

P61626	1	1	1	LYZ	1	2	1
P61619	20	25	18	SEC61A1	21	22	4.459432
P61513	16	15	15	RPL37A	15.33333	16.33333	4.029747
P61421	3	3	3	ATP6V0D1	3	4	2
P61353	62	64	61	RPL27	62.33333	63.33333	5.984893
P61313	39	36	40	RPL15	38.33333	39.33333	5.297681
P61289	4	5	5	PSME3	4.666667	5.666667	2.5025
P61254	38	36	38	RPL26	37.33333	38.33333	5.260528
P61247	100	92	97	RPS3A	96.33333	97.33333	6.604862
P61204	6	6	8	ARF3	6.666667	7.666667	2.938599
P61201	1	1	1	COPS2	1	2	1
P61165	2	1	2	TMEM258	1.666667	2.666667	1.415037
P61160	4	6	5	ACTR2	5	6	2.584963
P61106	8	6	3	RAB14	5.666667	6.666667	2.736966
P61081	4	3	2	UBE2M	3	4	2
P61077	4	4	5	UBE2D3	4.333333	5.333333	2.415037
P61026	2	3	4	RAB10	3	4	2
P61020	5	5	3	RAB5B	4.333333	5.333333	2.415037
P60981	5	2	3	DSTN	3.333333	4.333333	2.115477
P60953	3	4	2	CDC42	3	4	2
P60903	6	4	6	S100A10	5.333333	6.333333	2.662965
P60891	19	21	19	PRPS1	19.66667	20.66667	4.369234
P60866	25	20	22	RPS20	22.33333	23.33333	4.544321
P60842	24	17	28	EIF4A1	23	24	4.584963
P60709	74	76	70	ACTB	73.33333	74.33333	6.215937
P60660	2	3	2	MYL6	2.333333	3.333333	1.736966
P60604	9	8	9	UBE2G2	8.666667	9.666667	3.273018
P60468	2	2	2	SEC61B	2	3	1.584963
P60059	1	2	1	SEC61G	1.333333	2.333333	1.222392
P58546	1	2	1	MTPN	1.333333	2.333333	1.222392
P57740	1	1	2	NUP107	1.333333	2.333333	1.222392
P57678	46	52	48	GEMIN4	48.66667	49.66667	5.634206
P57105	3	3	3	SYNJ2BP	3	4	2
P57088	27	27	24	TMEM33	26	27	4.754888
P56589	4	3	4	PEX3	3.666667	4.666667	2.222392
P56537	6	7	8	EIF6	7	8	3
P56385	8	5	7	ATP5ME	6.666667	7.666667	2.938599
P56270	9	8	9	MAZ	8.666667	9.666667	3.273018
P56192	40	35	35	MARS1	36.66667	37.66667	5.235216
P56182	12	13	13	RRP1	12.66667	13.66667	3.77259
P56134	8	8	6	ATP5MF	7.333333	8.333333	3.058894
P55795	29	29	29	HNRNPH2	29	30	4.906891
P55786	4	2	3	NPEPPS	3	4	2
P55769	4	4	3	SNU13	3.666667	4.666667	2.222392
P55735	2	2	2	SEC13	2	3	1.584963
P55265	26	31	28	ADAR	28.33333	29.33333	4.874469
P55209	38	31	35	NAP1L1	34.66667	35.66667	5.156504
P55196	1	1	3	AFDN	1.666667	2.666667	1.415037
P55084	18	18	23	HADHB	19.66667	20.66667	4.369234
P55072	4	9	7	VCP	6.666667	7.666667	2.938599
P55060	34	39	36	CSE1L	36.33333	37.33333	5.222392
P55036	4	2	6	PSMD4	4	5	2.321928
P54886	8	6	8	ALDH18A1	7.333333	8.333333	3.058894
P54709	8	5	7	ATP1B3	6.666667	7.666667	2.938599
P54619	4	5	3	PRKAG1	4	5	2.321928
P54136	20	21	21	RARS1	20.66667	21.66667	4.437405

P53992	22	22	22	SEC24C	22	23	4.523562
P53985	7	7	8	SLC16A1	7.333333	8.333333	3.058894
P53794	6	4	5	SLC5A3	5	6	2.584963
P53680	1	1	1	AP2S1	1	2	1
P53621	64	50	52	COPA	55.33333	56.33333	5.815917
P53618	9	11	11	COPB1	10.33333	11.33333	3.5025
P53597	1	1	1	SUCLG1	1	2	1
P53396	12	9	8	ACLY	9.666667	10.66667	3.415037
P53350	16	12	10	PLK1	12.66667	13.66667	3.77259
P53007	12	7	10	SLC25A1	9.666667	10.66667	3.415037
P52907	6	4	3	CAPZA1	4.333333	5.333333	2.415037
P52815	4	4	3	MRPL12	3.666667	4.666667	2.222392
P52799	2	2	2	EFNB2	2	3	1.584963
P52789	10	8	8	HK2	8.666667	9.666667	3.273018
P52758	4	3	4	RIDA	3.666667	4.666667	2.222392
P52701	17	23	21	MSH6	20.33333	21.33333	4.415037
P52597	44	40	41	HNRNPF	41.66667	42.66667	5.415037
P52434	2	1	2	POLR2H	1.666667	2.666667	1.415037
P52429	7	7	5	DGKE	6.333333	7.333333	2.874469
P52294	12	11	11	KPNA1	11.33333	12.33333	3.624491
P52292	32	29	28	KPNA2	29.66667	30.66667	4.938599
P52272	92	87	91	HNRNPM	90	91	6.507795
P51991	27	24	28	HNRNPA3	26.33333	27.33333	4.77259
P51665	3	3	2	PSMD7	2.666667	3.666667	1.874469
P51648	7	8	8	ALDH3A2	7.666667	8.666667	3.115477
P51617	6	6	6	IRAK1	6	7	2.807355
P51610	3	2	5	HCFC1	3.333333	4.333333	2.115477
P51571	14	14	12	SSR4	13.33333	14.33333	3.841302
P51570	5	4	5	GALK1	4.666667	5.666667	2.5025
P51553	1	1	1	IDH3G	1	2	1
P51532	4	4	7	SMARCA4	5	6	2.584963
P51530	2	1	4	DNA2	2.333333	3.333333	1.736966
P51398	23	19	18	DAP3	20	21	4.392317
P51148	5	6	7	RAB5C	6	7	2.807355
P51116	15	13	14	FXR2	14	15	3.906891
P51114	16	20	17	FXR1	17.66667	18.66667	4.222392
P50991	24	24	22	CCT4	23.33333	24.33333	4.604862
P50990	4	1	3	CCT8	2.666667	3.666667	1.874469
P50914	41	38	39	RPL14	39.33333	40.33333	5.333901
P50454	2	2	2	SERPINH1	2	3	1.584963
P50416	4	4	3	CPT1A	3.666667	4.666667	2.222392
P50402	15	12	17	EMD	14.66667	15.66667	3.969626
P50213	2	2	4	IDH3A	2.666667	3.666667	1.874469
P49916	1	2	1	LIG3	1.333333	2.333333	1.222392
P49915	10	9	11	GMPS	10	11	3.459432
P49810	2	2	2	PSEN2	2	3	1.584963
P49790	2	3	4	NUP153	3	4	2
P49770	7	7	12	EIF2B2	8.666667	9.666667	3.273018
P49756	5	3	3	RBM25	3.666667	4.666667	2.222392
P49721	3	3	2	PSMB2	2.666667	3.666667	1.874469
P49720	2	2	2	PSMB3	2	3	1.584963
P49711	8	8	8	CTCF	8	9	3.169925
P49589	1	1	2	CARS1	1.333333	2.333333	1.222392
P49588	1	1	2	AARS1	1.333333	2.333333	1.222392
P49585	1	3	2	PCYT1A	2	3	1.584963
P49458	7	7	8	SRP9	7.333333	8.333333	3.058894

P49411	30	33	29	TUFM	30.66667	31.66667	4.984893
P49368	20	20	21	CCT3	20.33333	21.33333	4.415037
P49327	41	48	42	FASN	43.66667	44.66667	5.481127
P49207	24	22	24	RPL34	23.33333	24.33333	4.604862
P48651	7	8	9	PTDSS1	8	9	3.169925
P48643	13	12	13	CCT5	12.66667	13.66667	3.77259
P48634	8	6	8	PRRC2A	7.333333	8.333333	3.058894
P48556	3	3	3	PSMD8	3	4	2
P48507	2	2	2	GCLM	2	3	1.584963
P48047	31	29	27	ATP5PO	29	30	4.906891
P47914	10	10	9	RPL29	9.666667	10.66667	3.415037
P47897	13	16	15	QARS1	14.66667	15.66667	3.969626
P47756	3	4	3	CAPZB	3.333333	4.333333	2.115477
P47755	2	3	2	CAPZA2	2.333333	3.333333	1.736966
P46977	15	17	16	STT3A	16	17	4.087463
P46940	22	26	29	IQGAP1	25.66667	26.66667	4.736966
P46783	65	63	58	RPS10	62	63	5.97728
P46782	57	53	54	RPS5	54.66667	55.66667	5.798742
P46781	89	81	82	RPS9	84	85	6.409391
P46779	36	30	31	RPL28	32.33333	33.33333	5.058894
P46778	35	30	38	RPL21	34.33333	35.33333	5.142958
P46777	39	34	33	RPL5	35.33333	36.33333	5.183222
P46776	29	30	29	RPL27A	29.33333	30.33333	4.922832
P46734	3	3	3	MAP2K3	3	4	2
P46379	9	10	9	BAG6	9.333333	10.33333	3.369234
P46109	13	15	15	CRKL	14.33333	15.33333	3.938599
P46087	23	24	23	NOP2	23.33333	24.33333	4.604862
P46060	2	2	3	RANGAP1	2.333333	3.333333	1.736966
P45974	6	5	10	USP5	7	8	3
P45954	2	3	2	ACADSB	2.333333	3.333333	1.736966
P45880	10	12	7	VDAC2	9.666667	10.66667	3.415037
P43686	13	12	12	PSMC4	12.33333	13.33333	3.736966
P43490	12	10	12	NAMPT	11.33333	12.33333	3.624491
P43355	3	3	3	MAGEA1	3	4	2
P43307	8	9	8	SSR1	8.333333	9.333333	3.222392
P43304	16	19	18	GPD2	17.66667	18.66667	4.222392
P43246	14	18	16	MSH2	16	17	4.087463
P43243	21	25	27	MATR3	24.33333	25.33333	4.662965
P43003	1	1	1	SLC1A3	1	2	1
P42766	26	24	26	RPL35	25.33333	26.33333	4.718818
P42704	25	35	31	LRPPRC	30.33333	31.33333	4.969626
P42695	20	22	22	NCAPD3	21.33333	22.33333	4.481127
P42677	15	15	14	RPS27	14.66667	15.66667	3.969626
P42356	9	6	7	PI4KA	7.333333	8.333333	3.058894
P42345	6	9	9	MTOR	8	9	3.169925
P42285	15	15	16	MTREX	15.33333	16.33333	4.029747
P42167	29	31	29	TMPO	29.66667	30.66667	4.938599
P41743	7	5	7	PRKCI	6.333333	7.333333	2.874469
P41252	25	30	26	IARS1	27	28	4.807355
P41250	4	8	10	GARS1	7.333333	8.333333	3.058894
P41240	6	5	5	CSK	5.333333	6.333333	2.662965
P41091	16	13	12	EIF2S3	13.66667	14.66667	3.874469
P40939	36	37	39	HADHA	37.33333	38.33333	5.260528
P40938	16	18	16	RFC3	16.66667	17.66667	4.142958
P40937	11	13	15	RFC5	13	14	3.807355
P40763	1	1	2	STAT3	1.333333	2.333333	1.222392

P40616	9	13	11	ARL1	11	12	3.584963
P40429	76	65	64	RPL13A	68.33333	69.33333	6.115477
P40227	8	7	8	CCT6A	7.666667	8.666667	3.115477
P39748	2	2	1	FEN1	1.666667	2.666667	1.415037
P39656	32	32	30	DDOST	31.33333	32.33333	5.01495
P39023	107	98	93	RPL3	99.33333	100.3333	6.648657
P39019	51	48	45	RPS19	48	49	5.61471
P38935	4	6	4	IGHMBP2	4.666667	5.666667	2.5025
P38919	8	7	8	EIF4A3	7.666667	8.666667	3.115477
P38646	21	26	23	HSPA9	23.33333	24.33333	4.604862
P38606	9	9	8	ATP6V1A	8.666667	9.666667	3.273018
P38435	1	2	1	GGCX	1.333333	2.333333	1.222392
P38159	6	8	8	RBMX	7.333333	8.333333	3.058894
P37802	4	4	7	TAGLN2	5	6	2.584963
P37268	6	7	6	FDFT1	6.333333	7.333333	2.874469
P37108	7	5	8	SRP14	6.666667	7.666667	2.938599
P36873	20	24	25	PPP1CC	23	24	4.584963
P36578	161	147	158	RPL4	155.3333	156.3333	7.288482
P36551	1	2	2	CPOX	1.666667	2.666667	1.415037
P36542	19	16	17	ATP5F1C	17.33333	18.33333	4.196397
P35998	13	9	11	PSMC2	11	12	3.584963
P35908	18	10	27	KRT2	18.33333	19.33333	4.273018
P35659	7	6	8	DEK	7	8	3
P35637	6	5	5	FUS	5.333333	6.333333	2.662965
P35613	5	4	3	BSG	4	5	2.321928
P35606	7	9	5	COPB2	7	8	3
P35527	14	14	20	KRT9	16	17	4.087463
P35268	33	31	30	RPL22	31.33333	32.33333	5.01495
P35251	18	19	16	RFC1	17.66667	18.66667	4.222392
P35250	14	14	15	RFC2	14.33333	15.33333	3.938599
P35249	16	14	16	RFC4	15.33333	16.33333	4.029747
P35237	1	4	2	SERPINB6	2.333333	3.333333	1.736966
P35232	5	6	5	PHB1	5.333333	6.333333	2.662965
P35222	3	4	4	CTNNB1	3.666667	4.666667	2.222392
P35080	5	7	7	PFN2	6.333333	7.333333	2.874469
P33993	28	30	26	MCM7	28	29	4.857981
P33992	47	41	42	MCM5	43.33333	44.33333	5.47032
P33991	2	3	4	MCM4	3	4	2
P33981	1	6	4	TTK	3.666667	4.666667	2.222392
P33947	2	2	2	KDELR2	2	3	1.584963
P32969	43	40	37	RPL9	40	41	5.357552
P32119	21	22	20	PRDX2	21	22	4.459432
P31943	44	40	47	HNRNPH1	43.66667	44.66667	5.481127
P31689	41	44	43	DNAJA1	42.66667	43.66667	5.448461
P31350	2	2	2	RRM2	2	3	1.584963
P31153	4	3	5	MAT2A	4	5	2.321928
P31040	2	2	2	SDHA	2	3	1.584963
P30876	8	7	8	POLR2B	7.666667	8.666667	3.115477
P30837	8	6	7	ALDH1B1	7	8	3
P30825	4	6	3	SLC7A1	4.333333	5.333333	2.415037
P30740	3	2	2	SERPINB1	2.333333	3.333333	1.736966
P30519	2	2	3	HMOX2	2.333333	3.333333	1.736966
P30414	9	11	9	NKTR	9.666667	10.66667	3.415037
P30260	4	4	3	CDC27	3.666667	4.666667	2.222392
P30154	10	11	9	PPP2R1B	10	11	3.459432
P30153	20	20	24	PPP2R1A	21.33333	22.33333	4.481127

P30101	1	1	1	PDIA3	1	2	1
P30050	39	37	34	RPL12	36.66667	37.66667	5.235216
P30049	2	2	2	ATP5F1D	2	3	1.584963
P30048	6	4	4	PRDX3	4.666667	5.666667	2.5025
P29401	5	3	3	TKT	3.666667	4.666667	2.222392
P28482	3	5	6	MAPK1	4.666667	5.666667	2.5025
P28340	8	8	8	POLD1	8	9	3.169925
P28288	5	5	5	ABCD3	5	6	2.584963
P28066	3	2	2	PSMA5	2.333333	3.333333	1.736966
P27824	17	20	17	CANX	18	19	4.247928
P27816	8	12	11	MAP4	10.33333	11.33333	3.5025
P27708	26	23	21	CAD	23.33333	24.33333	4.604862
P27635	62	59	64	RPL10	61.66667	62.66667	5.969626
P27544	3	2	3	CERS1	2.666667	3.666667	1.874469
P27348	14	11	8	YWHAQ	11	12	3.584963
P26641	20	20	20	EEF1G	20	21	4.392317
P26599	44	43	45	PTBP1	44	45	5.491853
P26373	71	61	59	RPL13	63.66667	64.66667	6.01495
P26368	48	46	46	U2AF2	46.66667	47.66667	5.574909
P26196	9	9	14	DDX6	10.66667	11.66667	3.544321
P25789	4	5	5	PSMA4	4.666667	5.666667	2.5025
P25705	89	82	75	ATP5F1A	82	83	6.375039
P25685	2	3	3	DNAJB1	2.666667	3.666667	1.874469
P25490	2	3	1	YY1	2	3	1.584963
P25398	39	35	31	RPS12	35	36	5.169925
P25205	55	57	57	MCM3	56.33333	57.33333	5.841302
P24928	1	3	1	POLR2A	1.666667	2.666667	1.415037
P24752	4	1	3	ACAT1	2.666667	3.666667	1.874469
P24666	7	9	10	ACP1	8.666667	9.666667	3.273018
P24539	13	15	16	ATP5PB	14.66667	15.66667	3.969626
P23921	3	2	2	RRM1	2.333333	3.333333	1.736966
P23528	12	12	9	CFL1	11	12	3.584963
P23396	122	114	109	RPS3	115	116	6.857981
P23284	5	7	6	PPIB	6	7	2.807355
P23246	28	29	27	SFPQ	28	29	4.857981
P22830	3	5	4	FECH	4	5	2.321928
P22695	6	5	7	UQCRC2	6	7	2.807355
P22626	17	17	19	HNRNPA2E	17.66667	18.66667	4.222392
P22392	16	16	15	NME2	15.66667	16.66667	4.058894
P22314	6	7	9	UBA1	7.333333	8.333333	3.058894
P22234	1	3	6	PAICS	3.333333	4.333333	2.115477
P22102	19	19	18	GART	18.66667	19.66667	4.297681
P22087	13	16	14	FBL	14.33333	15.33333	3.938599
P22061	14	10	9	PCMT1	11	12	3.584963
P21964	6	9	7	COMT	7.333333	8.333333	3.058894
P21912	2	1	2	SDHB	1.666667	2.666667	1.415037
P21796	5	3	3	VDAC1	3.666667	4.666667	2.222392
P21359	2	6	4	NF1	4	5	2.321928
P21333	3	3	5	FLNA	3.666667	4.666667	2.222392
P21266	1	1	2	GSTM3	1.333333	2.333333	1.222392
P21127	14	13	18	CDK11B	15	16	4
P20700	3	3	4	LMNB1	3.333333	4.333333	2.115477
P20674	4	4	4	COX5A	4	5	2.321928
P20618	1	1	3	PSMB1	1.666667	2.666667	1.415037
P19784	13	16	13	CSNK2A2	14	15	3.906891
P19623	1	2	1	SRM	1.333333	2.333333	1.222392

P19525	14	15	13	EIF2AK2	14	15	3.906891
P19387	1	1	2	POLR2C	1.333333	2.333333	1.222392
P19338	36	27	26	NCL	29.66667	30.66667	4.938599
P18859	7	7	8	ATP5PF	7.333333	8.333333	3.058894
P18621	63	50	61	RPL17	58	59	5.882643
P18583	6	10	6	SON	7.333333	8.333333	3.058894
P18124	87	78	76	RPL7	80.33333	81.33333	6.345775
P18085	10	14	12	ARF4	12	13	3.70044
P18077	36	31	32	RPL35A	33	34	5.087463
P18074	1	1	1	ERCC2	1	2	1
P18031	16	20	18	PTPN1	18	19	4.247928
P17987	27	27	25	TCP1	26.33333	27.33333	4.77259
P17980	14	13	17	PSMC3	14.66667	15.66667	3.969626
P17858	5	6	7	PFKL	6	7	2.807355
P17844	64	74	68	DDX5	68.66667	69.66667	6.122397
P17812	34	33	33	CTPS1	33.33333	34.33333	5.101538
P17480	2	3	3	UBTF	2.666667	3.666667	1.874469
P17302	3	3	2	GJA1	2.666667	3.666667	1.874469
P17152	2	2	2	TMEM11	2	3	1.584963
P16615	50	53	48	ATP2A2	50.33333	51.33333	5.681824
P16403	40	40	37	H1-2	39	40	5.321928
P16298	3	4	6	PPP3CB	4.333333	5.333333	2.415037
P15924	24	22	22	DSP	22.66667	23.66667	4.564785
P15880	68	61	58	RPS2	62.33333	63.33333	5.984893
P15531	14	13	10	NME1	12.33333	13.33333	3.736966
P14923	3	2	4	JUP	3	4	2
P14868	21	22	19	DARS1	20.66667	21.66667	4.437405
P14866	16	14	14	HNRNPL	14.66667	15.66667	3.969626
P14678	7	8	6	SNRPB	7	8	3
P14635	3	2	4	CCNB1	3	4	2
P14618	4	5	3	PKM	4	5	2.321928
P14174	36	36	34	MIF	35.33333	36.33333	5.183222
P13995	1	1	2	MTHFD2	1.333333	2.333333	1.222392
P13807	7	5	7	GYS1	6.333333	7.333333	2.874469
P13804	2	1	3	ETFA	2	3	1.584963
P13797	25	28	30	PLS3	27.66667	28.66667	4.841302
P13645	34	23	39	KRT10	32	33	5.044394
P13639	50	48	51	EEF2	49.66667	50.66667	5.662965
P13489	4	1	3	RNH1	2.666667	3.666667	1.874469
P13010	53	46	52	XRCC5	50.33333	51.33333	5.681824
P12956	50	39	41	XRCC6	43.33333	44.33333	5.47032
P12268	3	4	4	IMPDH2	3.666667	4.666667	2.222392
P12236	62	56	56	SLC25A6	58	59	5.882643
P12235	56	50	52	SLC25A4	52.66667	53.66667	5.745954
P12004	19	20	16	PCNA	18.33333	19.33333	4.273018
P11940	53	43	44	PABPC1	46.66667	47.66667	5.574909
P11717	5	9	9	IGF2R	7.666667	8.666667	3.115477
P11586	12	11	10	MTHFD1	11	12	3.584963
P11441	1	2	2	UBL4A	1.666667	2.666667	1.415037
P11388	19	22	27	TOP2A	22.66667	23.66667	4.564785
P11387	31	24	28	TOP1	27.66667	28.66667	4.841302
P11310	5	8	3	ACADM	5.333333	6.333333	2.662965
P11177	11	11	11	PDHB	11	12	3.584963
P11172	7	7	8	UMPS	7.333333	8.333333	3.058894
P11166	1	1	1	SLC2A1	1	2	1
P11142	51	57	54	HSPA8	54	55	5.78136

P11117	7	4	6	ACP2	5.666667	6.666667	2.736966
P11021	24	29	26	HSPA5	26.33333	27.33333	4.77259
P10809	35	34	25	HSPD1	31.33333	32.33333	5.01495
P10644	2	3	2	PRKAR1A	2.333333	3.333333	1.736966
P10599	19	15	16	TXN	16.66667	17.66667	4.142958
P10586	7	5	13	PTPRF	8.333333	9.333333	3.222392
P10321	7	7	7	HLA-C	7	8	3
P10155	2	3	3	RO60	2.666667	3.666667	1.874469
P0DP25	1	1	1	CALM	1	2	1
P0DOX2	4	4	4	IGA2	4	5	2.321928
P0DN79	6	4	3	CBS	4.333333	5.333333	2.415037
P0DMV9	62	63	61	HSPA1	62	63	5.97728
P0CG08	6	4	5	GPR89B/GI	5	6	2.584963
P09874	49	43	47	PARP1	46.33333	47.33333	5.564785
P09661	12	10	9	SNRPA1	10.33333	11.33333	3.5025
P09651	27	23	28	HNRNPA1	26	27	4.754888
P09543	22	24	21	CNP	22.33333	23.33333	4.544321
P09429	4	6	4	HMGB1	4.666667	5.666667	2.5025
P09234	3	4	4	SNRPC	3.666667	4.666667	2.222392
P09110	1	2	2	ACAA1	1.666667	2.666667	1.415037
P09001	6	8	5	MRPL3	6.333333	7.333333	2.874469
P08865	9	6	13	RPSA	9.333333	10.33333	3.369234
P08754	2	2	2	GNAI3	2	3	1.584963
P08708	61	59	63	RPS17	61	62	5.954196
P08670	4	2	3	VIM	3	4	2
P08621	15	14	14	SNRNP70	14.33333	15.33333	3.938599
P08574	7	9	8	CYC1	8	9	3.169925
P08243	12	17	12	ASNS	13.66667	14.66667	3.874469
P08240	18	17	21	SRPRA	18.66667	19.66667	4.297681
P08238	40	46	37	HSP90AB1	41	42	5.392317
P08195	37	37	39	SLC3A2	37.66667	38.66667	5.273018
P07910	20	14	20	HNRNPC	18	19	4.247928
P07900	12	16	13	HSP90AA1	13.66667	14.66667	3.874469
P07814	44	45	42	EPRS1	43.66667	44.66667	5.481127
P07437	169	162	146	TUBB	159	160	7.321928
P07355	7	8	8	ANXA2	7.666667	8.666667	3.115477
P07305	4	5	5	H1-0	4.666667	5.666667	2.5025
P07195	3	1	2	LDHB	2	3	1.584963
P06748	144	123	112	NPM1	126.3333	127.3333	6.992466
P06730	4	3	4	EIF4E	3.666667	4.666667	2.222392
P06576	61	55	56	ATP5F1B	57.33333	58.33333	5.866249
P06493	14	16	14	CDK1	14.66667	15.66667	3.969626
P06280	1	2	3	GLA	2	3	1.584963
P05412	3	3	4	JUN	3.333333	4.333333	2.115477
P05388	67	64	64	RPLP0	65	66	6.044394
P05387	32	24	27	RPLP2	27.66667	28.66667	4.841302
P05386	17	12	16	RPLP1	15	16	4
P05141	72	63	67	SLC25A5	67.33333	68.33333	6.094518
P05023	41	46	42	ATP1A1	43	44	5.459432
P04844	36	42	39	RPN2	39	40	5.321928
P04843	43	46	44	RPN1	44.33333	45.33333	5.5025
P04637	2	3	3	TP53	2.666667	3.666667	1.874469
P04406	63	57	62	GAPDH	60.66667	61.66667	5.946419
P04350	140	138	130	TUBB4A	136	137	7.098032
P04264	29	26	39	KRT1	31.33333	32.33333	5.01495
P04183	1	1	2	TK1	1.333333	2.333333	1.222392

P04181	4	3	4	OAT	3.666667	4.666667	2.222392
P04114	2	1	2	APOB	1.666667	2.666667	1.415037
P04080	9	11	9	CSTB	9.666667	10.66667	3.415037
P04049	4	4	3	RAF1	3.666667	4.666667	2.222392
P04035	2	4	2	HMGCR	2.666667	3.666667	1.874469
P03928	6	3	4	ATP8	4.333333	5.333333	2.415037
P02786	5	5	4	TFRC	4.666667	5.666667	2.5025
P02768	5	4	5	ALB	4.666667	5.666667	2.5025
P00846	3	3	4	ATP6	3.333333	4.333333	2.115477
P00492	8	9	8	HPRT1	8.333333	9.333333	3.222392
P00403	5	7	6	COX2	6	7	2.807355
P00395	2	1	2	COX1	1.666667	2.666667	1.415037
P00390	4	3	2	GSR	3	4	2
O96019	1	3	2	ACTL6A	2	3	1.584963
O96005	2	2	2	CLPTM1	2	3	1.584963
O95905	6	5	8	ECD	6.333333	7.333333	2.874469
O95881	2	2	3	TXNDC12	2.333333	3.333333	1.736966
O95864	1	1	1	FADS2	1	2	1
O95831	13	18	19	AIFM1	16.66667	17.66667	4.142958
O95819	5	5	4	MAP4K4	4.666667	5.666667	2.5025
O95816	10	8	8	BAG2	8.666667	9.666667	3.273018
O95801	4	3	1	TTC4	2.666667	3.666667	1.874469
O95793	34	34	29	STAU1	32.33333	33.33333	5.058894
O95782	8	7	10	AP2A1	8.333333	9.333333	3.222392
O95707	2	3	4	POP4	3	4	2
O95674	3	4	3	CDS2	3.333333	4.333333	2.115477
O95573	14	15	14	ACSL3	14.33333	15.33333	3.938599
O95490	5	5	5	ADGRL2	5	6	2.584963
O95486	1	1	1	SEC24A	1	2	1
O95470	33	29	26	SGPL1	29.33333	30.33333	4.922832
O95433	3	3	1	AHSA1	2.333333	3.333333	1.736966
O95394	1	1	1	PGM3	1	2	1
O95373	54	54	57	IPO7	55	56	5.807355
O95372	2	1	1	LYPLA2	1.333333	2.333333	1.222392
O95363	9	8	8	FARS2	8.333333	9.333333	3.222392
O95347	22	25	23	SMC2	23.33333	24.33333	4.604862
O95299	2	2	2	NDUFA10	2	3	1.584963
O95248	5	7	3	SBF1	5	6	2.584963
O95232	24	22	21	LUC7L3	22.33333	23.33333	4.544321
O95218	3	2	5	ZRANB2	3.333333	4.333333	2.115477
O95197	1	3	3	RTN3	2.333333	3.333333	1.736966
O95163	5	5	4	ELP1	4.666667	5.666667	2.5025
O95159	3	2	3	ZFPL1	2.666667	3.666667	1.874469
O95071	27	22	24	UBR5	24.33333	25.33333	4.662965
O94966	4	4	4	USP19	4	5	2.321928
O94906	9	7	4	PRPF6	6.666667	7.666667	2.938599
O94905	5	6	4	ERLIN2	5	6	2.584963
O94874	10	9	6	UFL1	8.333333	9.333333	3.222392
O94826	1	1	1	TOMM70	1	2	1
O94822	7	10	9	LTN1	8.666667	9.666667	3.273018
O94813	14	11	14	SLIT2	13	14	3.807355
O76094	20	16	17	SRP72	17.66667	18.66667	4.222392
O76071	6	9	10	CIAO1	8.333333	9.333333	3.222392
O76021	25	21	24	RSL1D1	23.33333	24.33333	4.604862
O75976	3	2	2	CPD	2.333333	3.333333	1.736966
O75964	9	8	10	ATP5MG	9	10	3.321928

O75947	20	23	26	ATP5PD	23	24	4.584963
O75940	3	3	3	SMNDC1	3	4	2
O75934	10	12	12	BCAS2	11.33333	12.33333	3.624491
O75925	3	4	4	PIAS1	3.666667	4.666667	2.222392
O75915	7	4	4	ARL6IP5	5	6	2.584963
O75832	2	1	2	PSMD10	1.666667	2.666667	1.415037
O75818	5	6	5	RPP40	5.333333	6.333333	2.662965
O75794	2	2	2	CDC123	2	3	1.584963
O75787	7	5	4	ATP6AP2	5.333333	6.333333	2.662965
O75746	9	11	9	SLC25A12	9.666667	10.66667	3.415037
O75694	16	22	23	NUP155	20.33333	21.33333	4.415037
O75691	11	10	13	UTP20	11.33333	12.33333	3.624491
O75643	39	38	35	SNRNP200	37.33333	38.33333	5.260528
O75616	4	3	5	ERAL1	4	5	2.321928
O75607	2	3	3	NPM3	2.666667	3.666667	1.874469
O75592	4	6	6	MYCBP2	5.333333	6.333333	2.662965
O75569	5	6	6	PRKRA	5.666667	6.666667	2.736966
O75534	20	25	24	CSDE1	23	24	4.584963
O75533	20	20	17	SF3B1	19	20	4.321928
O75531	1	1	1	BANF1	1	2	1
O75530	1	2	2	EED	1.666667	2.666667	1.415037
O75489	10	8	8	NDUFS3	8.666667	9.666667	3.273018
O75477	2	2	3	ERLIN1	2.333333	3.333333	1.736966
O75475	19	22	21	PSIP1	20.66667	21.66667	4.437405
O75419	2	1	3	CDC45	2	3	1.584963
O75400	3	3	3	PRPF40A	3	4	2
O75396	8	9	10	SEC22B	9	10	3.321928
O75352	2	3	3	MPDU1	2.666667	3.666667	1.874469
O75306	7	7	6	NDUFS2	6.666667	7.666667	2.938599
O75251	6	5	6	NDUFS7	5.666667	6.666667	2.736966
O75190	2	3	3	DNAJB6	2.666667	3.666667	1.874469
O75179	15	14	15	ANKRD17	14.66667	15.66667	3.969626
O75170	3	5	5	PPP6R2	4.333333	5.333333	2.415037
O75155	29	30	33	CAND2	30.66667	31.66667	4.984893
O75131	1	1	1	CPNE3	1	2	1
O75027	3	3	4	ABCB7	3.333333	4.333333	2.115477
O60942	9	9	9	RNGTT	9	10	3.321928
O60884	32	31	34	DNAJA2	32.33333	33.33333	5.058894
O60841	6	5	9	EIF5B	6.666667	7.666667	2.938599
O60832	9	8	12	DKC1	9.666667	10.66667	3.415037
O60830	2	1	2	TIMM17B	1.666667	2.666667	1.415037
O60783	2	1	2	MRPS14	1.666667	2.666667	1.415037
O60762	16	16	16	DPM1	16	17	4.087463
O60725	5	6	3	ICMT	4.666667	5.666667	2.5025
O60716	5	3	3	CTNND1	3.666667	4.666667	2.222392
O60684	7	5	6	KPNA6	6	7	2.807355
O60678	3	3	2	PRMT3	2.666667	3.666667	1.874469
O60573	2	2	1	EIF4E2	1.666667	2.666667	1.415037
O60518	5	7	4	RANBP6	5.333333	6.333333	2.662965
O60506	31	35	30	SYNCRIP	32	33	5.044394
O60488	8	5	6	ACSL4	6.333333	7.333333	2.874469
O60476	3	1	1	MAN1A2	1.666667	2.666667	1.415037
O60341	4	4	5	KDM1A	4.333333	5.333333	2.415037
O60313	15	20	20	OPA1	18.33333	19.33333	4.273018
O60287	4	6	9	URB1	6.333333	7.333333	2.874469
O60264	19	19	16	SMARCA5	18	19	4.247928

O43837	9	10	8	IDH3B	9	10	3.321928
O43819	4	3	2	SCO2	3	4	2
O43813	5	6	4	LANCL1	5	6	2.584963
O43809	8	8	10	NUDT21	8.666667	9.666667	3.273018
O43808	2	2	4	SLC25A17	2.666667	3.666667	1.874469
O43795	2	4	4	MYO1B	3.333333	4.333333	2.115477
O43765	1	1	1	SGTA	1	2	1
O43759	1	1	1	SYNGR1	1	2	1
O43684	12	9	13	BUB3	11.33333	12.33333	3.624491
O43670	4	3	6	ZNF207	4.333333	5.333333	2.415037
O43663	20	20	20	PRC1	20	21	4.392317
O43660	3	4	5	PLRG1	4	5	2.321928
O43615	4	3	4	TIMM44	3.666667	4.666667	2.222392
O43592	21	29	27	XPOT	25.66667	26.66667	4.736966
O43583	3	2	3	DENR	2.666667	3.666667	1.874469
O43505	14	15	17	B4GAT1	15.33333	16.33333	4.029747
O43390	25	31	29	HNRNPR	28.33333	29.33333	4.874469
O43347	17	12	18	MSI1	15.66667	16.66667	4.058894
O43324	3	2	2	EEF1E1	2.333333	3.333333	1.736966
O43292	1	2	1	GPAA1	1.333333	2.333333	1.222392
O43290	6	8	8	SART1	7.333333	8.333333	3.058894
O43264	12	10	9	ZW10	10.33333	11.33333	3.5025
O43251	2	1	2	RBFOX2	1.666667	2.666667	1.415037
O43242	9	9	9	PSMD3	9	10	3.321928
O43175	53	54	57	PHGDH	54.66667	55.66667	5.798742
O43164	11	11	15	PJA2	12.33333	13.33333	3.736966
O43156	4	5	4	TTI1	4.333333	5.333333	2.415037
O43143	65	62	61	DHX15	62.66667	63.66667	5.992466
O15479	1	3	2	MAGEB2	2	3	1.584963
O15397	11	13	10	IPO8	11.33333	12.33333	3.624491
O15381	5	6	6	NVL	5.666667	6.666667	2.736966
O15371	6	8	5	EIF3D	6.333333	7.333333	2.874469
O15355	15	17	13	PPM1G	15	16	4
O15321	3	5	6	TM9SF1	4.666667	5.666667	2.5025
O15294	2	2	1	OGT	1.666667	2.666667	1.415037
O15270	3	4	4	SPTLC2	3.666667	4.666667	2.222392
O15269	4	4	5	SPTLC1	4.333333	5.333333	2.415037
O15260	8	10	8	SURF4	8.666667	9.666667	3.273018
O15235	7	4	4	MRPS12	5	6	2.584963
O15228	4	3	2	GNPAT	3	4	2
O15226	31	30	25	NKRF	28.66667	29.66667	4.890771
O15212	1	1	1	PFDN6	1	2	1
O15173	6	8	7	PGRMC2	7	8	3
O15127	3	5	5	SCAMP2	4.333333	5.333333	2.415037
O15084	19	17	18	ANKRD28	18	19	4.247928
O15067	3	4	3	PFAS	3.333333	4.333333	2.115477
O15042	20	16	17	U2SURP	17.66667	18.66667	4.222392
O15027	8	9	5	SEC16A	7.333333	8.333333	3.058894
O14981	6	8	5	BTAF1	6.333333	7.333333	2.874469
O14980	55	58	55	XPO1	56	57	5.83289
O14979	8	11	8	HNRNPDL	9	10	3.321928
O14966	3	3	3	RAB29	3	4	2
O14965	8	7	9	AURKA	8	9	3.169925
O14949	2	2	2	UQCRQ	2	3	1.584963
O14929	9	9	8	HAT1	8.666667	9.666667	3.273018
O14925	5	6	6	TIMM23	5.666667	6.666667	2.736966

O14893	13	14	13	GEMIN2	13.33333	14.33333	3.841302
O14874	1	1	1	BCKDK	1	2	1
O14828	10	5	6	SCAMP3	7	8	3
O14787	8	8	7	TNPO2	7.666667	8.666667	3.115477
O14744	4	5	5	PRMT5	4.666667	5.666667	2.5025
O14735	6	7	8	CDIPT	7	8	3
O14681	3	2	2	EI24	2.333333	3.333333	1.736966
O14654	42	41	47	IRS4	43.33333	44.33333	5.47032
O14579	14	13	7	COPE	11.33333	12.33333	3.624491
O00767	5	4	2	SCD	3.666667	4.666667	2.222392
O00743	21	21	18	PPP6C	20	21	4.392317
O00629	6	4	6	KPNA4	5.333333	6.333333	2.662965
O00571	67	64	54	DDX3X	61.66667	62.66667	5.969626
O00567	19	22	21	NOP56	20.66667	21.66667	4.437405
O00566	4	5	3	MPHOSPH1	4	5	2.321928
O00560	2	4	4	SDCBP	3.333333	4.333333	2.115477
O00541	12	9	14	PES1	11.66667	12.66667	3.662965
O00505	9	11	10	KPNA3	10	11	3.459432
O00487	8	6	7	PSMD14	7	8	3
O00483	7	8	9	NDUFA4	8	9	3.169925
O00471	3	2	4	EXOC5	3	4	2
O00442	11	9	9	RTCA	9.666667	10.66667	3.415037
O00425	24	26	28	IGF2BP3	26	27	4.754888
O00411	46	37	36	POLRMT	39.66667	40.66667	5.345775
O00410	38	41	40	IPO5	39.66667	40.66667	5.345775
O00400	1	2	1	SLC33A1	1.333333	2.333333	1.222392
O00264	24	20	24	PGRMC1	22.66667	23.66667	4.564785
O00233	3	2	3	PSMD9	2.666667	3.666667	1.874469
O00232	4	5	4	PSMD12	4.333333	5.333333	2.415037
O00231	5	5	5	PSMD11	5	6	2.584963
O00217	6	7	5	NDUFS8	6	7	2.807355
O00178	16	16	14	GTPBP1	15.33333	16.33333	4.029747
O00165	8	11	8	HAX1	9	10	3.321928
O00148	9	7	6	DDX39A	7.333333	8.333333	3.058894
O00139	18	19	17	KIF2A	18	19	4.247928
O00116	13	14	14	AGPS	13.66667	14.66667	3.874469
A6ZKI3	1	1	1	RTL8C	1	2	1
A6NDU8	1	1	1	RIMOC1	1	2	1
A5YKK6	1	5	8	CNOT1	4.666667	5.666667	2.5025
A4D1E9	12	13	14	GTPBP10	13	14	3.807355
A1L0T0	14	14	14	ILVBL	14	15	3.906891
A0FGR8	22	23	22	ESYT2	22.33333	23.33333	4.544321
A0A0B4J2D	1	2	2	GATD3B	1.666667	2.666667	1.415037
Q9Y6V7	6	6	8	DDX49	6.666667	7.666667	2.938599
Q9Y6C9	2	4	2	MTCH2	2.666667	3.666667	1.874469
Q9Y6A4	4	4	5	CFAP20	4.333333	5.333333	2.415037
Q9Y606	4	2	2	PUS1	2.666667	3.666667	1.874469
Q9Y5Z9	1	1	1	UBIAD1	1	2	1
Q9Y5J1	4	3	3	UTP18	3.333333	4.333333	2.115477
Q9Y5A7	1	2	1	NUB1	1.333333	2.333333	1.222392
Q9Y580	1	1	1	RBM7	1	2	1
Q9Y4I1	1	1	1	MYO5A	1	2	1
Q9Y4F3	1	1	1	MARF1	1	2	1
Q9Y4A5	7	3	4	TRRAP	4.666667	5.666667	2.5025
Q9Y3T9	6	4	6	NOC2L	5.333333	6.333333	2.662965
Q9Y3B9	4	4	4	RRP15	4	5	2.321928

Q9Y3B7	5	6	6	MRPL11	5.666667	6.666667	2.736966
Q9Y3B4	1	1	2	SF3B6	1.333333	2.333333	1.222392
Q9Y3A4	4	2	4	RRP7A	3.333333	4.333333	2.115477
Q9Y388	2	2	1	RBMX2	1.666667	2.666667	1.415037
Q9Y2X9	2	6	2	ZNF281	3.333333	4.333333	2.115477
Q9Y2V7	2	2	2	COG6	2	3	1.584963
Q9Y2R0	2	2	1	COA3	1.666667	2.666667	1.415037
Q9Y2Q5	4	2	1	LAMTOR2	2.333333	3.333333	1.736966
Q9Y2Q3	3	1	2	GSTK1	2	3	1.584963
Q9Y2A7	1	2	1	NCKAP1	1.333333	2.333333	1.222392
Q9Y291	3	4	3	MRPS33	3.333333	4.333333	2.115477
Q9Y277	2	3	3	VDAC3	2.666667	3.666667	1.874469
Q9UQE7	3	5	3	SMC3	3.666667	4.666667	2.222392
Q9UMQ6	1	1	1	CAPN11	1	2	1
Q9ULT8	3	1	3	HECTD1	2.333333	3.333333	1.736966
Q9ULH0	2	1	1	KIDINS220	1.333333	2.333333	1.222392
Q9UL40	3	1	3	ZNF346	2.333333	3.333333	1.736966
Q9UL15	1	2	3	BAG5	2	3	1.584963
Q9UKM9	2	2	1	RALY	1.666667	2.666667	1.415037
Q9UKJ3	1	1	1	GPATCH8	1	2	1
Q9UKD2	6	4	5	MRTO4	5	6	2.584963
Q9UJX4	3	4	2	ANAPC5	3	4	2
Q9UJX3	4	2	1	ANAPC7	2.333333	3.333333	1.736966
Q9UJK0	1	2	1	TSR3	1.333333	2.333333	1.222392
Q9UJA5	2	2	3	TRMT6	2.333333	3.333333	1.736966
Q9UIW2	1	1	2	PLXNA1	1.333333	2.333333	1.222392
Q9UI43	2	1	1	MRM2	1.333333	2.333333	1.222392
Q9UHK0	3	2	1	NUFIP1	2	3	1.584963
Q9UHA4	1	1	1	LAMTOR3	1	2	1
Q9UGU5	2	1	1	HMGXB4	1.333333	2.333333	1.222392
Q9UGJ1	1	4	3	TUBGCP4	2.666667	3.666667	1.874469
Q9UFW8	3	2	3	CGGBP1	2.666667	3.666667	1.874469
Q9UER7	1	2	2	DAXX	1.666667	2.666667	1.415037
Q9UBW7	2	4	3	ZMYM2	3	4	2
Q9UBU9	7	6	6	NXF1	6.333333	7.333333	2.874469
Q9P2W9	1	1	1	STX18	1	2	1
Q9P2K5	2	2	2	MYEF2	2	3	1.584963
Q9P289	4	3	3	STK26	3.333333	4.333333	2.115477
Q9NZQ3	1	1	1	NCKIPSD	1	2	1
Q9NZJ5	1	1	1	EIF2AK3	1	2	1
Q9NYV4	4	4	4	CDK12	4	5	2.321928
Q9NY93	5	6	6	DDX56	5.666667	6.666667	2.736966
Q9NY61	1	1	1	AATF	1	2	1
Q9NXS2	3	2	3	QPCTL	2.666667	3.666667	1.874469
Q9NXB9	2	2	1	ELOVL2	1.666667	2.666667	1.415037
Q9NX74	1	2	2	DUS2	1.666667	2.666667	1.415037
Q9NX40	1	2	2	OCIAD1	1.666667	2.666667	1.415037
Q9NWU5	1	5	2	MRPL22	2.666667	3.666667	1.874469
Q9NWT8	1	1	1	AURKAIP1	1	2	1
Q9NWT1	3	5	6	PAK1IP1	4.666667	5.666667	2.5025
Q9NWK9	1	2	2	ZNHIT6	1.666667	2.666667	1.415037
Q9NW75	1	1	2	GPATCH2	1.333333	2.333333	1.222392
Q9NVV4	4	6	4	MTPAP	4.666667	5.666667	2.5025
Q9NVU7	2	4	7	SDAD1	4.333333	5.333333	2.415037
Q9NVU0	1	3	2	POLR3E	2	3	1.584963
Q9NVS2	2	3	3	MRPS18A	2.666667	3.666667	1.874469

Q9NV96	1	1	1	TMEM30A	1	2	1
Q9NV56	1	1	1	MRGBP	1	2	1
Q9NV31	1	1	2	IMP3	1.333333	2.333333	1.222392
Q9NUN5	1	1	1	LMBRD1	1	2	1
Q9NUM4	2	1	2	TMEM106I	1.666667	2.666667	1.415037
Q9NUL7	12	9	11	DDX28	10.66667	11.66667	3.544321
Q9NUL3	6	9	11	STAU2	8.666667	9.666667	3.273018
Q9NTJ5	1	1	1	SACM1L	1	2	1
Q9NT62	2	1	2	ATG3	1.666667	2.666667	1.415037
Q9NSP4	1	1	1	CENPM	1	2	1
Q9NSI2	10	9	8	SLX9	9	10	3.321928
Q9NSG2	3	2	2	FIRRM	2.333333	3.333333	1.736966
Q9NRX1	6	3	4	PNO1	4.333333	5.333333	2.415037
Q9NRP0	3	1	2	OSTC	2	3	1.584963
Q9NRM2	2	2	3	ZNF277	2.333333	3.333333	1.736966
Q9NRA8	3	1	2	EIF4ENIF1	2	3	1.584963
Q9NQZ2	3	4	3	UTP3	3.333333	4.333333	2.115477
Q9NQW6	1	1	1	ANLN	1	2	1
Q9NQC8	1	1	1	IFT46	1	2	1
Q9NQ50	2	1	2	MRPL40	1.666667	2.666667	1.415037
Q9NPL8	1	1	1	TIMMDC1	1	2	1
Q9NPF5	1	1	2	DMAP1	1.333333	2.333333	1.222392
Q9NP97	2	2	2	DYNLRB1	2	3	1.584963
Q9NP92	2	3	3	MRPS30	2.666667	3.666667	1.874469
Q9NP50	2	3	2	SINHCAF	2.333333	3.333333	1.736966
Q9HDC5	2	3	3	JPH1	2.666667	3.666667	1.874469
Q9HC56	1	2	2	PCDH9	1.666667	2.666667	1.415037
Q9HB90	1	2	2	RRAGC	1.666667	2.666667	1.415037
Q9HAS0	2	3	1	C17orf75	2	3	1.584963
Q9HAD4	1	2	1	WDR41	1.333333	2.333333	1.222392
Q9H9V9	3	1	2	JMJD4	2	3	1.584963
Q9H9T3	2	1	1	ELP3	1.333333	2.333333	1.222392
Q9H9L3	4	3	2	ISG20L2	3	4	2
Q9H900	1	1	1	ZWILCH	1	2	1
Q9H8Y5	6	7	3	ANKZF1	5.333333	6.333333	2.662965
Q9H8M2	2	2	2	BRD9	2	3	1.584963
Q9H8G2	1	2	2	CAAP1	1.666667	2.666667	1.415037
Q9H869	4	3	2	YY1AP1	3	4	2
Q9H845	3	2	4	ACAD9	3	4	2
Q9H814	1	1	1	PHAX	1	2	1
Q9H7H0	7	6	5	METTL17	6	7	2.807355
Q9H7E2	1	1	1	TDRD3	1	2	1
Q9H6Y2	1	1	1	WDR55	1	2	1
Q9H649	3	3	5	NSUN3	3.666667	4.666667	2.222392
Q9H5X1	2	2	3	CIAO2A	2.333333	3.333333	1.736966
Q9H4L7	2	1	2	SMARCAD1	1.666667	2.666667	1.415037
Q9H4L4	2	2	1	SENP3	1.666667	2.666667	1.415037
Q9H3G5	1	1	1	CPVL	1	2	1
Q9H2W6	3	2	4	MRPL46	3	4	2
Q9H2P0	1	1	2	ADNP	1.333333	2.333333	1.222392
Q9H2M9	1	1	2	RAB3GAP2	1.333333	2.333333	1.222392
Q9H1X3	2	4	3	DNAJC25	3	4	2
Q9H1B5	1	1	1	XYLT2	1	2	1
Q9H0U6	1	1	1	MRPL18	1	2	1
Q9H089	10	8	8	LSG1	8.666667	9.666667	3.273018
Q9GZS1	4	2	2	POLR1E	2.666667	3.666667	1.874469

Q9GZR2	1	2	1	REXO4	1.333333	2.333333	1.222392
Q9GZP9	6	6	6	DERL2	6	7	2.807355
Q9GZM5	1	2	2	YIPF3	1.666667	2.666667	1.415037
Q9GZL7	2	2	4	WDR12	2.666667	3.666667	1.874469
Q9C0H9	1	1	1	SRCIN1	1	2	1
Q9BZ95	2	2	2	NSD3	2	3	1.584963
Q9BYK8	2	2	1	HELZ2	1.666667	2.666667	1.415037
Q9BYI3	1	1	1	HYCC1	1	2	1
Q9BYG3	5	4	5	NIFK	4.666667	5.666667	2.5025
Q9BYD1	2	1	1	MRPL13	1.333333	2.333333	1.222392
Q9BY44	3	3	3	EIF2A	3	4	2
Q9BXS6	7	5	4	NUSAP1	5.333333	6.333333	2.662965
Q9BXS5	2	2	3	AP1M1	2.333333	3.333333	1.736966
Q9BXP5	3	2	3	SRRT	2.666667	3.666667	1.874469
Q9BX10	3	2	1	GTPBP2	2	3	1.584963
Q9BWN1	1	1	1	PRR14	1	2	1
Q9BW83	3	2	3	IFT27	2.666667	3.666667	1.874469
Q9BW72	3	1	1	HIGD2A	1.666667	2.666667	1.415037
Q9BW61	1	1	2	DDA1	1.333333	2.333333	1.222392
Q9BVK8	1	2	3	TMEM147	2	3	1.584963
Q9BVJ6	9	6	7	UTP14A	7.333333	8.333333	3.058894
Q9BVC6	1	1	1	TMEM109	1	2	1
Q9BUL9	1	1	1	RPP25	1	2	1
Q9BUK6	2	1	2	MSTO1	1.666667	2.666667	1.415037
Q9BU61	1	1	2	NDUFAF3	1.333333	2.333333	1.222392
Q9BT17	3	2	1	MTG1	2	3	1.584963
Q9BSJ2	2	2	2	TUBGCP2	2	3	1.584963
Q9BSC4	1	4	3	NOL10	2.666667	3.666667	1.874469
Q9BRZ2	5	5	4	TRIM56	4.666667	5.666667	2.5025
Q9BRT6	2	2	2	LLPH	2	3	1.584963
Q9BRS2	1	1	3	RIOK1	1.666667	2.666667	1.415037
Q9BRJ2	3	1	2	MRPL45	2	3	1.584963
Q9BQ04	17	20	21	RBM4B	19.33333	20.33333	4.345775
Q99956	3	3	5	DUSP9	3.666667	4.666667	2.222392
Q99943	2	2	2	AGPAT1	2	3	1.584963
Q99741	1	1	2	CDC6	1.333333	2.333333	1.222392
Q99570	2	2	1	PIK3R4	1.666667	2.666667	1.415037
Q99547	1	1	1	MPHOSPH(	1	2	1
Q99523	1	2	1	SORT1	1.333333	2.333333	1.222392
Q96TC7	4	3	2	RMDN3	3	4	2
Q96T88	4	3	2	UHRF1	3	4	2
Q96SK2	1	2	1	TMEM209	1.333333	2.333333	1.222392
Q96SI9	6	12	5	STRBP	7.666667	8.666667	3.115477
Q96S44	2	1	2	TP53RK	1.666667	2.666667	1.415037
Q96QR8	8	10	10	PURB	9.333333	10.33333	3.369234
Q96QE3	3	2	2	ATAD5	2.333333	3.333333	1.736966
Q96NB2	2	1	2	SFXN2	1.666667	2.666667	1.415037
Q96N66	1	1	1	MBOAT7	1	2	1
Q96MX3	2	3	1	ZNF48	2	3	1.584963
Q96MU7	2	1	2	YTHDC1	1.666667	2.666667	1.415037
Q96LL9	2	2	2	DNAJC30	2	3	1.584963
Q96L58	3	2	2	B3GALT6	2.333333	3.333333	1.736966
Q96K58	2	4	3	ZNF668	3	4	2
Q96JK2	1	2	1	DCAF5	1.333333	2.333333	1.222392
Q96JG8	5	7	3	MAGED4	5	6	2.584963
Q96JB5	2	2	2	CDK5RAP3	2	3	1.584963

Q96I25	4	6	7	RBM17	5.666667	6.666667	2.736966
Q96HY6	2	2	3	DDRGK1	2.333333	3.333333	1.736966
Q96HQ2	1	2	1	CDKN2AIPI	1.333333	2.333333	1.222392
Q96HE9	4	5	4	PRR11	4.333333	5.333333	2.415037
Q96HC4	1	1	1	PDLIM5	1	2	1
Q96GY0	2	2	4	ZC2HC1A	2.666667	3.666667	1.874469
Q96GR4	1	1	1	ZDHHC12	1	2	1
Q96GD4	1	2	1	AURKB	1.333333	2.333333	1.222392
Q96GC5	2	2	2	MRPL48	2	3	1.584963
Q96FX7	1	1	1	TRMT61A	1	2	1
Q96EC8	2	1	1	YIPF6	1.333333	2.333333	1.222392
Q96EB6	4	2	3	SIRT1	3	4	2
Q96DY7	1	1	1	MTBP	1	2	1
Q96DX4	1	1	1	RSPRY1	1	2	1
Q96DV4	3	3	4	MRPL38	3.333333	4.333333	2.115477
Q96D70	2	2	2	R3HDM4	2	3	1.584963
Q96CX2	1	1	2	KCTD12	1.333333	2.333333	1.222392
Q96C57	1	1	1	C12orf43	1	2	1
Q96BR1	1	1	1	SGK3	1	2	1
Q96B26	5	5	5	EXOSC8	5	6	2.584963
Q96AX1	1	2	1	VPS33A	1.333333	2.333333	1.222392
Q96AQ6	6	5	9	PBXIP1	6.666667	7.666667	2.938599
Q96AE7	1	1	1	TTC17	1	2	1
Q96A35	3	4	3	MRPL24	3.333333	4.333333	2.115477
Q969H6	1	1	1	POP5	1	2	1
Q92979	4	2	2	EMG1	2.666667	3.666667	1.874469
Q92903	1	1	1	CDS1	1	2	1
Q92901	7	10	7	RPL3L	8	9	3.169925
Q92879	1	2	2	CELF1	1.666667	2.666667	1.415037
Q92688	3	3	3	ANP32B	3	4	2
Q92544	1	2	3	TM9SF4	2	3	1.584963
Q8WY36	3	1	2	BBX	2	3	1.584963
Q8WXH0	2	2	3	SYNE2	2.333333	3.333333	1.736966
Q8WXA9	1	1	2	SREK1	1.333333	2.333333	1.222392
Q8WX92	2	3	2	NELFB	2.333333	3.333333	1.736966
Q8WVQ1	1	1	1	CANT1	1	2	1
Q8WVM8	2	1	2	SCFD1	1.666667	2.666667	1.415037
Q8WVC6	1	1	1	DCAKD	1	2	1
Q8WVC0	2	3	2	LEO1	2.333333	3.333333	1.736966
Q8WUY9	1	1	1	DEPDC1B	1	2	1
Q8WUY8	2	1	1	NAT14	1.333333	2.333333	1.222392
Q8WUK0	2	2	4	PTPMT1	2.666667	3.666667	1.874469
Q8WUB8	3	2	3	PHF10	2.666667	3.666667	1.874469
Q8WUA2	2	3	3	PPIL4	2.666667	3.666667	1.874469
Q8WU76	1	2	2	SCFD2	1.666667	2.666667	1.415037
Q8WU67	1	2	1	ABHD3	1.333333	2.333333	1.222392
Q8WTT2	5	7	6	NOC3L	6	7	2.807355
Q8TED0	7	7	5	UTP15	6.333333	7.333333	2.874469
Q8TCT9	4	2	3	HM13	3	4	2
Q8TBM8	2	1	1	DNAJB14	1.333333	2.333333	1.222392
Q8TBF4	2	1	1	ZCRB1	1.333333	2.333333	1.222392
Q8TB61	3	3	4	SLC35B2	3.333333	4.333333	2.115477
Q8TAF3	1	1	1	WDR48	1	2	1
Q8TAE8	2	1	1	GADD45GI	1.333333	2.333333	1.222392
Q8NI36	8	7	9	WDR36	8	9	3.169925
Q8NFI3	1	2	1	ENGASE	1.333333	2.333333	1.222392

Q8NCX0	1	1	1	CCDC150	1	2	1
Q8NCR0	1	1	3	B3GALNT2	1.666667	2.666667	1.415037
Q8NCH0	2	1	2	CHST14	1.666667	2.666667	1.415037
Q8NCD3	3	1	1	HJURP	1.666667	2.666667	1.415037
Q8NC54	1	1	1	C5orf15	1	2	1
Q8NBZ7	1	1	1	UXS1	1	2	1
Q8NBT0	2	1	1	POC1A	1.333333	2.333333	1.222392
Q8NB37	1	1	1	GATD1	1	2	1
Q8N9N2	2	2	2	ASCC1	2	3	1.584963
Q8N999	1	1	1	C12orf29	1	2	1
Q8N8L6	1	1	1	ARL10	1	2	1
Q8N8D1	1	1	1	PDCD7	1	2	1
Q8N766	3	4	2	EMC1	3	4	2
Q8N6M0	4	3	4	OTUD6B	3.666667	4.666667	2.222392
Q8N5U6	7	6	7	RNF10	6.666667	7.666667	2.938599
Q8N5P1	2	2	5	ZC3H8	3	4	2
Q8N5A5	1	3	2	ZGPAT	2	3	1.584963
Q8N4V1	1	1	1	MMGT1	1	2	1
Q8N442	1	1	1	GUF1	1	2	1
Q8N441	1	1	1	FGFRL1	1	2	1
Q8N1G4	3	4	7	LRRC47	4.666667	5.666667	2.5025
Q8N138	1	1	1	ORMDL3	1	2	1
Q8N0V3	8	8	9	RBFA	8.333333	9.333333	3.222392
Q8N0U8	1	3	1	VKORC1L1	1.666667	2.666667	1.415037
Q8IZU8	1	1	1	DSEL	1	2	1
Q8IZ07	1	1	2	ANKRD13A	1.333333	2.333333	1.222392
Q8IYW2	1	1	1	CFAP46	1	2	1
Q8IYU8	4	3	3	MICU2	3.333333	4.333333	2.115477
Q8IYB3	1	2	1	SRRM1	1.333333	2.333333	1.222392
Q8IY18	3	2	1	SMC5	2	3	1.584963
Q8IXU6	1	1	2	SLC35F2	1.333333	2.333333	1.222392
Q8IXK0	1	2	2	PHC2	1.666667	2.666667	1.415037
Q8IX18	2	1	4	DHX40	2.333333	3.333333	1.736966
Q8IX01	1	1	1	SUGP2	1	2	1
Q8IWT6	2	3	1	LRRC8A	2	3	1.584963
Q8IWR0	7	6	7	ZC3H7A	6.666667	7.666667	2.938599
Q8IVS2	4	1	1	MCAT	2	3	1.584963
Q8IV48	3	1	1	ERI1	1.666667	2.666667	1.415037
Q86YV9	1	1	1	HPS6	1	2	1
Q86Y79	4	3	2	PTRH1	3	4	2
Q86XZ4	2	3	3	SPATS2	2.666667	3.666667	1.874469
Q86XX4	1	1	1	FRAS1	1	2	1
Q86XR2	2	2	2	NIBAN3	2	3	1.584963
Q86XL3	2	1	1	ANKLE2	1.333333	2.333333	1.222392
Q86WJ1	6	5	2	CHD1L	4.333333	5.333333	2.415037
Q86W42	1	2	1	THOC6	1.333333	2.333333	1.222392
Q86VU5	2	1	2	COMTD1	1.666667	2.666667	1.415037
Q86V59	5	6	4	PNMA8A	5	6	2.584963
Q86UY6	5	7	7	NAA40	6.333333	7.333333	2.874469
Q86U06	9	7	8	RBM23	8	9	3.169925
Q86TN4	1	1	1	TRPT1	1	2	1
Q86TJ2	1	2	1	TADA2B	1.333333	2.333333	1.222392
Q7Z4W1	1	1	2	DCXR	1.333333	2.333333	1.222392
Q7Z460	3	7	6	CLASP1	5.333333	6.333333	2.662965
Q7Z434	1	1	1	MAVS	1	2	1
Q7Z333	4	3	6	SETX	4.333333	5.333333	2.415037

Q7Z2K6	1	1	1	ERMP1	1	2	1
Q7RTS9	1	1	1	DYM	1	2	1
Q7L5N7	2	2	5	LPCAT2	3	4	2
Q7L576	2	3	3	CYFIP1	2.666667	3.666667	1.874469
Q7L1Q6	3	3	5	BZW1	3.666667	4.666667	2.222392
Q7KZI7	3	3	4	MARK2	3.333333	4.333333	2.115477
Q7KYR7	1	1	2	BTN2A1	1.333333	2.333333	1.222392
Q71UM5	9	11	11	RPS27L	10.33333	11.33333	3.5025
Q71UI9	10	9	8	H2AZ2	9	10	3.321928
Q6ZW31	1	1	1	SYDE1	1	2	1
Q6ZRS2	1	3	2	SRCAP	2	3	1.584963
Q6ZN55	1	2	1	ZNF574	1.333333	2.333333	1.222392
Q6ZMG9	1	1	1	CERS6	1	2	1
Q6UVY6	1	1	1	MOXD1	1	2	1
Q6RFH5	1	1	1	WDR74	1	2	1
Q6PL24	1	1	1	TMED8	1	2	1
Q6PJI9	1	1	4	WDR59	2	3	1.584963
Q6PEV8	1	2	1	FAM199X	1.333333	2.333333	1.222392
Q6PD62	9	11	8	CTR9	9.333333	10.33333	3.369234
Q6P5Z2	3	1	5	PKN3	3	4	2
Q6P1X5	5	3	5	TAF2	4.333333	5.333333	2.415037
Q6P1Q0	2	1	2	LETMD1	1.666667	2.666667	1.415037
Q6P087	4	2	3	RPUSD3	3	4	2
Q6NZY4	4	3	4	ZCCHC8	3.666667	4.666667	2.222392
Q6NZ67	1	1	1	MZT2B	1	2	1
Q6NXT1	1	1	1	ANKRD54	1	2	1
Q6NW34	2	2	4	NEPRO	2.666667	3.666667	1.874469
Q6NSJ5	2	1	1	LRRC8E	1.333333	2.333333	1.222392
Q6IN84	2	1	1	MRM1	1.333333	2.333333	1.222392
Q6GPH6	1	1	1	ITPRIPL1	1	2	1
Q6DCA0	1	1	1	AMMECR1	1	2	1
Q6BDS2	2	1	2	BLTP3A	1.666667	2.666667	1.415037
Q659A1	1	2	1	ICE2	1.333333	2.333333	1.222392
Q5VZE5	1	2	1	NAA35	1.333333	2.333333	1.222392
Q5VYS8	5	3	4	TUT7	4	5	2.321928
Q5VWZ2	1	1	1	LYPLAL1	1	2	1
Q5VW36	2	4	5	FOCAD	3.666667	4.666667	2.222392
Q5VTE6	1	1	1	ANGEL2	1	2	1
Q5VT66	1	2	1	MTARC1	1.333333	2.333333	1.222392
Q5TAQ9	1	4	3	DCAF8	2.666667	3.666667	1.874469
Q5T653	1	1	1	MRPL2	1	2	1
Q5T447	1	2	1	HECTD3	1.333333	2.333333	1.222392
Q5ST30	2	2	2	VARS2	2	3	1.584963
Q5RKV6	6	7	5	EXOSC6	6	7	2.807355
Q5JVS0	1	2	1	HABP4	1.333333	2.333333	1.222392
Q5JTW2	10	9	6	CEP78	8.333333	9.333333	3.222392
Q5H8A4	1	1	1	PIGG	1	2	1
Q5F1R6	3	3	3	DNAJC21	3	4	2
Q5BJH7	1	1	1	YIF1B	1	2	1
Q53HI1	1	1	2	UNC50	1.333333	2.333333	1.222392
Q53GL0	2	1	1	PLEKHO1	1.333333	2.333333	1.222392
Q4KMP7	3	1	3	TBC1D10B	2.333333	3.333333	1.736966
Q4G148	1	1	1	GXYLT1	1	2	1
Q4G0J3	5	6	3	LARP7	4.666667	5.666667	2.5025
Q3KP31	2	1	2	ZNF791	1.666667	2.666667	1.415037
Q2TB18	2	4	2	ASTE1	2.666667	3.666667	1.874469

Q27J81	1	1	1	INF2	1	2	1
Q1ED39	2	2	3	KNOP1	2.333333	3.333333	1.736966
Q16880	2	2	2	UGT8	2	3	1.584963
Q16644	1	1	1	MAPKAPK3	1	2	1
Q16543	1	2	2	CDC37	1.666667	2.666667	1.415037
Q16394	4	2	5	EXT1	3.666667	4.666667	2.222392
Q15910	1	1	1	EZH2	1	2	1
Q15776	5	4	5	ZKSCAN8	4.666667	5.666667	2.5025
Q15650	3	4	5	TRIP4	4	5	2.321928
Q15649	2	1	1	ZNHIT3	1.333333	2.333333	1.222392
Q15554	1	2	1	TERF2	1.333333	2.333333	1.222392
Q15527	2	2	1	SURF2	1.666667	2.666667	1.415037
Q15397	3	6	4	PUM3	4.333333	5.333333	2.415037
Q15165	1	1	1	PON2	1	2	1
Q15125	1	1	1	EBP	1	2	1
Q15048	1	2	1	LRRC14	1.333333	2.333333	1.222392
Q15031	1	1	1	LARS2	1	2	1
Q15022	2	2	3	SUZ12	2.333333	3.333333	1.736966
Q14CB8	2	1	2	ARHGAP19	1.666667	2.666667	1.415037
Q149N8	2	1	5	SHPRH	2.666667	3.666667	1.874469
Q14999	1	1	1	CUL7	1	2	1
Q14692	4	2	3	BMS1	3	4	2
Q14674	2	1	1	ESPL1	1.333333	2.333333	1.222392
Q14562	7	5	7	DHX8	6.333333	7.333333	2.874469
Q14558	4	1	1	PRPSAP1	2	3	1.584963
Q14331	1	1	1	FRG1	1	2	1
Q14240	12	9	14	EIF4A2	11.66667	12.66667	3.662965
Q14166	1	1	1	TTLL12	1	2	1
Q14152	1	1	3	EIF3A	1.666667	2.666667	1.415037
Q14146	1	4	2	URB2	2.333333	3.333333	1.736966
Q14012	1	1	1	CAMK1	1	2	1
Q14011	1	1	1	CIRBP	1	2	1
Q13901	1	1	1	C1D	1	2	1
Q13888	1	1	1	GTF2H2	1	2	1
Q13685	1	1	1	AAMP	1	2	1
Q13620	1	1	1	CUL4B	1	2	1
Q13601	15	15	12	KRR1	14	15	3.906891
Q13557	2	3	3	CAMK2D	2.666667	3.666667	1.874469
Q13362	3	2	3	PPP2R5C	2.666667	3.666667	1.874469
Q13257	1	2	1	MAD2L1	1.333333	2.333333	1.222392
Q13232	2	1	1	NME3	1.333333	2.333333	1.222392
Q13162	11	9	12	PRDX4	10.66667	11.66667	3.544321
Q13084	4	4	3	MRPL28	3.666667	4.666667	2.222392
Q13043	2	2	2	STK4	2	3	1.584963
Q13042	1	3	4	CDC16	2.666667	3.666667	1.874469
Q12986	1	1	1	NFX1	1	2	1
Q12931	3	2	2	TRAP1	2.333333	3.333333	1.736966
Q12873	5	7	5	CHD3	5.666667	6.666667	2.736966
Q12872	5	2	3	SFSWAP	3.333333	4.333333	2.115477
Q12788	11	8	10	TBL3	9.666667	10.66667	3.415037
Q10472	5	8	2	GALNT1	5	6	2.584963
Q0VDF9	2	3	1	HSPA14	2	3	1.584963
Q08431	1	1	1	MFGE8	1	2	1
Q04917	4	3	2	YWHAH	3	4	2
Q03701	3	4	5	CEBPZ	4	5	2.321928
Q02809	2	1	1	PLOD1	1.333333	2.333333	1.222392

Q01804	1	2	1	OTUD4	1.333333	2.333333	1.222392
Q00537	2	2	1	CDK17	1.666667	2.666667	1.415037
P98179	4	1	2	RBM3	2.333333	3.333333	1.736966
P84095	2	3	2	RHOG	2.333333	3.333333	1.736966
P83111	2	1	3	LACTB	2	3	1.584963
P82912	3	3	4	MRPS11	3.333333	4.333333	2.115477
P82664	6	6	7	MRPS10	6.333333	7.333333	2.874469
P80188	1	2	1	LCN2	1.333333	2.333333	1.222392
P78549	3	2	2	NTHL1	2.333333	3.333333	1.736966
P63220	3	5	3	RPS21	3.666667	4.666667	2.222392
P63096	2	2	2	GNAI1	2	3	1.584963
P62834	3	3	2	RAP1A	2.666667	3.666667	1.874469
P62310	2	1	1	LSM3	1.333333	2.333333	1.222392
P62306	2	3	3	SNRPF	2.666667	3.666667	1.874469
P62136	19	24	24	PPP1CA	22.33333	23.33333	4.544321
P61981	7	4	2	YWHAG	4.333333	5.333333	2.415037
P61221	4	3	4	ABCE1	3.666667	4.666667	2.222392
P60763	2	4	3	RAC3	3	4	2
P60228	3	2	3	EIF3E	2.666667	3.666667	1.874469
P58511	1	1	1	SMIM11	1	2	1
P56962	3	1	2	STX17	2	3	1.584963
P56378	1	1	2	ATP5MJ	1.333333	2.333333	1.222392
P56282	3	1	1	POLE2	1.666667	2.666667	1.415037
P54132	2	3	1	BLM	2	3	1.584963
P52756	1	3	2	RBM5	2	3	1.584963
P52298	1	1	1	NCBP2	1	2	1
P51809	1	1	1	VAMP7	1	2	1
P51151	2	2	1	RAB9A	1.666667	2.666667	1.415037
P50750	3	4	2	CDK9	3	4	2
P50579	2	2	2	METAP2	2	3	1.584963
P50452	2	2	2	SERPINB8	2	3	1.584963
P49841	1	1	2	GSK3B	1.333333	2.333333	1.222392
P49755	5	3	4	TMED10	4	5	2.321928
P49643	1	1	1	PRIM2	1	2	1
P49591	2	1	2	SARS1	1.666667	2.666667	1.415037
P49406	2	3	2	MRPL19	2.333333	3.333333	1.736966
P49069	1	1	1	CAMLG	1	2	1
P49023	1	1	1	PXN	1	2	1
P49006	1	1	1	MARCKSL1	1	2	1
P48995	1	1	1	TRPC1	1	2	1
P48730	7	7	9	CSNK1D	7.666667	8.666667	3.115477
P48729	2	5	6	CSNK1A1	4.333333	5.333333	2.415037
P48059	1	1	3	LIMS1	1.666667	2.666667	1.415037
P46821	2	1	1	MAP1B	1.333333	2.333333	1.222392
P46459	1	3	3	NSF	2.333333	3.333333	1.736966
P46013	5	1	2	MKI67	2.666667	3.666667	1.874469
P43487	1	2	3	RANBP1	2	3	1.584963
P42898	1	2	2	MTHFR	1.666667	2.666667	1.415037
P42696	8	7	8	RBM34	7.666667	8.666667	3.115477
P42694	2	4	2	HELZ	2.666667	3.666667	1.874469
P40855	2	1	2	PEX19	1.666667	2.666667	1.415037
P38432	1	1	1	COIL	1	2	1
P35241	3	3	3	RDX	3	4	2
P30566	1	1	1	ADSL	1	2	1
P29597	1	1	1	TYK2	1	2	1
P29558	2	1	2	RBMS1	1.666667	2.666667	1.415037

P28074	1	1	1	PSMB5	1	2	1
P27695	1	1	1	APEX1	1	2	1
P27448	4	2	4	MARK3	3.333333	4.333333	2.115477
P26639	3	1	5	TARS1	3	4	2
P26358	2	2	2	DNMT1	2	3	1.584963
P25786	1	1	1	PSMA1	1	2	1
P24534	3	2	2	EEF1B2	2.333333	3.333333	1.736966
P23588	1	1	1	EIF4B	1	2	1
P23526	1	3	4	AHCY	2.666667	3.666667	1.874469
P23458	3	3	1	JAK1	2.333333	3.333333	1.736966
P23378	1	1	3	GLDC	1.666667	2.666667	1.415037
P23258	1	2	1	TUBG1	1.333333	2.333333	1.222392
P22694	1	1	1	PRKACB	1	2	1
P22413	1	1	1	ENPP1	1	2	1
P20290	1	1	2	BTF3	1.333333	2.333333	1.222392
P20042	7	11	9	EIF2S2	9	10	3.321928
P19838	1	1	1	NFKB1	1	2	1
P17948	3	2	4	FLT1	3	4	2
P17028	2	2	1	ZNF24	1.666667	2.666667	1.415037
P16989	7	8	8	YBX3	7.666667	8.666667	3.115477
P16435	1	2	2	POR	1.666667	2.666667	1.415037
P15170	2	1	2	GSPT1	1.666667	2.666667	1.415037
P14406	2	2	1	COX7A2	1.666667	2.666667	1.415037
P14314	1	2	1	PRKCSH	1.333333	2.333333	1.222392
P13861	2	2	5	PRKAR2A	3	4	2
P13473	2	2	1	LAMP2	1.666667	2.666667	1.415037
P13073	1	1	1	COX4I1	1	2	1
P12814	1	1	1	ACTN1	1	2	1
P11532	1	1	1	DMD	1	2	1
P09601	2	2	3	HMOX1	2.333333	3.333333	1.736966
P09012	6	5	3	SNRPA	4.666667	5.666667	2.5025
P07947	2	1	2	YES1	1.666667	2.666667	1.415037
P07741	3	3	2	APRT	2.666667	3.666667	1.874469
P07237	2	1	1	P4HB	1.333333	2.333333	1.222392
P05455	4	3	3	SSB	3.333333	4.333333	2.115477
P05198	4	3	5	EIF2S1	4	5	2.321928
P05091	1	2	1	ALDH2	1.333333	2.333333	1.222392
P04792	2	2	2	HSPB1	2	3	1.584963
P04632	1	1	1	CAPNS1	1	2	1
P04626	1	2	1	ERBB2	1.333333	2.333333	1.222392
P04004	1	1	1	VTN	1	2	1
P03891	2	1	1	ND2	1.333333	2.333333	1.222392
P03886	1	2	2	ND1	1.666667	2.666667	1.415037
P02100	2	2	2	HBE1	2	3	1.584963
P01889	5	6	6	HLA-B	5.666667	6.666667	2.736966
P00387	2	1	1	CYB5R3	1.333333	2.333333	1.222392
P00338	2	2	2	LDHA	2	3	1.584963
O96028	1	1	2	NSD2	1.333333	2.333333	1.222392
O95985	2	1	2	TOP3B	1.666667	2.666667	1.415037
O95625	1	4	3	ZBTB11	2.666667	3.666667	1.874469
O95619	1	1	1	YEATS4	1	2	1
O95602	3	1	3	POLR1A	2.333333	3.333333	1.736966
O95478	5	5	5	NSA2	5	6	2.584963
O95453	3	1	3	PARN	2.333333	3.333333	1.736966
O95400	1	1	1	CD2BP2	1	2	1
O95391	2	1	1	SLU7	1.333333	2.333333	1.222392

O95352	2	1	1	ATG7	1.333333	2.333333	1.222392
O95104	5	6	5	SCAF4	5.333333	6.333333	2.662965
O94992	1	3	3	HEXIM1	2.333333	3.333333	1.736966
O94973	6	5	6	AP2A2	5.666667	6.666667	2.736966
O94972	1	1	1	TRIM37	1	2	1
O94887	2	1	1	FARP2	1.333333	2.333333	1.222392
O94829	1	2	1	IPO13	1.333333	2.333333	1.222392
O94761	2	1	1	RECQL4	1.333333	2.333333	1.222392
O76031	1	1	1	CLPX	1	2	1
O76024	2	2	2	WFS1	2	3	1.584963
O75817	1	1	1	POP7	1	2	1
O75608	1	2	1	LYPLA1	1.333333	2.333333	1.222392
O75600	1	1	1	GCAT	1	2	1
O75460	2	2	2	ERN1	2	3	1.584963
O75427	2	3	3	LRCH4	2.666667	3.666667	1.874469
O75394	1	1	1	MRPL33	1	2	1
O75330	1	3	4	HMMR	2.666667	3.666667	1.874469
O75319	2	1	1	DUSP11	1.333333	2.333333	1.222392
O75175	1	1	1	CNOT3	1	2	1
O75165	6	7	8	DNAJC13	7	8	3
O75152	2	2	2	ZC3H11A	2	3	1.584963
O75147	1	2	3	OBSL1	2	3	1.584963
O60870	3	3	4	KIN	3.333333	4.333333	2.115477
O60645	3	2	3	EXOC3	2.666667	3.666667	1.874469
O60566	1	1	1	BUB1B	1	2	1
O60563	2	1	1	CCNT1	1.333333	2.333333	1.222392
O60524	4	5	4	NEMF	4.333333	5.333333	2.415037
O60508	3	4	2	CDC40	3	4	2
O60427	1	1	2	FADS1	1.333333	2.333333	1.222392
O60294	1	1	1	LCMT2	1	2	1
O60293	3	5	5	ZFC3H1	4.333333	5.333333	2.415037
O60231	2	3	3	DHX16	2.666667	3.666667	1.874469
O43933	1	2	1	PEX1	1.333333	2.333333	1.222392
O43929	2	1	1	ORC4	1.333333	2.333333	1.222392
O43913	3	3	4	ORC5	3.333333	4.333333	2.115477
O43865	4	3	4	AHCYL1	3.666667	4.666667	2.222392
O43847	10	9	7	NRDC	8.666667	9.666667	3.273018
O43824	5	2	5	GTPBP6	4	5	2.321928
O43823	2	2	5	AKAP8	3	4	2
O43818	1	2	1	RRP9	1.333333	2.333333	1.222392
O43776	1	2	3	NARS1	2	3	1.584963
O43760	1	1	1	SYNGR2	1	2	1
O43731	1	1	1	KDELR3	1	2	1
O43504	1	2	1	LAMTOR5	1.333333	2.333333	1.222392
O43414	2	1	2	ERI3	1.666667	2.666667	1.415037
O43395	1	1	1	PRPF3	1	2	1
O43159	2	1	2	RRP8	1.666667	2.666667	1.415037
O43148	3	1	1	RNMT	1.666667	2.666667	1.415037
O15504	1	1	2	NUP42	1.333333	2.333333	1.222392
O15446	4	3	3	POLR1G	3.333333	4.333333	2.115477
O15344	1	1	1	MID1	1	2	1
O15258	2	1	2	RER1	1.666667	2.666667	1.415037
O15160	4	4	3	POLR1C	3.666667	4.666667	2.222392
O15120	2	1	1	AGPAT2	1.333333	2.333333	1.222392
O15116	1	1	1	LSM1	1	2	1
O15091	2	2	2	PRORP	2	3	1.584963

O14975	1	2	1	SLC27A2	1.333333	2.333333	1.222392
O14802	4	2	4	POLR3A	3.333333	4.333333	2.115477
O14773	2	1	1	TPP1	1.333333	2.333333	1.222392
O14730	3	4	5	RIOK3	4	5	2.321928
O14717	1	1	1	TRDMT1	1	2	1
O14617	2	6	5	AP3D1	4.333333	5.333333	2.415037
O14578	1	1	1	CIT	1	2	1
O14561	2	1	2	NDUFAB1	1.666667	2.666667	1.415037
O14545	1	1	1	TRAFD1	1	2	1
O00458	2	2	3	IFRD1	2.333333	3.333333	1.736966
O00311	2	1	2	CDC7	1.666667	2.666667	1.415037
O00303	3	4	3	EIF3F	3.333333	4.333333	2.115477
O00161	1	1	1	SNAP23	1	2	1
B5ME19	3	3	3	EIF3CL	3	4	2
A8MPP1	1	1	1	DDX11L8	1	2	1
A8CG34	3	3	2	POM121C	2.666667	3.666667	1.874469
A6NHR9	3	1	1	SMCHD1	1.666667	2.666667	1.415037
A2RRP1	2	1	1	NBAS	1.333333	2.333333	1.222392
A0A0C4DH	4	3	2	IGHV3-66	3	4	2
